# α-myosin heavy chain lactylation maintains sarcomeric structure and function and alleviates the development of heart failure

**DOI:** 10.1038/s41422-023-00844-w

**Published:** 2023-07-13

**Authors:** Naijin Zhang, Ying Zhang, Jiaqi Xu, Pengbo Wang, Boquan Wu, Saien Lu, Xinxin Lu, Shilong You, Xinyue Huang, Mohan Li, Yuanming Zou, Mengke Liu, Yuanhui Zhao, Guozhe Sun, Wenbin Wang, Danxi Geng, Jingwei Liu, Liu Cao, Yingxian Sun

**Affiliations:** 1 Department of Cardiology, First Hospital of China Medical University, Shenyang, Liaoning China; 2 NHC Key Laboratory of Advanced Reproductive Medicine and Fertility, National Health Commission, China Medical University, Shenyang, Liaoning China; 3 Institute of Health Sciences, China Medical University, Shenyang, Liaoning China; 4 Key Laboratory of Medical Cell Biology, Ministry of Education, Shenyang, Liaoning China; 5 Institute of School of Basic Medicine, China Medical University, Shenyang, Liaoning China; 6 Key Laboratory of Environmental Stress and Chronic Disease Control and Prevention, Ministry of Education, China Medical University, Shenyang, Liaoning China

**Keywords:** Post-translational modifications, Post-translational modifications

## Abstract

The sarcomeric interaction of α-myosin heavy chain (α-MHC) with Titin is vital for cardiac structure and contraction. However, the mechanism regulating this interaction in normal and failing hearts remains unknown. Lactate is a crucial energy substrate of the heart. Here, we identify that α-MHC undergoes lactylation on lysine 1897 to regulate the interaction of α-MHC with Titin. We observed a reduction of α-MHC K1897 lactylation in mice and patients with heart failure. Loss of K1897 lactylation in α-MHC K1897R knock-in mice reduces α-MHC–Titin interaction and leads to impaired cardiac structure and function. Furthermore, we identified that p300 and Sirtuin 1 act as the acyltransferase and delactylase of α-MHC, respectively. Decreasing lactate production by chemical or genetic manipulation reduces α-MHC lactylation, impairs α-MHC–Titin interaction and worsens heart failure. By contrast, upregulation of the lactate concentration by administering sodium lactate or inhibiting the pivotal lactate transporter in cardiomyocytes can promote α-MHC K1897 lactylation and α-MHC–Titin interaction, thereby alleviating heart failure. In conclusion, α-MHC lactylation is dynamically regulated and an important determinant of overall cardiac structure and function. Excessive lactate efflux and consumption by cardiomyocytes may decrease the intracellular lactate level, which is the main cause of reduced α-MHC K1897 lactylation during myocardial injury. Our study reveals that cardiac metabolism directly modulates the sarcomeric structure and function through lactate-dependent modification of α-MHC.

## Introduction

A sarcomere is the basic unit of cardiac structure and contraction.^1^ In sarcomere, myosin tail is packaged together with Titin to form thick filaments. Myosin head and neck domains form a cross bridge. In the presence of calcium ions, the myosin head binds to adenosine triphosphate (ATP), undergoes a conformational change, and pulls on the thin filaments, which results in cardiac contraction.^2,3^ This structure was recently observed by cryo-electron microscopy.^4^ Although myosin is known to dynamically interact with Titin,^5–7^ how they associate and dissociate with each other remains unclear.

While lactate was previously viewed as merely a byproduct of metabolism, it is a vital energy substrate of the heart.^8–10^ Lactate, as an important energy source for mitochondrial respiration and an intercellular signaling molecule, is the fulcrum of metabolic regulation in vivo.^11^ A growing body of evidence has highlighted the role of lactate in cardiac hypertrophy, injury, and heart failure.^12–17^ A recent study showed that lactate is required for cardiac hypertrophic growth in response to pressure overload.^13^ It stabilizes NDRG3 and stimulates extracellular signal-regulated kinase. Lack of lactate prevents cardiomyocytes from developing hypertrophy, but it directly aggravates heart failure under pressure overload. Another work also showed that myocardial lactate levels decreased significantly after adriamycin-induced myocardial injury.^14^ Heart failure promotes lactate secretion from cardiomyocytes into the serum, which leads to excessive lactate accumulation in the serum and is predicted to cause adverse cardiovascular outcomes in patients with heart failure.^15^ Inhibiting lactate efflux from cardiomyocytes by inhibiting monocarboxylate transporter 4 (MCT4) activity effectively alleviates heart failure, suggesting that lactate inside cardiomyocytes plays a protective role.^12^ In addition, lactate consumption almost doubles in failing hearts of patients,^8^ and treatment with lactate analogs alleviates acute heart failure in animal models.^16^ Remarkably, a clinical study further confirmed these effects, showing that treatment with lactate sodium significantly restored cardiac function in patients with acute heart failure.^17^

Recent studies have also demonstrated that lactate may exert its biological activity through protein lactylation, which alters the function of histone or non-histone proteins by post-translationally modifying specific lysine residues.^10,18,19^ It was shown that lactylation of H3K18 promotes the polarization of macrophages from M1 pro-inflammatory phenotype to M2 anti-inflammatory phenotype.^10^ Glis1 facilitates metabolic remodeling from mitochondrial oxidative phosphorylation to glycolysis, which promotes lactate production and thus increases H3K18 lactylation.^18^ In hepatocellular carcinoma (HCC), the enzyme adenylate kinase 2 is inhibited by K28 lactylation, which in turn promotes HCC proliferation and tumorigenesis.^19^ Lactylation at the K673 site of fatty acid synthase inhibits its activity, which mediates the downregulation of lipid accumulation in the liver.^20^ Lactate is a vital energy substrate of the heart, yet relatively little is known about the physiological and pathological roles of lactylation in cardiomyocytes.

Here, we confirmed that lactylation of α-myosin heavy chain (α-MHC), which is encoded by the *Myh6* gene, at lysine 1897 is decreased in mice and patients with heart failure. α-MHC K1897R knock-in (KI) mice exhibit impaired α-MHC–Titin interaction and develop aggravated heart failure. In addition, we further identified that p300 (E1A binding protein, 300 kDa) and Sirtuin 1 (SIRT1) work as acyltransferase and delactylase of α-MHC, respectively. Importantly, excessive lactate efflux and consumption by cardiomyocytes induce a decreased lactate concentration in cardiomyocytes, which is the main cause of the reduction of α-MHC K1897 lactylation and α-MHC–Titin interaction in myocardial injury. Thus, administration of sodium lactate (NALA) or inhibition of lactate efflux could treat mice with heart failure through restoring α-MHC K1897 lactylation and α-MHC–Titin interaction. These results suggest that endogenous lactate is a critical regulator of sarcomeric structure and function.

## Results

### α-MHC K1897 lactylation is decreased in mice and humans with heart failure

**Fig. 1:**
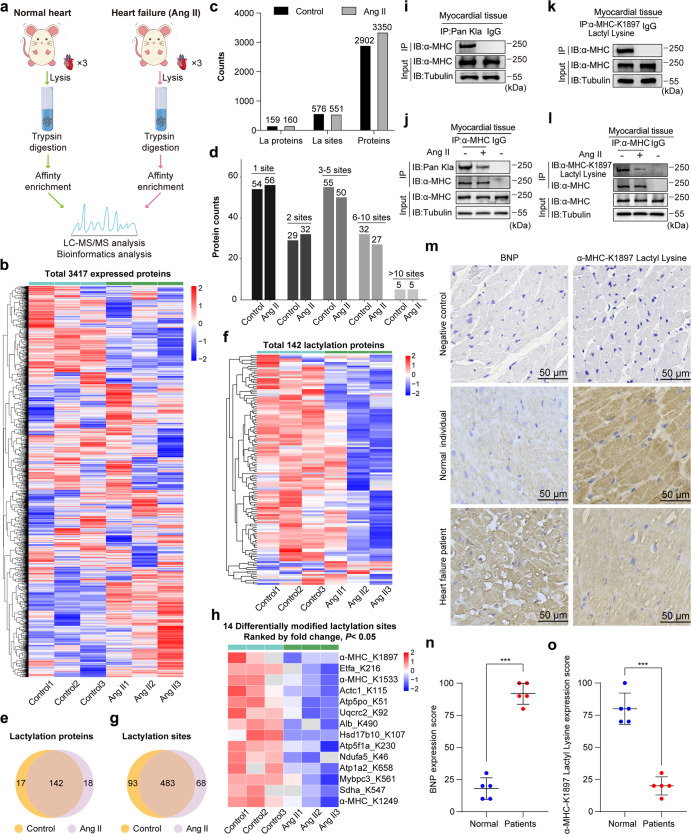
α-MHC K1897 lactylation is decreased in mice and humans with heart failure. **a** Flow chart showing LC-MS/MS analysis and bioinformatic analysis of control and Ang II-induced heart failure mice (*n* = 3 per group). **b** Heatmap of total 3417 expressed proteins of control and Ang II group cardiac samples, displayed individually for each biological replicate. Rows represented individual proteins detected by quantitative MS/MS. **c** Column graph of omics data showing the number of proteins, lactylation-modified proteins and lactylation sites identified by MS/MS. **d** Column graph showing protein quantitative distribution map based on protein lactylation sites identified by MS/MS in control and Ang II groups. **e** Venn diagram showing the shared and specific lactylation-modified proteins in control and Ang II groups. **f** Heatmap of 142 shared lactylation proteins in the control and Ang II-treated mice heart samples, displayed individually for each biological replicate. Rows represented individual proteins detected by quantitative MS/MS. **g** Venn diagram showing the shared and specific lactylation sites. **h** 14 differentially modified lactylation sites of the shared 483 lactylated modified sites in **g**, using cut offs of fold change > 1.5 or fold change < 0.67, and *P* value < 0.05. The selected 14 lactylated modified sites were ranked by fold change. **i** Each WT mouse heart was lysed and immunoprecipitated using anti-Pan Kla antibody or control IgG followed by detection of α-MHC. **k** Each WT mouse heart was lysed and immunoprecipitated using anti-α-MHC K1897 Lactyl Lysine antibody or control IgG followed by detection of α-MHC. **j**, **l** Hearts from control mice and Ang II-treated mice were lysed and immunoprecipitated with anti-α-MHC antibody or control IgG, followed by detection of Pan Kla (**j**) and α-MHC K1897 Lactyl Lysine (**l**). **m** Representative immunohistochemical (IHC) staining of BNP (left) and α-MHC K1897 Lactyl Lysine (right) in the myocardial tissue from normal individuals and heart failure patients. Negative controls were performed with rabbit IgG. **n**, **o** Quantification of the relative BNP and α-MHC K1897 Lactyl Lysine expression score. Data are presented as mean ± standard deviation (SD) (*n* = 5, ***P < 0.001).

A model of heart failure was created in which an osmotic minipump was implanted subcutaneously in mice and angiotensin II (Ang II, 3 mg/kg/day) was infused over 14 days. A previous study showed that this dosage induces heart failure by causing cardiomyocyte injury and apoptosis.^21^ We then collected the hearts of control animals and animals with heart failure and performed proteomics analysis to assess protein lactylation (Fig. 1a, b and Supplementary information, Fig. S1). Among the 2902 proteins identified in the control group, we detected 159 lactylated proteins and 576 lactylation sites. Among the 3350 proteins that were resolved in the Ang II-induced heart failure (Ang II) group, 160 lactylated proteins and 551 lactylation sites were identified (Fig. 1c, d). The control and Ang II group shared 142 lactylated proteins (Fig. 1e, f and Supplementary information, Table S1) and 483 lactylation sites (Fig. 1g and Supplementary information, Table S2). The heatmap analysis showed the lactylation levels of these shared lactylation sites with statistical significance (fold change > 1.5 or fold change < 0.67, and *P* value < 0.05) between the control and Ang II group (Fig. 1h). The substrate site with the greatest change in lactylation was α-MHC K1897 (Fig. 1h and Supplementary information, Fig. S2a, d and Table S2).

Given that the lactylation levels of α-MHC K1533 and K1249 were also decreased in the Ang II group (Fig. 1h and Supplementary information, Fig. S2a–c), we compared the changes in lactylation of α-MHC K1897, K1533, and K1249 sites under physiological and Ang II-treated conditions. Under physiological conditions, compared with wild-type (WT) α-MHC, lactylation was markedly reduced in α-MHC K1897R (a lysine (K) to arginine (R) substitution at position 1897) and slightly reduced in both α-MHC K1533R and α-MHC K1249R (Supplementary information, Fig. S2e), revealing that K1897 lactylation might be a pivotal part in α-MHC lactylation. With Ang II treatment, the degree of lactylation of α-MHC K1897 was the most greatly reduced compared to that of α-MHC K1533 and α-MHC K1249 (Supplementary information, Fig. S2f). These results suggested that the decrease in α-MHC lactylation with Ang II treatment mainly originates from the decrease in K1897 lactylation. Notably, the α-MHC K1897 residue and the surrounding residues were conserved in many species, including *Homo sapiens*, *Mus musculus*, *Rattus norvegicus*, *Nannospalax galili*, *Cricetulus griseus*, *Bos taurus*, and *Xenopus laevis* (Supplementary information, Fig. S2g). Hence, α-MHC K1897 was chosen as the main target in this study.

We next sought to confirm the α-MHC protein lactylation in vivo. Consistent with the proteomics data, we detected α-MHC lactylation in the myocardial tissues in control mice (Fig. 1i) and decreased lactylation of α-MHC in the myocardial tissues in mice with heart failure (Fig. 1j). Next, to study changes in α-MHC K1897 lactylation, we generated an antibody that specifically recognizes this modification. Consistent with the proteomics analysis, using this antibody, we detected α-MHC K1897 lactylation in control mice (Fig. 1k) and its lactylation level was decreased in mice with heart failure (Fig. 1l). Importantly, we found that in human pathological cardiac samples, the concentration of the heart failure marker B-type natriuretic peptide and α-MHC K1897 lactylation were inversely correlated (*P* < 0.001; Fig. 1m–o).

### α-MHC K1897R mutation reduces α-MHC binding with Titin and aggravates heart failure in mice

The interaction between α-MHC and Titin is essential for maintaining myosin stability under physiological conditions, and this interaction is reduced during heart failure.^6^ To test if the α-MHC K1897 lactylation plays a role in facilitating binding to Titin, we assessed the effects of K1897, K1533, and K1249 residues on the interaction between α-MHC and Titin under physiological and Ang II-treated conditions. Our results showed that four fragments of Titin (I106–108, A77–78, A80–82, and A84–86) interacted with α-MHC WT in physiological conditions (Supplementary information, Fig. S3a–c), which is consistent with previous studies.^6,7^ The binding of α-MHC K1897R to the four fragments of Titin was distinctly attenuated (Supplementary information, Fig. S3c–h), whereas the binding of α-MHC K1249R and α-MHC K1533R to the four fragments of Titin was slightly attenuated (Supplementary Information, Fig. S3e–h), revealing that the K1897 site was important in mediating α-MHC–Titin interaction. After treatment with Ang II, this interaction was weakened and the interactions of the four fragments of Titin with α-MHC K1533R and α-MHC K1249R were also attenuated. Moreover, the interactions between the four fragments of Titin and α-MHC K1897R were maintained at a low level and did not reduce obviously with Ang II treatment (Supplementary information, Fig. S3i–l). These results suggested that lactylation at the K1897 residue, but not at the K1533 and K1249 residues, contributes to the reduced α-MHC–Titin interaction by Ang II treatment. Overall, these data demonstrate that α-MHC K1897 lactylation mediates the interaction between α-MHC and Titin both in physiological and pathological conditions.

**Fig. 2:**
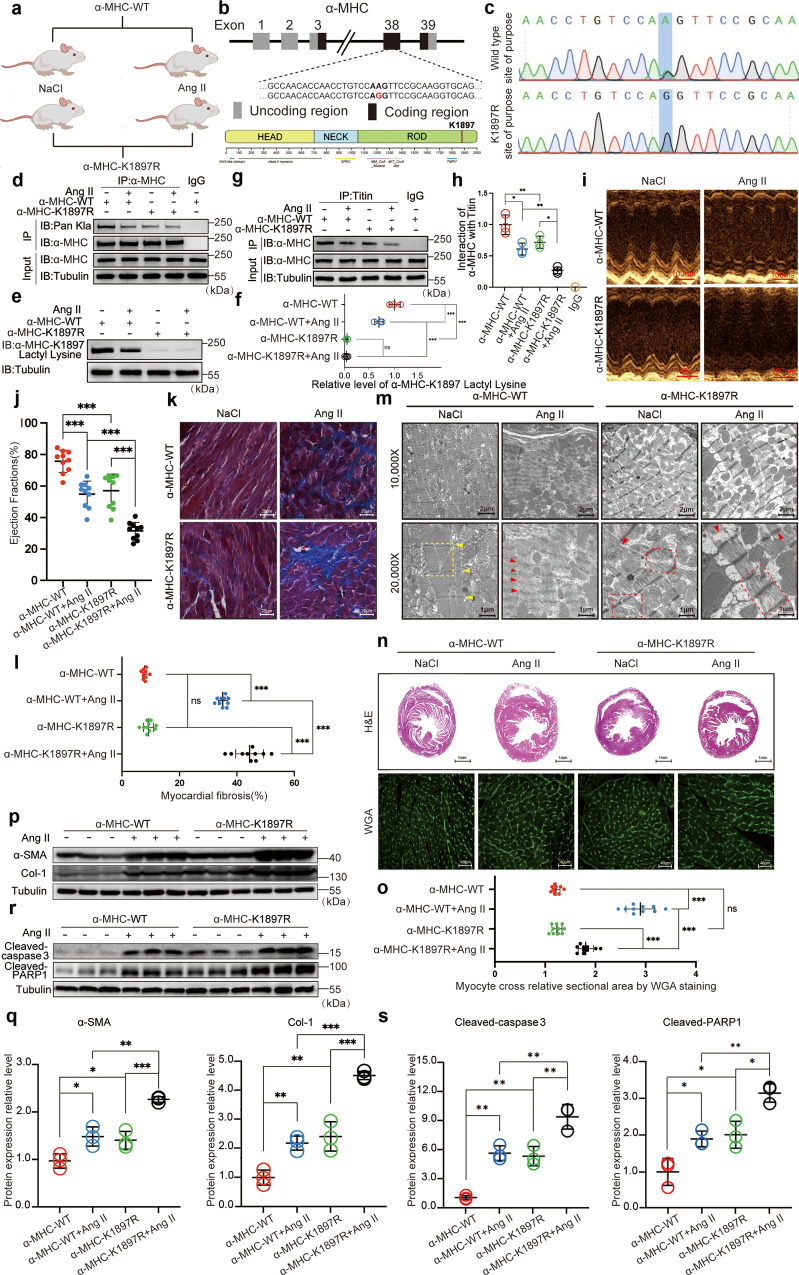
α-MHC K1897R mutation reduces α-MHC binding with Titin and aggravates heart failure in mice. **a** Schematic for experimental intervention in mice. Micro-osmotic pumps were used to deliver NaCl or Ang II subcutaneously to α-MHC K1897R KI and α-MHC WT mice. **b** Structural domains of α-MHC (bottom) and the amino acid change made at the α-MHC mutation site (above). The position of the 1897 mutation is indicated. **c** The nucleotide mutation site in α-MHC K1897R KI mice. **d** Each mouse myocardial tissues indicated in **a** was lysed and immunoprecipitated using anti-α-MHC antibody or control IgG followed by detection of Pan Kla. **e** α-MHC K1897 Lactyl Lysine was detected by western blot (WB) in each mouse myocardial tissues indicated in **a**. **f** Quantification of relative expression level of α-MHC K1897 Lactyl Lysine. Tubulin was used as an internal reference (*n* = 3 per group). **g** Each mouse myocardial tissue indicated in **a** was lysed and immunoprecipitated using anti-Titin antibody or control IgG followed by detection of α-MHC. **h** Quantification of the interaction between α-MHC and Titin. Tubulin was used as an internal reference (*n* = 3 per group). **i** Representative M-mode echocardiogram of WT and α-MHC K1897R KI mice after NaCl or Ang II treatment. **j** Cardiac function was evaluated by EF (*n* = 10 per group). **k**, **l** Masson staining to detect myocardial fibrosis and the quantification of the degree of fibrosis. (*n* = 10 per group). **m** Transmission electron microscope images of myocardial tissue ultrastructure showing myofilaments of WT and α-MHC K1897R KI mice after NaCl or Ang II treatment. The yellow dotted border lines indicate the normal sarcomere structure, and the red dotted border lines indicate the damaged sarcomere structure. The arrowheads indicate Z disk in myocardial tissue. **n** Representative heart sections stained with H&E (top) and WGA (bottom) to detect the presence of hypertrophy in cardiomyocytes. **o** Quantification of relative cardiomyocyte cross-sectional area (*n* = 10 per group). **p**, **q** WB and quantification of fibrosis markers (α-SMA, Col-1) in myocardial tissues. Tubulin was used as an internal reference (*n* = 3 per group). **r**, **s** WB and quantification of cardiac injury markers (cleaved-Caspase3 and cleaved-PARP1) in myocardial tissues. Tubulin was used as an internal reference (*n* = 3 per group). Data are presented as mean ± SD (**f**, **h**, **j**, **l**, **o**, **q**, **s**). Statistical significance was assessed by two-way ANOVA with Bonferroni multiple comparisons test (*P* values adjusted for 6 comparisons; ns, no significance; *P < 0.05; **P < 0.01; ***P < 0.001).

To further assess the role of α-MHC K1897 lactylation in Ang II-induced heart failure, we mutated the lysine AAG codon to the arginine AGG codon and constructed α-MHC K1897R KI mice and administered Ang II to induce heart failure (Fig. 2a–c). Using anti-pan-L-lactyl lysine antibody (Pan Kla), which recognizes lactylated proteins, as well as our α-MHC K1897-specific lactylation antibody, we found that Ang II treatment significantly reduced α-MHC K1897 lactylation. In α-MHC K1897R KI mice, α-MHC K1897 lactylation was undetectable with or without Ang II treatment (Fig. 2d–f). Moreover, the α-MHC–Titin interaction was significantly reduced in α-MHC K1897R KI mice compared with WT mice, both in physiological and pathological conditions (Fig. 2g, h).

We next assessed the physiological effects of the α-MHC K1897R mutation. Echocardiography demonstrated a slight reduction in ejection fraction (EF) and fractional shortening (FS) in α-MHC K1897R KI mice compared with α-MHC WT mice under physiological conditions. Under Ang II conditions, the KI mice displayed a significant impairment in EF and FS compared with WT mice (Fig. 2i, j and Supplementary information, Fig. S8a, f). Masson staining revealed a slight fibrosis in myocardial tissues from the KI mice under physiological conditions, while Ang II-induced myocardial fibrosis was significantly aggravated in the KI mice (Fig. 2k, l). Electron microscopy further confirmed that compared with WT mice, slightly disturbed myofibrillar arrangement and myofibrillar swelling occurred in the KI mice. These changes were significantly aggravated by Ang II stimulation (Fig. 2m). Moreover, we checked hypertrophic changes in the hearts using hematoxylin & eosin staining (H&E) and wheat germ agglutinin staining (WGA). Ang II caused significant hypertrophy in WT mice and mild hypertrophy in the KI mice (Fig. 2n, o). At the molecular level, we evaluated fibrosis markers, including α-smooth muscle actin (SMA) and Col-1, and myocardial injury markers including the apoptosis-related proteins cleaved-PARP1 and cleaved-Caspase3. All these markers were slightly upregulated in the KI mice at baseline and were significantly upregulated in the setting of Ang II-induced heart failure (Fig. 2p–s). Taken together, our results show that downregulation of α-MHC K1897 lactylation leads directly to heart failure without obvious myocardial hypertrophy.

### p300 is the acyltransferase for α-MHC K1897 lactylation

Given that lactylation of α-MHC K1897 plays a key role in heart failure, we set out to determine the enzymatic mechanism of α-MHC lactylation. We tested enzymes, including p300, which was previously shown to catalyze the lactylation of histones as an acyltransferase,^10^ as well as other acyltransferases, including CBP, GCN5, and PCAF.^22^ Only p300 overexpression significantly upregulated α-MHC lactylation (Supplementary information, Fig. S4a).

**Fig. 3:**
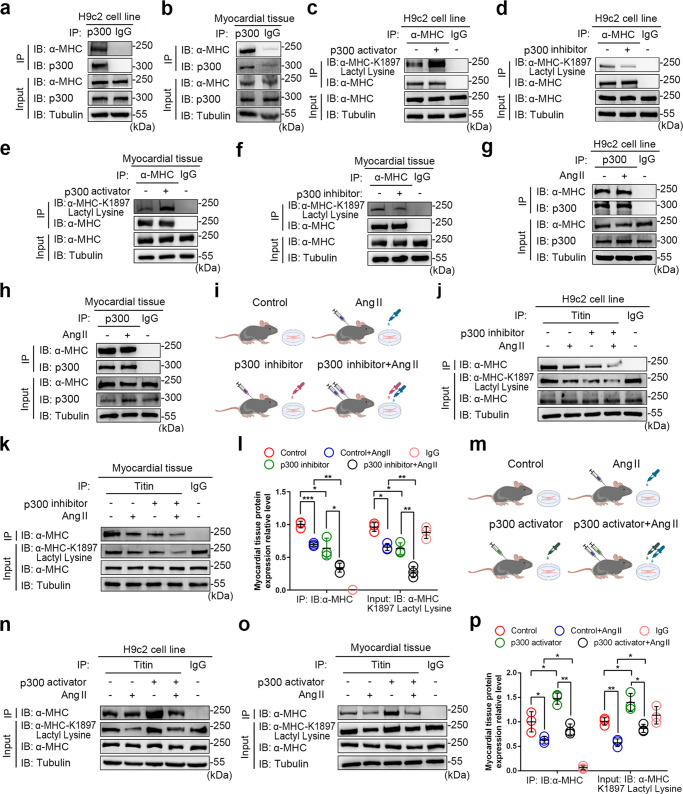
p300 is the acyltransferase for α-MHC K1897 lactylation. **a**–**h** H9c2 cells (**a**) or myocardial tissues (**b**) were lysed and immunoprecipitated using anti-p300 antibody or control IgG, followed by detection of α-MHC. H9c2 cells (**c**) and myocardial tissues (**e**) treated with or without p300 activator were lysed and immunoprecipitated using anti-α-MHC antibody or control IgG, followed by detection of α-MHC K1897 Lactyl Lysine. H9c2 cells (**d**) and myocardial tissues (**f**) treated with or without p300 inhibitor were lysed and immunoprecipitated using anti-α-MHC or control IgG, followed by detection of α-MHC K1897 Lactyl Lysine. H9c2 cells (**g**) and myocardial tissues (**h**) treated with or without Ang II were lysed and immunoprecipitated using anti-p300 antibody or control IgG, followed by detection of α-MHC. **i** Schematic of experimental intervention patterns (control, Ang II, p300 inhibitor, combination of Ang II and p300 inhibitor) in mice and H9c2 cells. **j**, **k** H9c2 cells (**j**) and myocardial tissues (**k**) indicated in **i** were lysed and immunoprecipitated using anti-Titin antibody or control IgG, followed by detection of α-MHC. **l** Quantification of the interaction of α-MHC with Titin and the relative expression level of α-MHC K1897 Lactyl Lysine in **k**. Tubulin was used as an internal reference (*n* = 3 per group). **m** Schematic for experimental intervention patterns (control, Ang II, p300 activator, combination of Ang II and p300 activator) in mice and H9c2 cells. **n**, **o** H9c2 cells (**n**) and myocardial tissue (**o**) indicated in **m** were lysed and immunoprecipitated using anti-Titin antibody or control IgG, followed by detection of α-MHC. **p** Quantification of the interaction of α-MHC with Titin and the expression relative level of α-MHC K1897 Lactyl Lysine in **o**. Tubulin was used as an internal reference (*n* = 3 per group). Data are presented as mean ± SD (**l**, **p**). Statistical significance was assessed by two-way ANOVA with Bonferroni multiple comparisons test (*P* values adjusted for 6 comparisons; *P < 0.05; **P < 0.01; ***P < 0.001).

We then verified the p300–α-MHC interaction by co-immunoprecipitation (Co-IP) in vitro and in vivo (Fig. 3a, b and Supplementary information, Fig. S4b). We found that the α-MHC structural domain Class II, Spec, and mmCoA were required for its interaction with p300 (Supplementary information, Fig. S4c). Moreover, p300 overexpression resulted in enhanced α-MHC lactylation (Supplementary information, Fig. S4d). The degree of α-MHC lactylation and α-MHC K1897 lactylation increased with p300 overexpression, while the α-MHC K1897R lactylation kept low, regardless of p300 expression levels (Supplementary information, Fig. S4e, f). We further validated these results using p300 activator or p300 inhibitor in H9c2 cells and in mouse hearts. In H9c2 cells, p300 activator enhanced α-MHC K1897 lactylation, while p300 inhibitor attenuated α-MHC K1897 lactylation (Fig. 3c, d). When p300 activator or inhibitor was intraperitoneally infused into mice for 2 weeks, similar results were observed (Fig. 3e, f).

We next explored the role of p300 in regulating α-MHC lactylation in heart failure and myocardial injury. We found that p300 expression was upregulated in the cardiac tissues of patients and mice with heart failure, as well as in Ang II-treated H9c2 cells (Supplementary information, Fig. S4g–m), but the interaction between p300 and α-MHC was not changed in H9c2 cells and mouse myocardial tissues, regardless of Ang II stimulation (Fig. 3g, h). To analyze the effects of p300 on α-MHC K1897 lactylation and α-MHC–Titin interaction in Ang II-induced heart failure, we used p300 inhibitor or p300 activator in vitro and in vivo. H9c2 cells and mouse myocardial tissues treated with p300 inhibitor (Fig. 3i) showed reduced baseline α-MHC K1897 lactylation and α-MHC–Titin interaction (Fig. 3j). Ang II reduced α-MHC K1897 lactylation and interaction between α-MHC and Titin. Compared with Ang II stimulation alone, p300 inhibitor combined with Ang II stimulation significantly reduced the degree of α-MHC K1897 lactylation and the interaction of α-MHC with Titin (Fig. 3j–l). In the p300 activator treatment group (Fig. 3m), both in vitro and in vivo results showed that p300 activator enhanced α-MHC K1897 lactylation and α-MHC–Titin interaction, while Ang II treatment alone downregulated both effects. The combination of p300 activator and Ang II stimulation could partially rescue the lactylation of α-MHC K1897 and the α-MHC–Titin interaction when compared with Ang II stimulation alone (Fig. 3n–p).

Altogether, these results suggest that p300 is the acyltransferase for α-MHC K1897 lactylation. However, p300 is not the key player in the reduction in α-MHC K1897 lactylation in heart failure.

### SIRT1 is the delactylase for α-MHC K1897

Our results show that intervention with the p300 acyltransferase activator failed to fully rescue α-MHC K1897 lactylation, as well as α-MHC–Titin interaction in Ang II-induced heart failure mouse model. To further clarify the regulatory mechanism, we sought to identify a relevant α-MHC K1897 delactylase. Given that Sirtuin family proteins are crucial deacylases,^23^ we selected the Sirtuin family enzymes (SIRT1–7) as candidate proteins for delactylazing α-MHC. We found that only the expression of SIRT1 significantly reduced the degree of α-MHC lactylation (Supplementary information, Fig. S5a, b).

**Fig. 4:**
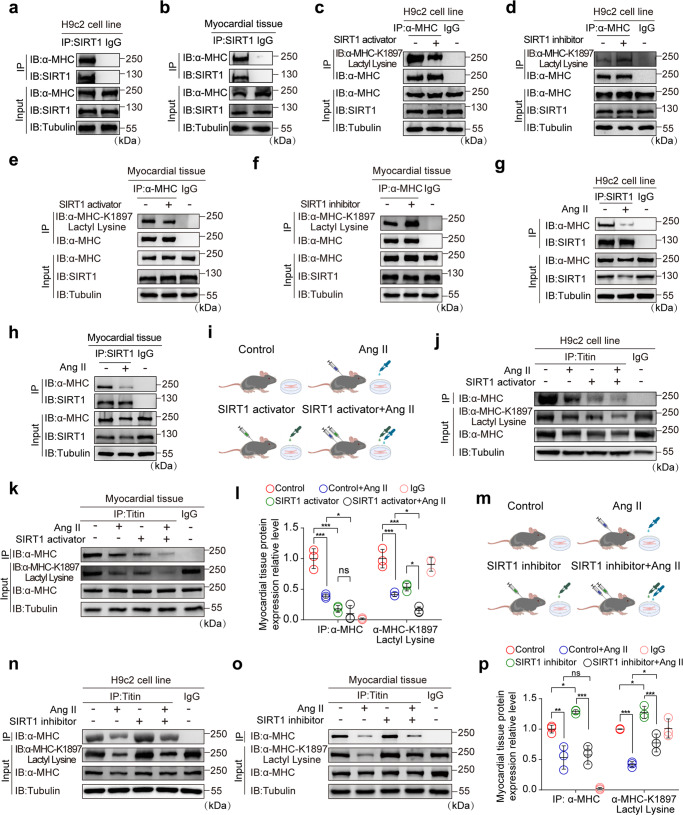
SIRT1 is the delactylase for α-MHC K1897. **a**–**h** H9c2 cells (**a**) or myocardial tissues (**b**) were lysed and immunoprecipitated using anti-SIRT1 antibody or control IgG, followed by detection of α-MHC. H9c2 cells (**c**) and myocardial tissues (**e**) treated with or without SIRT1 activator were lysed and immunoprecipitated using anti-α-MHC antibody or control IgG, followed by detection of α-MHC K1897 Lactyl Lysine. H9c2 cells (**d**) and myocardial tissues (**f**) treated with or without SIRT1 inhibitor were lysed and immunoprecipitated using anti-α-MHC or control IgG, followed by detection of α-MHC K1897 Lactyl Lysine. H9c2 cells (**g**) and myocardial tissue (**h**) treated with or without Ang II were lysed and immunoprecipitated using anti-SIRT1 antibody or control IgG, followed by detection of α-MHC. **i** Schematic of experimental intervention patterns (control, Ang II, SIRT1 activator, combination of Ang II and SIRT1 activator) in mice and H9c2 cells. **j**, **k **H9c2 cells (**j**) and myocardial tissues (**k**) indicated in **i** were lysed and immunoprecipitated using anti-Titin or control IgG, followed by detection of α-MHC. **l** Quantification of the interaction of α-MHC with Titin and the relative expression level of α-MHC K1897 Lactyl Lysine in **k**. Tubulin was used as an internal reference (*n* = 3 per group). **m** Schematic of experimental intervention patterns (control, Ang II, SIRT1 inhibitor, combination of Ang II and SIRT1 inhibitor) in mice and H9c2 cells. **n**, **o** H9c2 cells (**n**) and myocardial tissues (**o**) indicated in **m** were lysed and immunoprecipitated using anti-Titin or control IgG, followed by detection of α-MHC. **p** Quantification of the interaction of α-MHC with Titin and the relative expression level of α-MHC K1897 Lactyl Lysine in **o**. Tubulin was used as an internal reference (*n* = 3 per group). Data are presented as mean ± SD (**l**, **p**). Statistical significance was assessed by two-way ANOVA with Bonferroni multiple comparisons test (*P* values adjusted for 6 comparisons; ns, no significance; *P < 0.05; **P < 0.01; ***P < 0.001).

We subsequently verified the interaction between SIRT1 and α-MHC in vivo and in vitro by immunoprecipitation (Fig. 4a, b and Supplementary information, Fig. S5c). We identified the structural domains of α-MHC interacting with SIRT1 as mmCoA, MIT-CorA, and TMPIT (Supplementary information, Fig. S5d). Consistent with the above results, overexpression of SIRT1 reduced α-MHC lactylation (Supplementary information, Fig. S5e, f). Similarly, treating cells with SIRT1 activator decreased α-MHC lactylation, whereas treatment with SIRT1 inhibitor had an opposite effect (Supplementary information, Fig. S5g, h). Next, we used the anti-Pan Kla antibody and anti-α-MHC K1897 lactyl Lysine antibody to investigate SIRT1-mediated delactylation of α-MHC K1897. Overexpression of SIRT1 or addition of SIRT1 activator reduced α-MHC K1897 lactylation (Supplementary information, Fig. S5i–k), whereas SIRT1 inhibitor augmented K1897 lactylation (Supplementary information, Fig. S5l). We observed similar results in H9c2 cells treated with SIRT1 activator or inhibitor (Fig. 4c, d). Following 2 weeks of intraperitoneal administration of SIRT1 activator or inhibitor, myocardial examination revealed similar results (Fig. 4e, f).

To further support the role of SIRT1-regulated α-MHC lactylation, we found that SIRT1 expression was decreased in cardiac tissues of patients and mice with heart failure, as well as in Ang II-induced H9c2 cells (Supplementary information, Fig. S5m–s). Ang II stimulation attenuated the interaction of SIRT1 with α-MHC in cardiomyocytes and myocardial tissue (Fig. 4g, h). To investigate the effect of SIRT1 on α-MHC K1897 lactylation and the α-MHC–Titin interaction in Ang II-induced heart failure, cardiomyocytes were treated in vitro and in vivo with SIRT1 activator or SIRT1 inhibitor. As expected, in H9c2 cells and mouse cardiac tissue, SIRT1 activator or Ang II treatment downregulated α-MHC K1897 lactylation and reduced α-MHC–Titin interaction. When compared to the Ang II stimulation group, the administration of Ang II together with the SIRT1 activator attenuated α-MHC K1897 lactylation and reduced α-MHC–Titin interaction (Fig. 4i–l). In contrast, the addition of SIRT1 inhibitor resulted in the upregulation of α-MHC K1897 lactylation and increased α-MHC–Titin interaction. However, compared with Ang II stimulation alone, Ang II stimulation together with the addition of SIRT1 inhibitor partially restored α-MHC K1897 lactylation and α-MHC–Titin interaction (Fig. 4m–p).

These results demonstrate that SIRT1 is the delactylase for α-MHC K1897. However, SIRT1 is not essential in mediating α-MHC K1897 delactylation in heart failure.

### Inhibition of LDHA activity reduces the lactate concentration and inhibits α-MHC K1897 lactylation

LDHA was reported to regulate the amount of lactate in cells.^10^ We speculated that LDHA may act as another important regulatory factor to maintain the degree of α-MHC K1897 lactylation. Indeed, LDHA overexpression increased α-MHC lactylation (Supplementary information, Fig. S6a), whereas LDHA depletion (Supplementary information, Fig. S6b) or application of LDHA inhibitor (Supplementary information, Fig. S6c) reduced α-MHC lactylation. LDHA overexpression upregulated α-MHC K1897 lactylation (Supplementary information, Fig. S6d). However, compared with α-MHC WT, α-MHC K1897R lactylation was significantly reduced, regardless of LDHA overexpression (Supplementary information, Fig. S6e). LDHA knockdown reduced lactylation of α-MHC WT, while α-MHC K1897R lactylation remained low, with or without LDHA knockdown (Supplementary information, Fig. S6f). Similarly, the LDHA inhibitor had the same effect as LDHA knockdown did, both of which reduced α-MHC K1897 lactylation (Supplementary information, Fig. S6g).

**Fig. 5:**
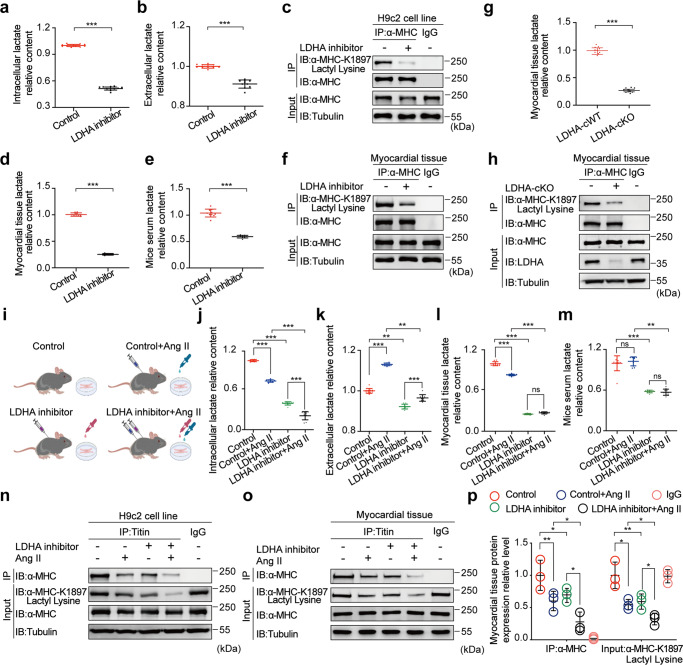
Inhibition of LDHA activity reduces the lactate concentration and inhibits α-MHC K1897 lactylation. **a**, **b** Relative content of intracellular (**a**) and extracellular (**b**) lactate in H9c2 cells after DSMO or LDHA inhibitor treatment (*n* = 10 per group). **c** H9c2 cells stimulated with or without LDHA inhibitor were lysed and immunoprecipitated using anti-α-MHC antibody or control IgG, followed by detection of α-MHC K1897 Lactyl Lysine. **d**, **e** Relative content of lactate in myocardial tissues (**d**) and serum (**e**) in mice after control or LDHA inhibitor treatment (*n* = 10 per group). **f**, **h** Mouse myocardial tissues treated by LDHA inhibitor (**f**) or genetic (LDHA-cKO) manipulation (**h**) were lysed and immunoprecipitated using anti-α-MHC antibody or control IgG, followed by detection of α-MHC K1897 Lactyl Lysine. **g** Relative content of lactate in myocardial tissues in LDHA-cKO and LDHA cWT mice (*n* = 10 per group). **i** Schematic for experimental intervention patterns (control, Ang II, LDHA inhibitor, combination of Ang II and LDHA inhibitor) in mice and H9c2 cells. **j**, **k** Relative content of intracellular (**j**) and extracellular (**k**) lactate in cardiomyocytes after control, Ang II, LDHA inhibitor or combination of Ang II and LDHA inhibitor (*n* = 10 per group). **l**, **m** Relative content of lactate in myocardial tissues (**l**) and serum (**m**) in mice after the indicated treatments (*n* = 10 per group). **n**, **o** H9c2 cells (**n**) and myocardial tissues (**o**) treated with control, Ang II, LDHA inhibitor or combination of Ang II and LDHA inhibitor were lysed and immunoprecipitated using anti-Titin antibody or control IgG, followed by detection of α-MHC. **p** Quantification of the interaction of α-MHC with Titin and the relative expression level of α-MHC K1897 Lactyl Lysine in **o**. Tubulin was used as an internal reference (*n* = 3 per group). Data are represented as mean ± SD (**a**, **b**, **d**, **e**, **g**). Statistical significance was assessed by Student’s *t*-test (***P < 0.001). Data are presented as mean ± SD (**j**–**m**, **p**). Statistical significance was assessed by two-way ANOVA with Bonferroni multiple comparisons test (*P* values adjusted for 6 comparisons; ns, no significance; *P < 0.05; **P < 0.01; ***P < 0.001).

Based on the above results, we examined the regulatory mechanism of LDHA on α-MHC by assessing the effect of LDHA inhibitor or knockout of *LDHA*. As expected, lactate concentration decreased after application of LDHA inhibitor (Fig. 5a, b). LDHA inhibitor also reduced α-MHC K1897 lactylation in cardiomyocytes (Fig. 5c). The lactate concentration in myocardial tissues and serum was reduced after treatment with LDHA inhibitor (Fig. 5d, e), and α-MHC K1897 lactylation was reduced (Fig. 5f). Furthermore, using myocardium-specific *LDHA* knockout mice (LDHA-cKO mice), we observed a decreased lactate concentration in myocardial tissue (Fig. 5g), as well as reduced K1897 lactylation (Fig. 5h).

The effects of LDHA on α-MHC K1897 lactylation were next explored in Ang II-induced heart failure (Fig. 5i). In H9c2 cells, LDHA inhibitor decreased lactate concentration in the cells and medium. Ang II stimulation resulted in a reduction in cellular lactate concentration, while its concentration in the medium increased. Compared with Ang II treatment alone, Ang II combined with LDHA inhibitor resulted in a decreased lactate concentration in both cells and medium (Fig. 5j, k). Similarly, in mouse myocardial tissue, LDHA inhibitor decreased the lactate concentration in myocardial tissues and serum, whereas Ang II decreased the lactate concentration in tissue. Compared with Ang II alone, when Ang II was combined with the LDHA inhibitor, the lactate concentration was decreased in tissues and serum (Fig. 5l, m). At the molecular level, the LDHA inhibitor or Ang II treatment decreased α-MHC K1897 lactylation and reduced the interaction of α-MHC with Titin in cardiomyocytes and in mouse cardiac tissues. Compared with the Ang II treatment alone, when Ang II was combined with the LDHA inhibitor, α-MHC K1897 lactylation and α-MHC–Titin interaction in cells and tissues were further decreased (Fig. 5n–p).

### LDHA-cKO mice decreases α-MHC K1897 lactylation and aggravates heart failure

**Fig. 6:**
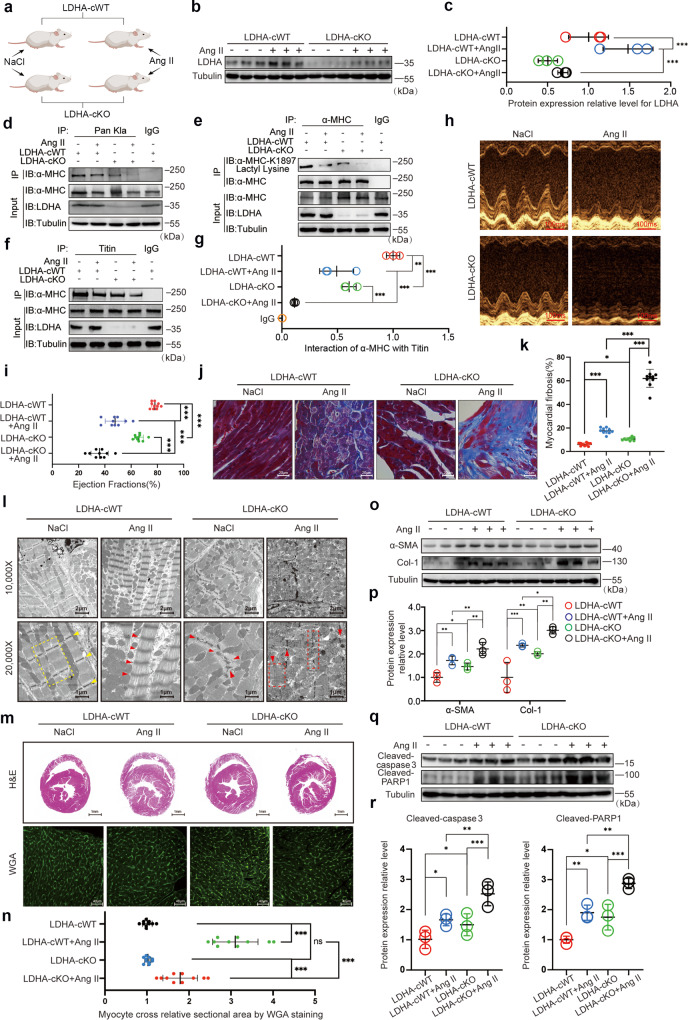
LDHA-cKO mice decreases α-MHC K1897 lactylation and aggravates heart failure. **a** Schematic for experimental intervention patterns in mice. Micro-osmotic pumps were used to deliver NaCl or Ang II subcutaneously to LDHA-cKO and LDHA-cWT mice. **b** WB assay assessed LDHA expression levels in myocardial tissues from LDHA-cWT and LDHA-cKO mice after NaCl or Ang II treatment. **c** Quantification analysis of LDHA expression. **d** Mouse myocardial tissues indicated in **a** were lysed and immunoprecipitated using anti-Pan kla antibody or control IgG, followed by detection of α-MHC. **e** Mouse myocardial tissues indicated in **a** were lysed and immunoprecipitated using anti-α-MHC antibody or control IgG, followed by detection of α-MHC K1897 Lactyl Lysine. **f** Mouse myocardial tissues indicated in **a** were lysed and immunoprecipitated using anti-Titin antibody or control IgG, followed by detection of α-MHC. **g** Quantification of the interaction between α-MHC and Titin. Tubulin was used as an internal reference (*n* = 3 per group). **h** Representative M-mode echocardiogram of LDHA-cWT and LDHA-cKO mice after NaCl or Ang II treatment. **i** Cardiac function was evaluated by EF (*n* = 10 per group). **j** Masson staining to detect myocardial fibrosis. **k** Quantification of the fibrotic area (*n* = 10 per group). **l** Transmission electron microscope photos of heart tissue ultrastructure in LDHA-cWT and cKO mice after NaCl or Ang II treatment. The yellow dotted border lines indicate the normal sarcomere structure, and the red dotted border lines indicate the damaged sarcomere structure. The arrowheads indicate Z disk in myocardial tissues. **m** Representative heart sections stained with H&E (top) and WGA (bottom) to detect the presence of hypertrophy in cardiac myocytes. **n** Quantification of relative cardiomyocyte cross-sectional area (*n* = 10 per group). **o**, **p** WB and quantification of fibrosis marker (α-SMA, Col-1) levels in myocardial tissues. Tubulin was used as an internal reference (*n* = 3 per group). **q**, **r** WB and quantification of cardiac injury marker (cleaved-Caspase3 and cleaved-PARP1) levels in myocardial tissues. Tubulin was used as an internal reference (*n* = 3 per group). Data are presented as mean ± SD (**c**, **g**, **i**, **k**, **n**, **p**, **r**). Statistical significance was assessed by two-way ANOVA with Bonferroni multiple comparisons test (*P* values adjusted for 6 comparisons; ns, no significance; *P < 0.05; **P < 0.01; ***P < 0.001).

To further elucidate the role of LDHA in Ang II-induced heart failure, we administered Ang II into LDHA-cKO mice (Fig. 6a). LDHA expression slightly increased in the hearts of LDHA-cWT mice under Ang II stimulation; however, LDHA expression remained low in LDHA-cKO mice, regardless of whether Ang II was administered (Fig. 6b, c). We observed decreased α-MHC K1897 lactylation in LDHA-cKO mice compared with LDHA-cWT mice under physiological conditions. In Ang II-induced heart failure, LDHA-cKO mice displayed a significant reduction in α-MHC K1897 lactylation compared with LDHA-cWT mice (Fig. 6d, e). Correspondingly, compared with LDHA-cWT mice, LDHA-cKO mice showed a weaker α-MHC–Titin interaction, which was significantly decreased in Ang II-induced heart failure (Fig. 6f, g).

We next explored the physiological effect of LDHA in LDHA-cKO mice. Echocardiography demonstrated slightly reduced EF and FS in LDHA-cKO mice compared with those in LDHA-cWT mice under baseline conditions. Compared with LDHA-cWT mice, the EF and FS showed a significant decline in LDHA-cKO mice treated with Ang II (Fig. 6h, i and Supplementary information, Fig. S8b, g). Masson staining showed that cardiomyocyte fibrosis in LDHA-cKO mice slightly increased compared with LDHA-cWT mice under baseline conditions, and compared with LDHA-cWT mice, Ang II stimulation significantly increased fibrosis in LDHA-cKO mice (Fig. 6j, k). Transmission electron microscopy showed slightly disturbed myofibrillar arrangement and myofibrillar swelling in LDHA-cKO mice compared with those in LDHA-cWT mice, and significantly disturbed myofibrillar arrangement and myofibrillar swelling were observed in LDHA-cKO mice received Ang II treatment (Fig. 6l). In addition, H&E staining and WGA staining revealed that LDHA-cWT mice exhibited significant hypertrophy after Ang II stimulation, whereas LDHA-cKO mice merely exhibited mild hypertrophy (Fig. 6m, n). At the molecular level, we observed a slight upregulation of fibrosis and injury indicators in LDHA-cKO mice compared with LDHA-cWT mice under baseline conditions. Following Ang II administration in LDHA-cKO mice, significant increases in α-SMA and Col-1 expression were observed, which is consistent with the result of fibrosis in the hearts compared with LDHA-cWT mice. Furthermore, apoptosis-related proteins cleaved-PARP1 and cleaved-Caspase3 were upregulated (Fig. 6o–r). These results suggest that loss of *LDHA* in LDHA-cKO reduces the lactate concentration in myocardial tissue and downregulates α-MHC K1897 lactylation, which in turn reduces α-MHC–Titin interaction and exacerbates Ang II-induced heart failure, without obvious myocardial hypertrophy.

Importantly, slightly increased expression of LDHA in mice with heart failure suggests that LDHA may not be the core manipulator in the reduction in α-MHC K1897 lactylation during heart failure.

### NALA increases α-MHC K1897 lactylation and prevents heart failure

**Fig. 7:**
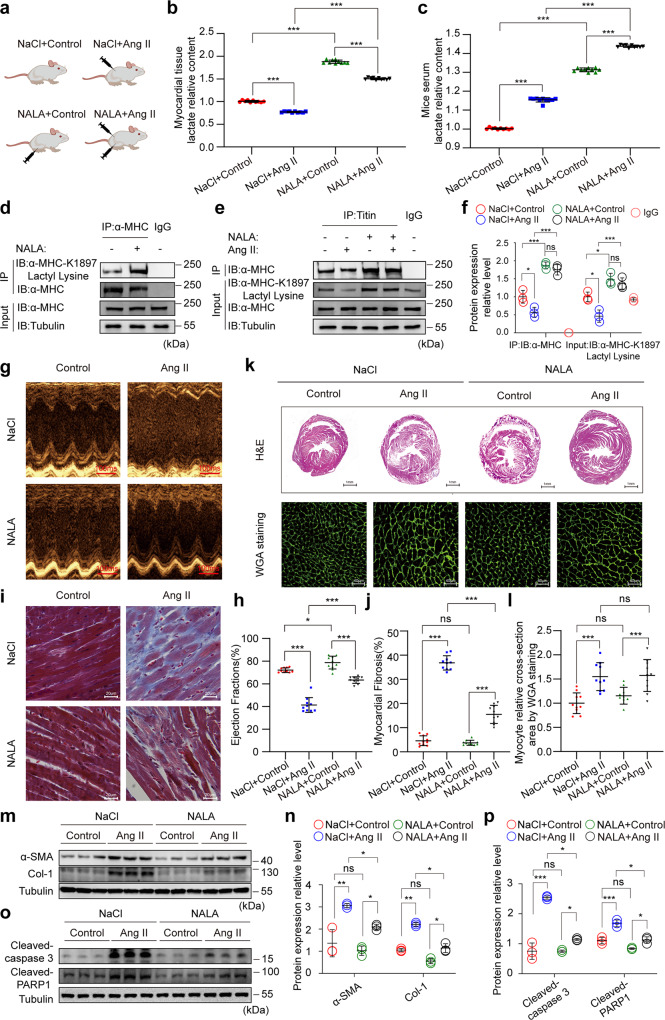
NALA increases lactylation of α-MHC K1897 and prevents heart failure. **a** Schematic for experimental intervention patterns (control/Ang II/NALA/ combination of Ang II and NALA) in mice. **b**, **c** Relative content of lactate in myocardial tissues (**b**) and serum (**c**) in mice after the indicated treatments (*n* = 10 per group). **d** Mouse myocardial tissues treated with or without NALA were lysed and immunoprecipitated using anti-α-MHC antibody or control IgG, followed by detection of α-MHC K1897 Lactyl Lysine. **e** Mouse myocardial tissues indicated in **a** were lysed and immunoprecipitated using anti-Titin antibody or control IgG, followed by detection of α-MHC. **f** Quantification of the interaction between α-MHC and Titin. Tubulin was used as an internal reference (*n* = 3 per group). **g** Representative M-mode echocardiogram of mice after the indicated treatments. **h** Cardiac function was evaluated by EF (*n* = 10 per group). **i** Masson staining was used to show myocardial fibrosis. **j** Quantification of the fibrotic area (*n* = 10 per group). **k** Representative heart sections stained with H&E (top) and WGA (bottom) to detect the presence of hypertrophy in cardiac myocytes. **l** Quantification of relative cardiomyocyte cross-sectional area (*n* = 10 per group). **m**, **n** WB and quantification of fibrosis marker (α-SMA, Col-1) levels in myocardial tissues. Tubulin was used as an internal reference (*n* = 3 per group). **o**, **p** WB and quantification of cardiac injury marker (cleaved-Caspase3 and cleaved-PARP1) levels in myocardial tissues. Tubulin was used as an internal reference (*n* = 3 per group). Data are presented as mean ± SD (**b**, **c**, **f**, **h**, **j**, **l**, **n**, **p**). Statistical significance was assessed by two-way ANOVA with Bonferroni multiple comparisons test (*P* values adjusted for 6 comparisons; ns, no significance; *P < 0.05; **P < 0.01; ***P < 0.001).

Interestingly, though LDHA expression was slightly increased in heart failure, lactate concentration in H9c2 cells and myocardial tissues was decreased. However, we found that lactate concentration was increased in the H9c2 cell culture medium and mouse serum (Supplementary information, Fig. S7a–d). We hypothesized that a diminished lactate concentration in cardiomyocytes is a major mediator of decreased α-MHC K1897 lactylation. To investigate this hypothesis, we evaluated the effects of NALA administration in H9c2 cells and mice, which were then grouped according to the administrations of control treatment, Ang II infusion, NALA infusion, and the combination of Ang II and NALA infusion (Fig. 7a). Regardless of Ang II stimulation, administration of NALA significantly increased the lactate concentration in vitro, both intracellularly and extracellularly, as well as in cardiac tissue and serum in vivo (Fig. 7b, c and Supplementary information, Fig. S7e, f). Moreover, NALA significantly upregulated α-MHC K1897 lactylation in H9c2 cells and mouse myocardial tissues (Fig. 7d and Supplementary information, Fig. S7g). Surprisingly, we found that under baseline conditions and Ang II-induced heart failure, treatment with NALA caused significant increase in α-MHC K1897 lactylation and α-MHC–Titin interaction (Fig. 7e, f and Supplementary information, Fig. S7h).

Remarkably, NALA slightly increased cardiac EF and FS in mice under baseline conditions and significantly improved the cardiac EF and FS in Ang II-induced heart failure (Fig. 7g, h and Supplementary information, Fig. S8c, h). In addition, treatment with NALA did not affect the degree of myocardial fibrosis in mice under baseline conditions. Compared with the control treatment, NALA administration showed a significant protective effect from myocardial fibrosis in mice treated with Ang II (Fig. 7i, j). Compared with the control treatment, NALA treatment had no obvious effect on myocardial hypertrophy in mice with or without Ang II stimulation (Fig. 7k, l). In addition, NALA had no effect on the expressions of the fibrosis markers α-SMA and Col-1, or the apoptotic markers cleaved-Caspase3 and cleaved-PARP1, in mouse myocardial tissues under baseline conditions. However, in Ang II-induced heart failure, the expressions of α-SMA, Col-1, cleaved-Caspase3, and cleaved-PARP1 were obviously declined after NALA administration (Fig. 7m–p).

These results suggest that lactate deficiency in cardiomyocytes and myocardial tissues may be the core mediator of reduction in α-MHC K1897 lactylation, which can be rescued by NALA infusion. NALA significantly abrogates heart failure by enhancing α-MHC K1897 lactylation both in vitro and in vivo.

### The α-MHC K1897R mutation partially abolishes the protective effect of NALA against Ang II-induced heart failure

**Fig. 8:**
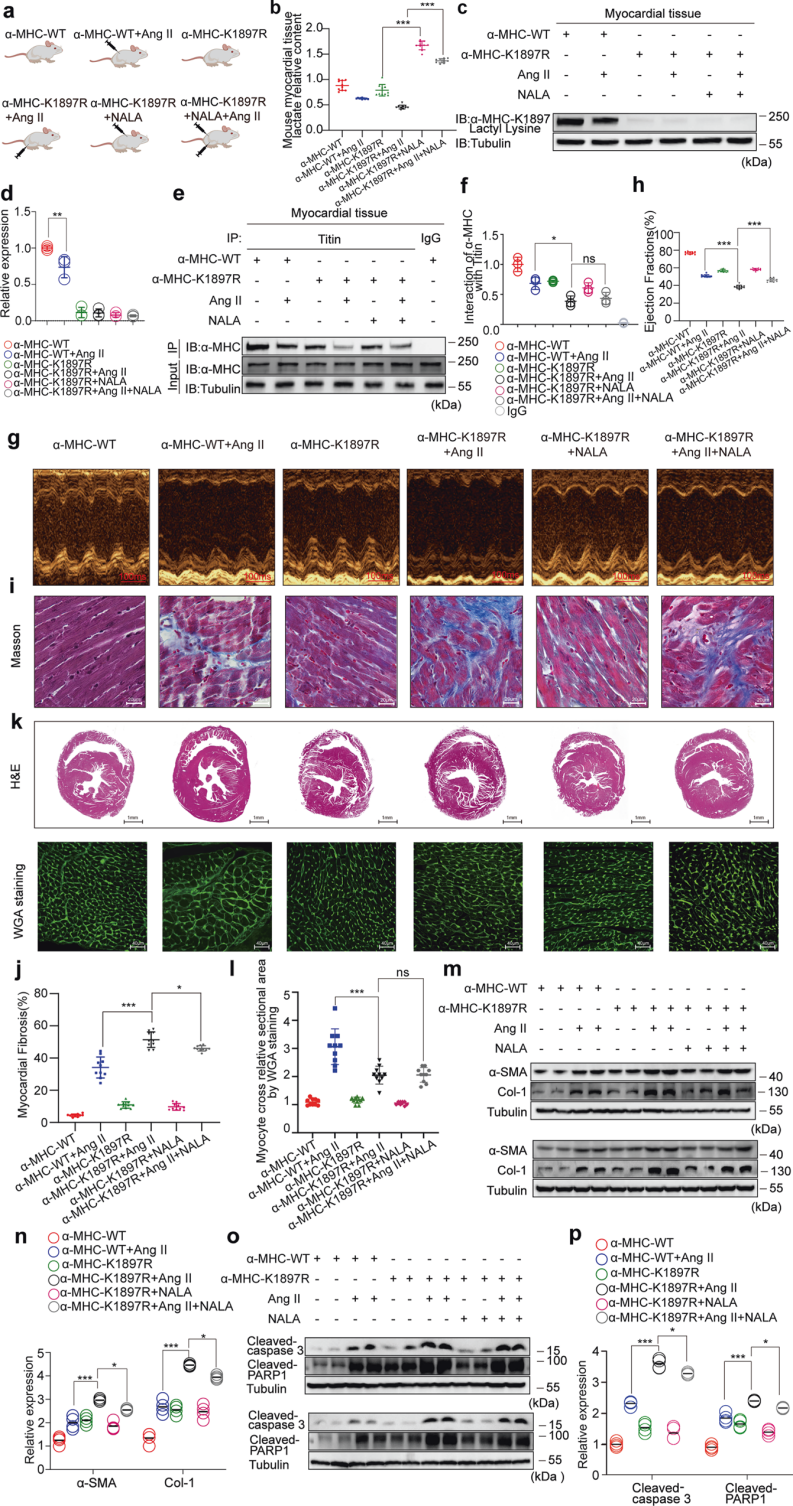
The α-MHC K1897R mutant partially abolishes the protective effect of NALA against Ang II-induced heart failure. **a** Schematic for experimental intervention patterns. α-MHC WT mice were given control treatment or Ang II infusion. α-MHC K1897R KI mice were given control treatment/Ang II infusion/NALA infusion/combined Ang II and NALA infusion. **b** Relative content of myocardial tissue lactate in mice after the indicated treatment (*n* = 10 per group). **c**, **d** Representative WB and quantification of α-MHC K1897 Lactyl Lysine in myocardial tissues from mice after the indicated treatment. Tubulin was used as an internal reference (*n* = 3 per group). **e**, **f** Immunoprecipitation using anti-Titin antibody or control IgG (**e**), followed by detection of α-MHC. Quantification of the interaction of α-MHC with Titin (**f**). Tubulin was used as an internal reference (*n* = 3 per group). **g** Representative M-mode echocardiogram of mice after the indicated treatment. **h** Cardiac function was evaluated by EF (*n* = 10 per group). **i** Masson staining to detect myocardial fibrosis. **j** Quantification of the fibrotic area (*n* = 10 per group). **k** Representative heart sections stained with H&E (top) and WGA (bottom) to detect the presence of hypertrophy in cardiomyocytes. **l** Quantitation of relative cardiomyocyte cross-sectional area (*n* = 10 per group). **m**, **n** WB and quantification of fibrosis marker (α-SMA, Col-1) levels in myocardial tissues. Tubulin was used as an internal reference (*n* = 4 per group). **o**, **p** WB and quantification of cardiac injury indicator (cleaved-Caspase3 and cleaved-PARP1) levels in myocardial tissue, Tubulin was used as an internal reference (*n* = 4 per group). **b**, **d**, **f**, **h**, **j**, **l**, **n**, **p** Statistical significance was assessed by two-way ANOVA with Bonferroni multiple comparisons test (*P* values adjusted for 10 comparisons; ns no significance; *P < 0.05; **P < 0.01; ***P < 0.001).

Next, we administered NALA in Ang II-induced heart failure in α-MHC K1897R KI mice. WT mice underwent control treatment or Ang II treatment, whereas α-MHC K1897R KI mice were grouped to receive control treatment, Ang II treatment, NALA treatment, and Ang II and NALA combined treatment (Fig. 8a). The results showed that after NALA administration, the lactate concentration in mouse myocardial tissues was increased significantly, regardless in Ang II-treated heart failure mice or the α-MHC K1897R KI mice (Fig. 8b). We found that Ang II treatment significantly reduced α-MHC K1897 lactylation in α-MHC WT mice; however, in α-MHC K1897R KI mice, α-MHC K1897 lactylation was undetectable with or without Ang II or NALA treatment (Fig. 8c, d). In addition, α-MHC K1897R KI mice showed a significant reduction in α-MHC–Titin interaction, which was caused by Ang II, compared with the α-MHC WT group. However, NALA merely slightly rescued the reduction in α-MHC–Titin interaction caused by Ang II in α-MHC K1897R KI mice (Fig. 8e, f).

We next explored the physiological effects of NALA in Ang II-induced heart failure in α-MHC K1897R KI mice. Compared with α-MHC WT mice, the α-MHC K1897R KI mice showed a greater reduction in EF and FS induced by Ang II. However, NALA merely partially rescued the decreased EF and FS induced by Ang II in α-MHC K1897R KI mice (Fig. 8g, h and Supplementary information, Fig. S8d, i). Moreover, compared with α-MHC WT mice, the α-MHC K1897R KI mice showed significant aggravation of Ang II-induced myocardial fibrosis, but NALA merely partially rescued this phenotype (Fig. 8i, j). With Ang II treatment, the α-MHC WT mice displayed obvious hypertrophy, but the α-MHC K1897R KI mice merely displayed mild hypertrophy. Again, NALA had no obvious influence on this phenotype (Fig. 8k, l). Finally, with Ang II treatment, our WB assay showed that compared with α-MHC WT mice, expressions of α-SMA, Col-1, cleaved-Caspase3, and cleaved-PARP1 in the myocardial tissue of α-MHC K1897R KI mice increased significantly, but NALA merely partially inhibited these biomarker expressions (Fig. 8m–p).

In sum, NALA had a therapeutic effect on heart failure in α-MHC WT mice, but α-MHC K1897R partially abolished the protective role of NALA against Ang II-induced heart failure in vivo.

### MCT4 inhibitor VB124 increases α-MHC K1897 lactylation and prevents heart failure, and the α-MHC K1897R mutation partially abolishes the protective role of VB124 against Ang II-induced heart failure

**Fig. 9:**
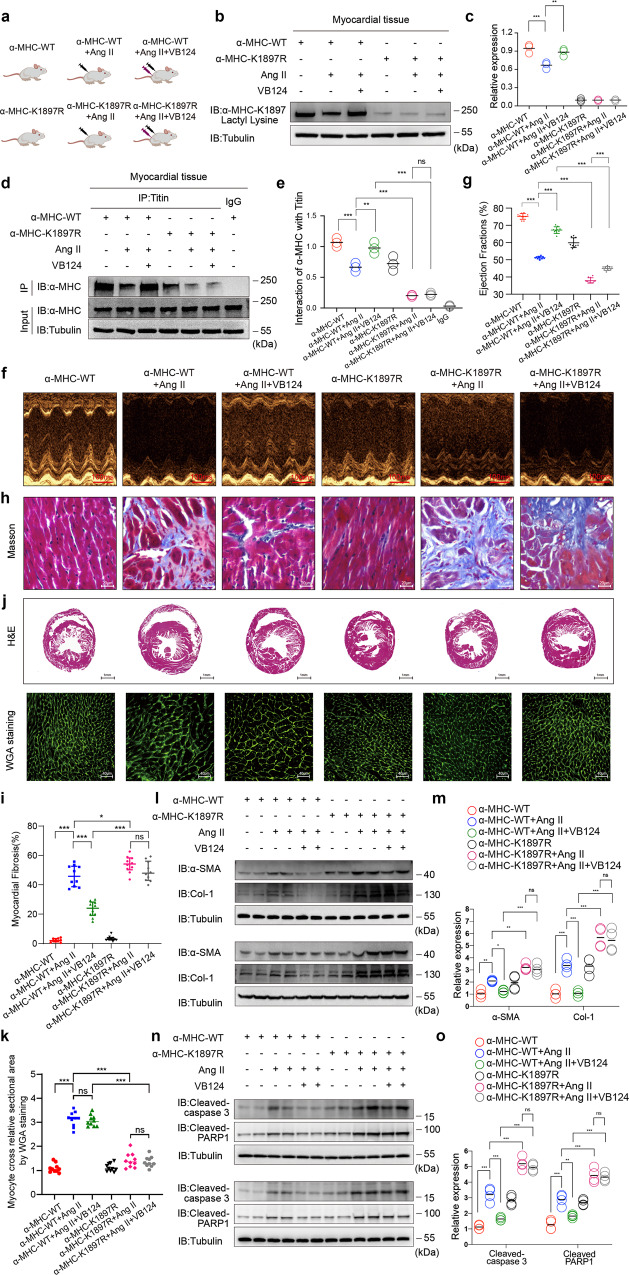
MCT4 inhibitor VB124 increases α-MHC K1897 lactylation and prevents heart failure, and the α-MHC K1897R mutation partially abolishes the protective effect of VB124 against Ang II-induced heart failure. **a** Schematic diagram for experimental intervention patterns. α-MHC WT and α-MHC K1897R KI mice were given control treatment, Ang II infusion or combined Ang II and VB124 infusion for 2 weeks. **b**, **c** Representative WB image and quantification of α-MHC K1897 Lactyl Lysine in myocardial tissue from mice after the indicated treatments. Tubulin was used as an internal reference (*n* = 3 per group). **d**, **e** Immunoprecipitation using anti-Titin antibody or control IgG followed by detection of α-MHC (**d**). Quantification of the interaction of α-MHC with Titin (**e**). Tubulin was used as an internal reference (*n* = 3 per group). **f** Representative mouse M-mode echocardiogram after the indicated treatment. **g** Cardiac function was evaluated by EF (*n* = 10 per group). **h** Masson staining to detect myocardial fibrosis. **i** Quantification of the fibrotic area (*n* = 10 per group). **j** Representative heart sections stained with H&E (top) and WGA (bottom) to detect the presence of hypertrophy in cardiac myocytes. **k** Quantitation of relative cardiomyocyte cross-sectional area (*n* = 10 per group). **l**, **m** WB and quantification of fibrosis marker (α-SMA, Col-1) levels in myocardial tissues. Tubulin was used as an internal reference (*n* = 4 per group). **n**, **o** WB and quantification of cardiac injury indicator (cleaved-Caspase3 and cleaved-PARP1) levels in myocardial tissues. Tubulin was used as an internal reference (*n* = 4 per group). **c**, **e**, **g**, **i**, **k**, **m**, **o** Statistical significance was assessed by two-way ANOVA with Bonferroni multiple comparisons test (*P* values adjusted for 10 comparisons, *P < 0.05; **P < 0.01; ***P < 0.001).

Next, we attempted to inhibit MCT4, a pivotal lactate transporter in cardiomyocytes, to further clarify the above molecular mechanism. α-MHC WT and α-MHC K1897R KI mice underwent control treatment or Ang II infusion and then MCT4 inhibitor VB124 was administered (Fig. 9a). Our results showed that in the α-MHC WT group, α-MHC K1897 lactylation was significantly reduced after Ang II infusion and then increased significantly after subsequent VB124 treatment, while α-MHC K1897 lactylation was almost undetectable in α-MHC K1897R KI mice (Fig. 9b, c). In the α-MHC WT group, the interaction between Titin and α-MHC was significantly reduced after Ang II infusion and increased significantly after subsequent VB124 treatment. However, under Ang II treatment, α-MHC K1897R mutation reversed the increase in α-MHC–Titin interaction by subsequent VB124 treatment compared with the α-MHC WT group (Fig. 9d, e).

We next explored the physiological effects of VB124 in Ang II-induced heart failure of α-MHC WT and α-MHC K1897R KI mice. In the α-MHC WT group, reduction of EF and FS was observed after Ang II treatment, and subsequent VB124 treatment restored the EF and FS decline. However, under Ang II treatment, the α-MHC K1897R mutation partially canceled out the therapeutic effect of VB124 compared with the α-MHC WT group (Fig. 9f, g and Supplementary information, Fig. S8e). In the α-MHC WT group, a significant aggravation of myocardial fibrosis was observed after treatment with Ang II, and subsequent VB124 treatment provided protection from this alteration. However, the α-MHC K1897R mutation partially offset the myocardial fibrosis alleviation caused by the administration of VB124 (Fig. 9h, i). In the α-MHC WT group, a significant aggravation of myocardial hypertrophy was observed after Ang II infusion and merely mild myocardial hypertrophy was observed after being treated with Ang II in α-MHC K1897R KI mice. In addition, subsequent VB124 treatment had no significant effect on myocardial hypertrophy in α-MHC K1897R KI mice (Fig. 9j, k). At the molecular level, increased expressions of α-SMA, Col-1, cleaved-Caspase3 and cleaved-PARP1 were observed after Ang II treatment, and subsequent VB124 treatment resulted in decreased expressions of these biomarkers in the α-MHC WT group. However, under Ang II treatment, the α-MHC K1897R mutation partially abolished the therapeutic effect of VB124 compared with α-MHC WT (Fig. 9l–o).

In conclusion, the MCT4 inhibitor also had a therapeutic effect on heart failure in α-MHC WT mice. However, consistent with the result of NALA, α-MHC K1897R partially abolished the protective effect of MCT4 inhibitor against Ang II-induced heart failure in vivo. Collectively, our results reveal the key role of α-MHC K1897 lactylation in heart failure.

## Discussion

The main finding of this study is that α-MHC K1897 lactylation regulates the interaction between α-MHC and Titin, and that decreasing α-MHC K1897 lactylation aggravates heart failure. We identified lactylation residues by whole-protein lactylation modification-omics analysis and found that α-MHC K1897 lactylation decreased sharply in heart failure mice, which was also confirmed in patients with heart failure by immunohistochemistry. Titin is the largest known protein at present, and it has several highly conserved and continuously repeated domains.^24–26^ A previous study showed that these structural domains of Titin interact with α-MHC to maintain sarcomere stability.^7^ However, what determines the binding and separation of α-MHC and Titin is still unknown. α-MHC K1897R KI mice that fail to undergo lactylation have a reduced interaction between α-MHC and Titin, which disturbs myofibrillar arrangement and impairs cardiac function, and in turn aggravates heart failure. Similarly, Ang II infusion decreases α-MHC K1897 lactylation and weakens the interaction between α-MHC and Titin. This study revealed that lactylation/delactylation of α-MHC K1897 determines the binding/separation of α-MHC and Titin. This is also the first study to examine protein lactylation in hearts under physiological and pathological processes.

**Fig. 10:**
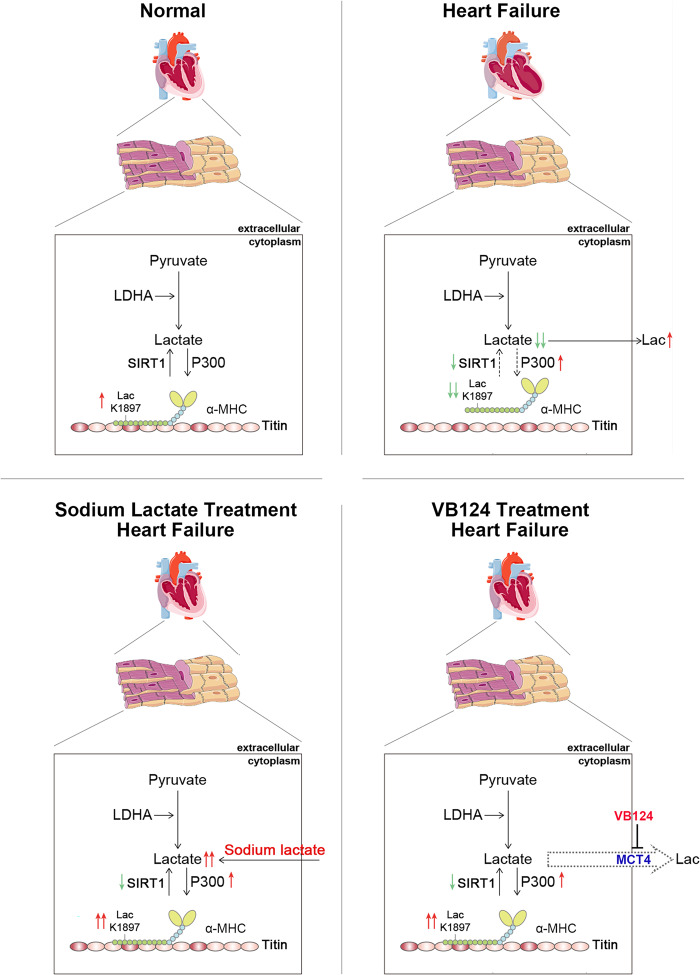
Schematic diagram showing the proposed mechanistic model of α-MHC lactylation. Under the physiological state, α-MHC lactylation reserves the interaction of α-MHC with Titin and maintains sarcomeric structure and function. Upon pathological stress stimulation, a decrease in the lactate concentration in cardiomyocytes leads to reduction of α-MHC lactylation and α-MHC–Titin interaction, thus impairing cardiac structure and function. Upregulation of the lactate concentration by administering sodium lactate or inhibiting MCT4 in cardiomyocytes can promote α-MHC lactylation and α-MHC–Titin interaction, thereby alleviating heart failure.

Our study reveals the core reason for the decrease in α-MHC K1897 lactylation during heart failure. Our results identify four factors, p300, SIRT1, LDHA, and the intracellular lactate concentration, as regulators of α-MHC K1897 lactylation during heart failure. Importantly, we demonstrate that excessive lactate efflux and consumption by cardiomyocytes induce a decrease in lactate concentration, which is the main cause of the reduced α-MHC K1897 lactylation and α-MHC–Titin interaction during myocardial injury (Fig. 10). Consistent with our study, previous study showed that lactate of cardiomyocytes decreased obviously in myocardial injury.^14^ One potential reason is excessive lactate efflux caused by increased MCT4 expression. MCT4 is a member of the MCT family, which is the principal lactate exporter in cells.^27,28^ Although MCT4 expression is low in most cell types including cardiomyocytes, it is markedly upregulated during myocardial injury.^29^ A recent study has indicated that isoproterenol- and Ang II-treated H9c2 cells exhibited an increase in MCT4 protein abundance.^12^ This phenotype may lead to excessive lactate efflux from cardiomyocytes.^12,29^ Another possible reason is that lactate consumption increases during myocardial injury. Lactate consumption almost doubled in failing hearts with left ventricular ejection fraction of less than 40%.^8^ Indeed, we found that NALA and MCT4 inhibitor restore α-MHC K1897 lactylation and interaction between α-MHC and Titin, which significantly improves cardiac function in heart failure. Consistently, previous studies reported that NALA- or MCT4 inhibitor-treated heart produces more ATP and maintains mitochondrial homeostasis during heart failure.^12,17,30^ Our study further supports NALA and MCT4 inhibitor as therapeutic agents for heart failure.

Although p300 is a factor regulating α-MHC K1897 lactylation, it is not a core element in the decrease in α-MHC K1897 lactylation during heart failure. Previous studies have implicated p300 as an acyltransferase that promotes polarization of M1 macrophages through lactylation of H3K18.^10^ Our results showed that p300 was the acyltransferase for α-MHC lactylation under both physiological and pathological conditions. We first hypothesized that p300 was the central factor leading to the reduction of α-MHC K1897 lactylation during heart failure, and p300 activator may restore the decreased α-MHC K1897 lactylation and α-MHC–Titin interaction during heart failure. However, our data did not support this hypothesis (Fig. 3). Thus, p300 may not be the key factor in the decreased α-MHC K1897 lactylation during heart failure. Furthermore, we analyzed the reason that lactylation of α-MHC could not be rescued by activating p300. Previous studies have indicated lactate as the necessary material for lactylation modification.^10^ Our results suggested that the decrease in the intracellular lactate level may be the main reason for the reduction of α-MHC K1897 lactylation during myocardial injury. Therefore, the decrease in α-MHC K1897 lactylation caused by a reduced lactate level may be insufficient to be rescued, even by activating p300. In addition, previous studies have shown that p300 is one of the most complex acyltransferases,^31^ because this enzyme possesses various acyltransferase capabilities,^31–35^ such as acetylation,^31–33^ crotonylation,^34^ and succinylation.^35^

SIRT1 belongs to the sirtuin family that mediates deacetylation of histones and non-histone proteins in an NAD-dependent manner.^36^ Our study confirmed for the first time that SIRT1 performed protein delactylation, and SIRT1 is the delactylase for α-MHC K1897 in both physiological and pathological conditions. We hypothesized that SIRT1 was the major factor in the reduced α-MHC K1897 lactylation during heart failure, which may elevate SIRT1 expression and the binding of α-MHC to SIRT1. Concurrently, SIRT1 inhibition may rescue the reduction in α-MHC K1897 lactylation and α-MHC binding to Titin during heart failure. However, our results cannot support this hypothesis either (Fig. 4). Therefore, SIRT1 may not be the core factor in decreased α-MHC K1897 lactylation during heart failure. We consider that the main reason that α-MHC K1897 lactylation cannot be rescued by inhibiting SIRT1 during heart failure is the lack of lactate in cardiomyocytes. Therefore, α-MHC K1897 lactylation and α-MHC–Titin interaction cannot be significantly rescued during heart failure by inhibiting SIRT1. Furthermore, previous studies have shown that SIRT1 mediates many other types of post-translational modification of proteins, such as de-crotonylation and deacetylation.^37–39^ Therefore, neither p300 nor SIRT1 is the core factor that regulates the α-MHC K1897 lactylation in heart failure.

If LDHA is the main mediator of α-MHC K1897 lactylation, loss of LDHA should be observed during heart failure. However, previous studies have documented that LDHA, as a major player in glucose metabolism, is upregulated during heart dysfunction,^13^ which is consistent with our findings. Although we observed a slight increase in LDHA expression, the lactate concentration in cardiomyocytes was decreased during heart failure. We considered that lactate efflux and consumption of cardiomyocytes may increase even more, resulting in a decrease in the intracellular lactate level, which may explain why lactylation of α-MHC cannot be maintained by increased LDHA expression.

In conclusion, α-MHC K1897 lactylation regulates the interaction between α-MHC and Titin, and the decrease in α-MHC K1897 lactylation predisposes to heart failure. Importantly, the decreased lactate concentration in cardiomyocytes in heart failure may be the main cause of the decrease in α-MHC K1897 lactylation. Furthermore, NALA and an MCT4 inhibitor upregulate α-MHC K1897 lactylation and alleviate heart failure. Our study reveals that cardiac lactate directly modulates the sarcomeric structure and function through α-MHC lactylation, which could serve as a novel therapeutic strategy for heart failure.

## Material and methods

### Immunohistochemistry of human hearts

Formalin-fixed heart tissues were embedded in paraffin and cut into 4-μm sections. The sections were dewaxed in xylene, rehydrated in a series of descending percentages of ethanol, and boiled in Tris-EDTA solution for antigen retrieval. The UltraSensitive^™^ SP IHC Kit (MXB, China) was used for immunostaining. After blocking, the slides were incubated with primary antibody for B-type natriuretic peptide (Affinity Biosciences, China) and α-MHC K1897 lac (PTMBIO, China), p300 (Affinity Biosciences, China) or SIRT1 (Cell Signaling Technology, USA) at 37 °C. After washing, the sections were immersed with biotin-conjugated immunoglobulin G secondary antibody from the immunohistochemistry kit for 20 min, stained by DAB (MXB, China) for 2 min, and then counter-stained in hematoxylin for 2 min. Antibodies and reagents used in this study are listed in Supplementary information, Table S4.

### Animals

α-MHC K1897R KI mice with c.5690A > G mutation (K1897R) of murine *Myh6* and LDHA-cKO mice with myocardium-specific *LDHA* knockout were designed and generated by Shanghai Model Organisms Center, Inc. (Shanghai, China). Briefly, the targeting construct of α-MHC K1897R KI mice was designed to flank exon 38 and 39 with point mutation and a pGK-Neomycine-polyA cassette. The targeting vector was electroporated into C57BL/6J embryonic stem cells (ES), and screening of the G418 resistant colonies was performed according to a routine protocol. The homologous recombined ES cell clones were identified by PCR and confirmed by Southern blot analysis. Tail DNAs were genotyped using primer sets specific to the mutation regions of α-MHC K1897R KI mice. Chimeric mice were bred with Flp mice to obtain F1 generation mice, and their genotypes were identified through PCR and sequencing using following primer pairs:

F1: 5’-TGTGCAGAAAACCCCCAAGTCTG-3’;

R1: 5’-GAGGAACTCACCTTGGCACC-3’.

Specific pathogen-free male mice aged 8–10 weeks were evaluated, and *LDHA* knockout was induced by intraperitoneal injection of 50 mg/kg tamoxifen for 5 consecutive days. In the Ang II infusion model, α-MHC K1897R KI mice, LDHA-cKO mice, and WT mice were intraperitoneally injected with 20 mg/kg of LDHA inhibitor galloflavin; 30 μmol/kg of p300 activator cholera toxin B subunit (CTB) or 30 μmol/kg of p300 inhibitor C646; 20 mg/kg of SIRT1 activator SRT1270 or 1 mg/kg of SIRT1 inhibitor EX527; and 0.2 g/kg NALA or intragastrically administered with 30 mg/kg of MCT4 inhibitor VB124 for 14 consecutive days, before being anesthetized with inhalational isoflurane/oxygen (2%, ~1500 mL/min). An adequate anesthetic depth was confirmed by assessing the lack of the paw withdrawal reflex after anesthesia in the mid-scapular region. An incision was made and an osmotic minipump (Alzet, Model 2002) was implanted subcutaneously following the manufacturer’s instructions. Animals were treated with Alzet (0.5 μL/h) by infusion of Ang II (3 mg/kg/day) for 14 days. And this dosage induces heart failure by causing cardiomyocyte injury and apoptosis.^21^ Ultrasound equipment was used to detect cardiac EF and FS in mice. Cervical dislocation after isoflurane inhalation was used to euthanize the mice. The effects of different treatment factors on the heart were assessed by endpoint blood pressure, left ventricular EF, and left ventricular FS. The animals were handled in accordance with the Animal Welfare Regulations of China Medical University (CMUXN2021730, CMUXN2022120, CMUXN2023007). All animal-involved experiments were approved by the Animal Science Committee of China Medical University. This work was performed in accordance with the Guide for the Care and Use of Laboratory Animals published by the National Institutes of Health (NIH Publication No. 85-23, revised 1985). The reagents used in this study are listed in Supplementary information, Table S4.

### Histopathological staining

Cardiac tissue samples were fixed with 4% formalin for 48 h, embedded in paraffin, and sectioned to 5 μm. This was followed by xylene dewaxing, rehydration with graded ethanol, and staining with H&E (G1120, Solarbio, China), Masson’s trichrome reagent (G1340; Solarbio, China), and WGA (L-1021, Fluorescein F).

### Electron microscopy

Excised hearts were fixed in 2.5% glutaraldehyde, post-fixed in 2% osmium tetroxide, embedded in resin, and sectioned. Ultrastructural studies of cardiac tissue were determined from transmission electron microscopy (H-7650; HITACHI, Japan).

### Protein extraction

Specimens were pulverized in liquid nitrogen and placed in 5 mL tubes, supplemented with four volumes of lysis buffer (8 M urea, 1% protease inhibitor cocktail, 3 μM Trichostatin A, 50 mM nicotinamide), and sonicated three times on ice with a Scientz ultrasonic homogenizer. This was followed by centrifugation-clearing at 12,000 rpm at 4 °C for 10 min. The protein concentration of the resulting supernatant was measured with BCA kit according to the manufacturer’s instructions.

### Trypsin digestion

An equal quantity of total protein in each sample was enzymatically digested, and the volume was adjusted to be consistent across the sample set. TCA was added dropwise to a final concentration of 20%, followed by vortex mixing and precipitation at 4 °C for 2 h. Centrifugation was performed at 4500× *g* for 5 min, and the resulting pellet was washed with pre-cooled acetone three times. After drying, the pellet was resuspended in 200 mM TEAB facilitated by sonication, and trypsin was added to each sample at 1:50 (protease: protein, w/w) for overnight digestion. Dithiothreitol was added at 5 mM followed by incubation at 37 °C for 60 min. Then, 11 mM iodoacetamide was added, followed by incubation for 45 min at room temperature in the dark.

### Enrichment of post-translationally modified peptides

Peptides were resuspended in immunoprecipitation buffer (100 mM NaCl, 1 mM EDTA, 50 mM Tris-HCl, and 0.5% NP-40, pH 8.0) mixed with pre-washed anti-lysine lactylation remnant antibody resin (PTM-1404; Hangzhou Jingjie PTM-Bio) and incubated with gentle shaking overnight at 4 °C. Antibody resin was washed with immunoprecipitation buffer and deionized water. Finally, the enriched peptides were eluted with 0.1% trifluoroacetic acid three times and cleaned with C18 ZipTips.

### Liquid chromatography-tandem mass spectrometry analysis

Tryptic peptides were dissolved in liquid chromatographic mobile phase A and separated on a NanoElute ultra-high performance liquid chromatography system. Mobile phases A and B were 0.1% formic acid and 2% acetonitrile in water and 0.1% formic acid in acetonitrile, respectively. The peptides were eluted using the gradient set as 0–72 min, 7%–24% B; 72–84 min, 24%–32% B; 84–87 min, 32%–80% B; and 87–90 min, 80% B, with a constant flow rate of 450 nL/min. The peptides were separated on a capillary column (25 cm length, 100 µm inner ID, 1.9 µm particle size) before injecting into a capillary ion source for ionization and timsTOF Pro mass spectrometer (ion source voltage, 1.6 kV; scanning range, 100–1700 Da). The parallel accumulation serial fragmentation (PASEF) mode was enabled for data acquisition. Precursors with charge states of 0–5 were selected for fragmentation, and 10 PASEF tandem mass spectrometry scans were acquired per cycle. The dynamic exclusion time of tandem mass spectrometry scanning was 30 s to prevent multiple scanning of the same parent ions.

For the lactylation analysis, tryptic peptides were dissolved in liquid chromatographic mobile phase A and separated on a NanoElute ultra-high performance liquid chromatography system. Mobile phases A and B were 0.1% formic acid and 2% acetonitrile in water and 0.1% formic acid in acetonitrile, respectively. The peptides were eluted using the gradient set as 0–44 min, 6%–22% B; 44–56 min, 22%–30% B; 56–58 min, 30%–80% B; and 58–60 min, 80% B, with a constant flow rate of 450 nL/min. The peptides were separated on a capillary column (100 µm inner ID, 1.9 µm particle size) before injecting into a capillary ion source for ionization and timsTOF Pro mass spectrometer (ion source voltage, 1.6 kV; scanning range, 100–1700 Da). The PASEF mode was enabled for data acquisition. Precursors with charge states of 0–5 were selected for fragmentation, and 10 PASEF tandem mass spectrometry scans were acquired per cycle. The dynamic exclusion time of tandem mass spectrometry scanning was 24 s to prevent multiple scanning of the same parent ions.

### Database search

Raw mass spectrometry data were searched against a Swissprot protein sequence database (Mus_musculus_10090_SP. fasta) by Maxquant (v1.6.6.0) with reverse decoy entries and common contamination proteins. A maximum of two missing cleavages was allowed for trypsin/P digestion, and at least seven amino acids were required for each peptide. The mass error tolerance for the precursor ion was 10 ppm, and for the product ion was 20 ppm. Cysteine alkylation (carbamidomethyl (C)) was considered a fixed modification. Variable modifications were methionine oxidation and N-terminal acetylation. Lysine lactylation on lysine was also set as variable modifications for the corresponding modification enrichment analysis. The false discovery rates for protein and PSM identification were both 1%.

### Cell culture

H9c2, HL-1, and HEK293T cells were obtained from the American Type Culture Collection and cultured in high-glucose Dulbecco’s Modified Eagle Medium with 10% fetal bovine serum, penicillin (100 μg/mL), and streptomycin (100 μg/mL) in a humidified atmosphere of 5% carbon dioxide at 37 °C.

### Plasmid construction and transfection

All the plasmids and siRNA used in this study are listed in Supplementary information, Table S3 and the reagents used in this study are listed in Supplementary information, Table S4. The plasmids were confirmed by sequencing. The manufacturer’s instructions were followed for plasmid transfection into H9c2, HL-1, and HEK293T cells using Lipofectamine 3000 (Invitrogen, USA), HiGene (Applygen, China), and jetPRIME (Polyplus, France). The cells were collected 48–72 h after transfection. LDHA inhibitor (Galloflavin; Cat#HY-W040118; 10^−5^ M; 24 h), p300 inhibitor (C646; Cat#HY-13823; 10^−5^ M; 24 h), p300 activator (CTB; Cat#HY-134964; 10^−4^ M; 24 h) and NALA (Cat#HY-B2227B; 300 mM; 24 h) were obtained from MedChemExpress (USA). SIRT1 inhibitor (EX527; Cat#A4181; 10^−5^ M; 24 h) and SIRT1 activator (SRT1720; Cat#A4180; 5 μM; 24 h) were obtained from APExBio (USA). Angiotensin II (Cat#HY-13948; 10^−4^ M) was from APExBio (USA).

### WB analysis and immunoprecipitation

Mouse heart tissues and cells were lysed with lysis buffer (137 mM NaCl, 10 mM NaF, 50 mM Tris-HCl, pH 7.6, 1 mM EDTA, 0.1 mM sodium orthovanadate, 10% glycerol, 1% NP-40, 1 mM protease inhibitor). For WB analysis, the protein samples were quantified, and the total quality and volume of each protein sample were adjusted according to the target protein expression. WB for detecting LDHA, α-SMA, α-MHC and α-MHC K1897-Lactyl Lysine in mouse myocardial tissues were performed with 10 µg of protein extracts, and Col-1, cleaved-Caspase3, cleaved-PARP1 in mouse myocardial tissues were performed with 40 µg of protein extracts. WB for p300 and SIRT1 in H9c2 cells and mouse myocardial tissues were performed with 40 µg of protein extracts. For Co-IP and lactylation immunoprecipitation, 40 µg protein lysate was used for input and 10 µg was used for input of mouse myocardial tissues, 30 μL of pre-washed magnetic beads was added to 1 mg protein lysate and incubated with rotation for 20 min at room temperature. Subsequently, the beads were separated from the lysates by magnetic separation, and the pre-cleared lysate was transferred into clean tubes. Then, the 1 mg of protein lysate was incubated with primary antibody (anti-Pan Kla, 4 µg, PTMBIO; anti-Titin, 4 µg, Santa Cruz; anti-α-MHC, 4 µg, Santa Cruz; anti-MHC K1897 lactyl-lysine, 4 µg, PTMBIO; anti-p300, 1 µg, Cell Signaling Technology; anti-SIRT1, 1 µg, Proteintech) conjugated with 30 μL of Protein A/G magnetic beads (Cat#B23202, Biotool, USA), 20 μL of anti-Myc magnetic beads (Cat#B26302, Biotool, USA), or 20 μL of Anti-Flag Affinity Gel (Cat#B23102, Biotool, USA) for 12 h at 4 °C. The complexes were then washed with cold lysis buffer and eluted with sodium dodecyl sulfate (SDS) loading buffer. SDS-polyacrylamide gel electrophoresis was used to separate the complexes followed by electro-transfer onto polyvinylidene fluoride (PVDF) membranes. The PVDF membranes were incubated with 5% bovine serum albumin for 1 h at room temperature and then successively incubated with primary (4 °C, overnight) and secondary (room temperature, 1 h) antibodies. Protein expression was quantitated using ImageJ v1.46 (National Institutes of Health, USA). Protein expression was normalized by Tubulin. For detecting protein expressing in WB and immunoprecipitation, antibodies were all used at 1:1000 dilution. Antibodies and reagents used in this study are listed in Supplementary Information, Table S4.

### Lactate measurements

The concentrations of mouse serum lactate, mouse heart lactate, H9c2 cell lactate (intracellular), and cell medium lactate were measured using the CheKine^™^ Lactate Assay Kit (KTB1100, Abbkine, China).

### Statistical analyses

The data are presented as mean ± SD. The *F*-test (two groups) or the Brown–Forsythe test (three or more groups) was used to assess the homogeneity of variance. The Shapiro–Wilk test was used to assess the normality of the data. Student’s *t*-test and Welch’s *t-*test were used for equal variance and unequal variance, respectively (two groups). Two-way analysis of variance with the Bonferroni multiple-comparisons test was used when two conditions were considered between the groups. The *p* values were adjusted for multiple comparisons where appropriate. GraphPad Prism 8.0 software (GraphPad, USA) and SPSS 22.0 (SPSS, USA) were used for all statistical analyses, with a *P* value less than 0.05 indicating statistical significance.

### Ethical approval

The study included five male patients and five age- and gender-matched controls. Failing heart samples were obtained from patients with end-stage heart failure (average EF was 20 ± 5%) at the time of cardiac transplantation (General Hospital of Northern Theater Command). Non-failing hearts were obtained from donors who had normal cardiac contractile function by echocardiography or anatomical analysis of remains and died from accidents (Department of Forensic Medicine, China Medical University; Center of Organ Transplantation, The First Hospital of China Medical University). The procurement of the heart tissues conforms to the principles outlined in the Declaration of Helsinki and was approved by the Institutional Ethics Committee of The First Hospital of China Medical University (protocol number AF-SOP-07-1.1-01; [2022]504).

## Supplementary information

Supplementary information, Fig. S1

Supplementary information, Fig. S2

Supplementary information, Fig. S3

Supplementary information, Fig. S4

Supplementary information, Fig. S5

Supplementary information, Fig. S6

Supplementary information, Fig. S7

Supplementary information, Fig. S8

Supplementary information, Table S1

Supplementary information, Table S2

Supplementary information, Table S3

Supplementary information, Table S4
