# Supplementary material for: α-myosin heavy chain lactylation maintains sarcomeric structure and function and alleviates the development of heart failure
Source: Cell Res. 2023 Jul 13;33(9):679–98. doi: 10.1038/s41422-023-00844-w (PMC10474270; doi:10.1038/s41422-023-00844-w)
Supplement: Supplementary file 1 — Supplementary information, Fig. S1 [file 41422_2023_844_MOESM1_ESM.pdf]

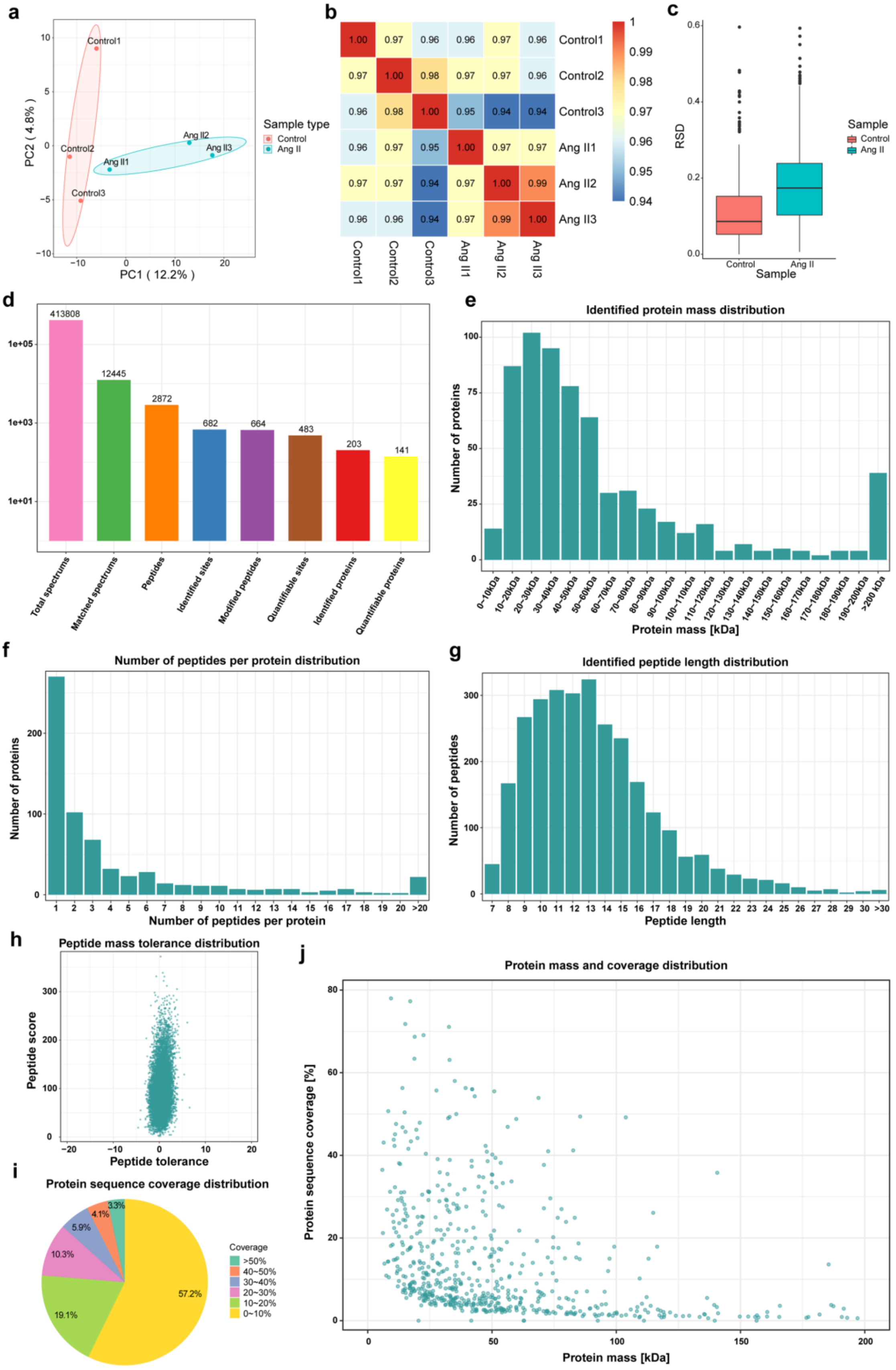
a
PC1 (12.2%)
PC2 (4.8%)
Control1
Control2
Control3
Ang II1
Ang II2
Ang II3
Sample type
Control
Ang II
b
Control1
Control2
Control3
Ang II1
Ang II2
Ang II3
c
RSD
Sample
Control
Ang II
d
413808
12445
2872
682
664
483
203
141
Total spectrums
Matched spectrums
Peptides
Identified sites
Modified peptides
Quantifiable sites
Identified proteins
Quantifiable proteins
e
Identified protein mass distribution
Number of proteins
Protein mass [kDa]
f
Number of peptides per protein distribution
Number of proteins
Number of peptides per protein
g
Identified peptide length distribution
Number of peptides
Peptide length
h
Peptide mass tolerance distribution
Peptide score
Peptide tolerance
i
Protein sequence coverage distribution
Coverage
>50%
40~50%
30~40%
20~30%
10~20%
0~10%
57.2%
19.1%
10.3%
5.9%
4.1%
3.3%
j
Protein mass and coverage distribution
Protein sequence coverage [%]
Protein mass [kDa]

Supplementary information, Fig. S1 Quality control of lactylation modification in normal and Ang II-induced heart failure mice.

a Principal component analysis (PCA) showing the first two principal components of protein intensities, with specimens linked by centroids based on sample type. **b** Pearson's correlation analysis; each value represents the correlation coefficient between the two samples. **c** A boxplot based on relative standard deviation (RSD), with each point representing an RSD value. **d** Histogram of mass spectrometry results. **e** Protein molecular weight statistics; each bar height represents the number of proteins. **f** Peptide number statistics after enzymatic hydrolysis; each bar height represents the number of proteins. **g** Peptide length statistics; each bar height represents the number of peptides. **h** Volcano plot showing peptide mass tolerance distribution. **i** Pie chart showing protein sequence coverage distribution. **j** Protein mass and coverage distribution.
