# Supplementary figures and images for: α-myosin heavy chain lactylation maintains sarcomeric structure and function and alleviates the development of heart failure
Source: Cell Res. 2023 Jul 13;33(9):679–98. doi: 10.1038/s41422-023-00844-w (PMC10474270; doi:10.1038/s41422-023-00844-w)

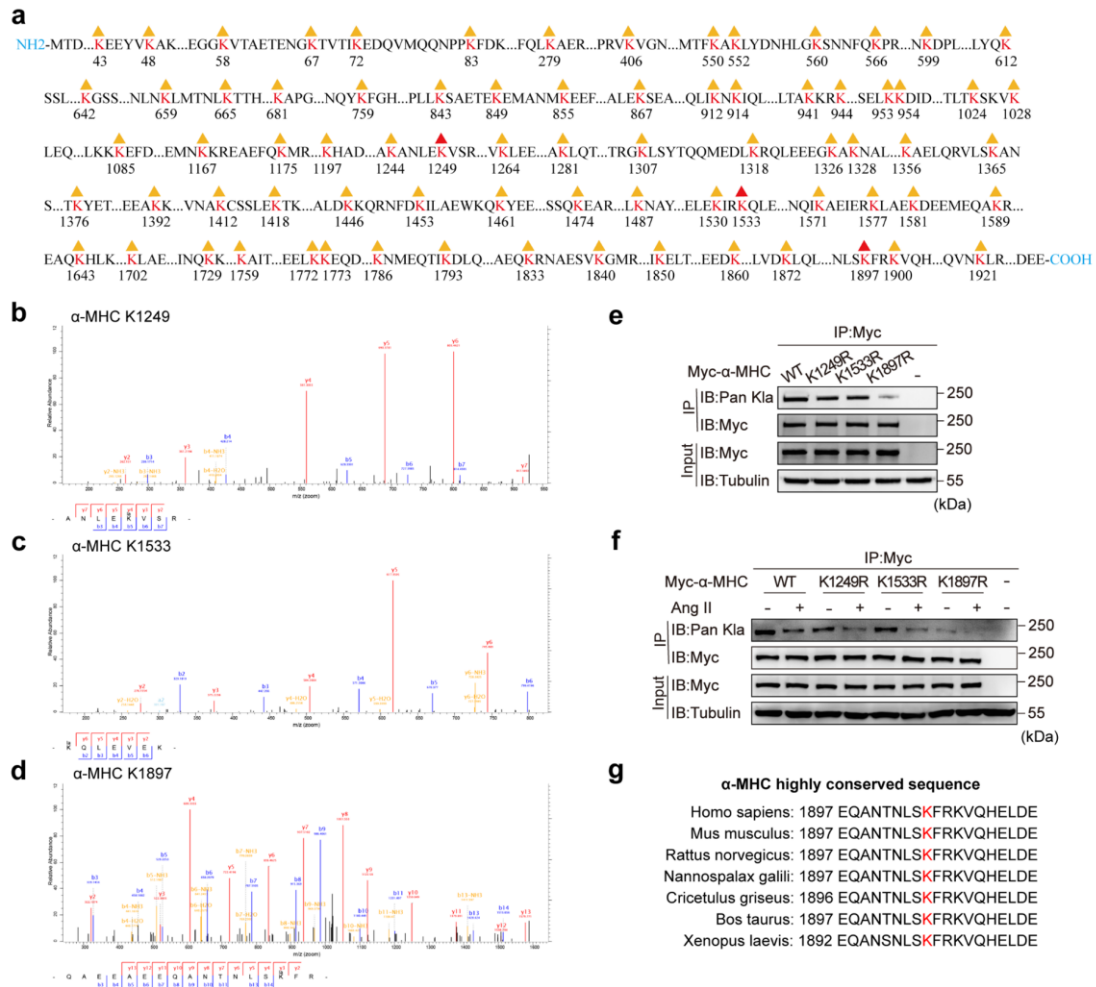

Supplement: Supplementary file 2 — Supplementary information, Fig. S2 [file 41422_2023_844_MOESM2_ESM.pdf]
