# Supplementary material for: α-myosin heavy chain lactylation maintains sarcomeric structure and function and alleviates the development of heart failure
Source: Cell Res. 2023 Jul 13;33(9):679–98. doi: 10.1038/s41422-023-00844-w (PMC10474270; doi:10.1038/s41422-023-00844-w)
Supplement: Supplementary file 3 — Supplementary information, Fig. S3 [file 41422_2023_844_MOESM3_ESM.pdf]

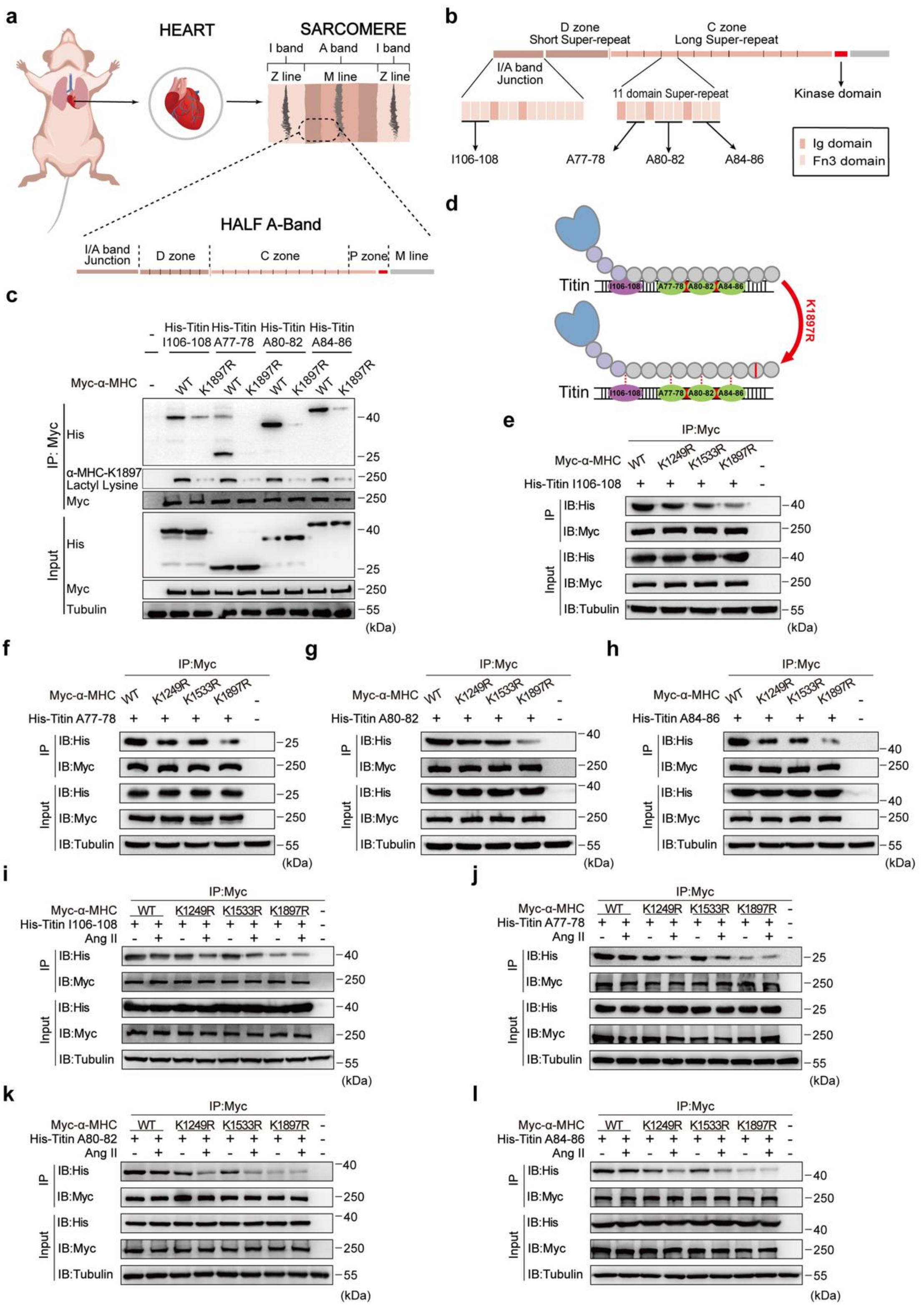

Supplementary information, Fig. S3 α-MHC K1897R mutation reduces the interaction between α-MHC and Titin.

a Schematic of sarcomere in mice myocardial tissue. The intercepts showing the structure of the half A-band in sarcomere. **b** Overview of Titin structure. Ig domain was painted in dark red, fn3

domain was painted in light red. Underneath, the location of the fn3-fragments (I106-108, A77-78, A80-82 & A84-86) in Titin was indicated. **c** IP showed diminished interaction between α-MHC and Titin resulting from the mutation of α-MHC K1897R in HEK293T cells. Anti-Myc magnetic beads were added in per immunoprecipitated samples, followed by detection of His-Titin fragments (I106-108, A77-78, A80-82 & A84-86) and α-MHC K1897 Lactyl Lysine. **d** Schematic showed diminished interaction between α-MHC and Titin resulting from mutation of the α-MHC K1897 site. **e-l** Interaction between α-MHC and Titin under physiological conditions and Ang II treatment conditions were determined by IP analysis. (**e-h**) HEK293T cells were transfected with plasmids expressing Myc-α-MHC WT, Myc-α-MHC K1249R, Myc-α-MHC K1533R, Myc-α-MHC K1897R and transfected with plasmids expressing fn3-fragments His-Titin I106-108 (**e**), His-Titin A77-78 (**f**), His-Titin A80-82 (**g**), His-Titin A84-86 (**h**). Equal amounts of lysates were prepared for IP with Anti-Myc magnetic beads, followed by detection of His. (**i-l**) HEK293T cells were transfected with plasmids expressing Myc-α-MHC WT, Myc-α-MHC K1249R, Myc-α-MHC K1533R, Myc-α-MHC K1897R and transfected with plasmids expressing fn3-fragments His-Titin I106-108 (**i**), His-Titin A77-78 (**j**), His-Titin A80-82 (**k**), His-Titin A84-86 (**l**) without or with Ang II treatment. Equal amounts of lysates were prepared for IP with anti-Myc magnetic beads, followed by detection of His.
