# Supplementary material for: α-myosin heavy chain lactylation maintains sarcomeric structure and function and alleviates the development of heart failure
Source: Cell Res. 2023 Jul 13;33(9):679–98. doi: 10.1038/s41422-023-00844-w (PMC10474270; doi:10.1038/s41422-023-00844-w)
Supplement: Supplementary file 4 — Supplementary information, Fig. S4 [file 41422_2023_844_MOESM4_ESM.pdf]

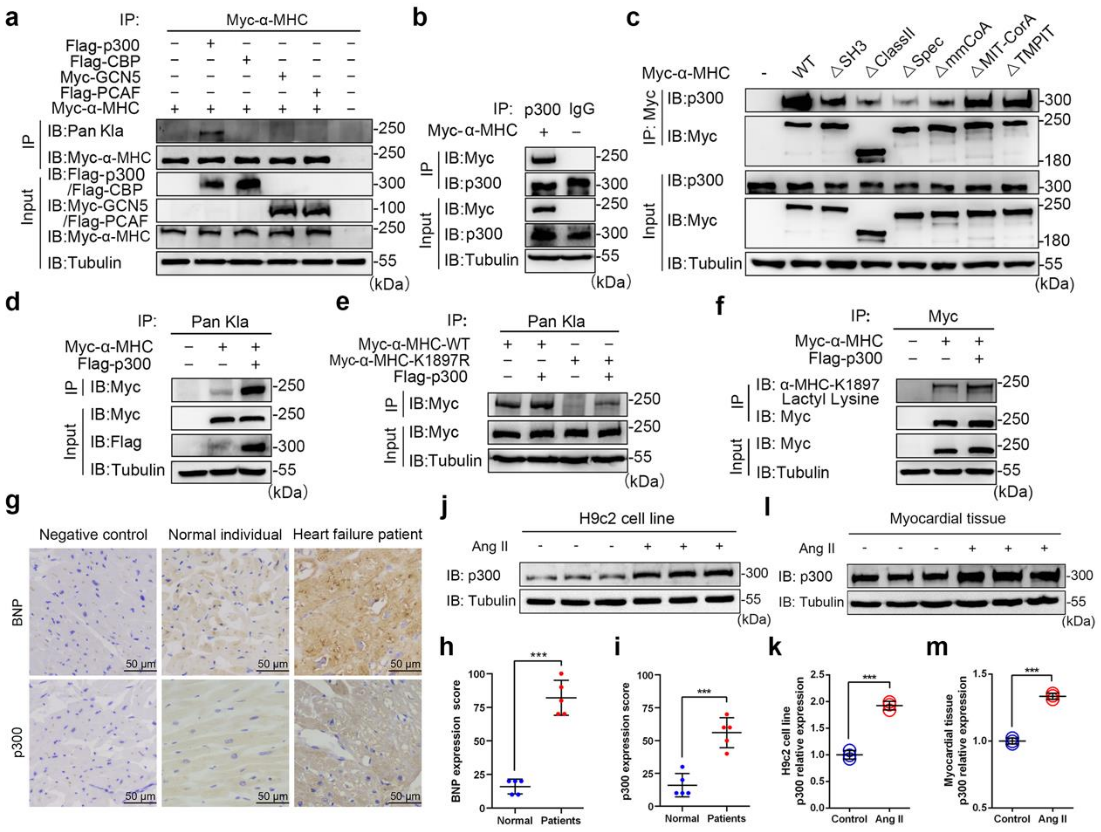

Supplementary information, Fig. S4 p300 is the acyltransferase of α-MHC K1897 lactylation.

a Lactylation of α-MHC was determined by IP analysis. HEK293T cells were transfected with plasmids expressing Myc-α-MHC, accompanied by overexpression of Flag-p300, Flag-CBP, Myc-GCN5 or Flag-PCAF. Equal amounts of lysates were prepared for IP with anti-Myc magnetic beads, followed by detection of Pan Kla. **b** HEK293T cells were transfected with Myc-α-MHC plasmid. Control IgG or anti-p300 antibody was used for IP followed by detection of Myc. **c** α-MHC domains binding to p300 were determined by IP analysis. HEK293T cells were transfected with plasmids expressing Myc-α-MHC WT, Myc-α-MHC-ΔSH3, Myc-α-MHC-ΔClassII, Myc-α-MHC-ΔSpec, Myc-α-MHC-ΔmmCoA, Myc-α-MHC-ΔMIT-CorA and Myc-α-MHC-ΔTMPIT (truncations). Equal amounts of lysates were prepared for IP with anti-Myc magnetic beads, followed by detection of p300. **d** IP analysis of lactylation of α-MHC. HEK293T cells were transfected with the indicated plasmids (Flag-p300 and Myc-α-MHC). Equal amounts of lysates were prepared for IP with anti-Pan Kla antibody followed by detection of Myc. **e** IP analysis of lactylation of α-MHC. HEK293T cells were transfected with the indicated plasmids (Myc-α-MHC WT, Myc-α-MHC K1897R and Flag-p300). Equal amounts of lysates were prepared for IP with anti-Pan Kla antibody, followed by detection of Myc. **f** IP analysis of α-MHC K1897 lactylation. HEK293T cells were transfected with

indicated plasmids (Myc-α-MHC and Flag-p300) using anti-Myc magnetic beads, followed by detection of α-MHC K1897 Lactyl Lysine. **g** Representative immunohistochemical (IHC) staining of BNP (top, using anti-BNP antibody) and p300 (bottom, using anti-p300 antibody) proteins in the heart tissues from normal controls and heart failure patients. Scale bars, 50μm. Negative controls were performed with normal rabbit IgG. **h**, **i** Quantification of the relative BNP and p300 expression score. (n=5 per group). **j** Western blot analysis to assess p300 expression levels in H9c2 cells with or without Ang II treatment. **k** Quantification of relative p300 expression in H9c2 cells. **l** Western blot analysis to assess p300 expression levels in myocardial tissues from mice after with or without Ang II treatment. **m** Quantification of relative p300 expression in myocardial tissues. (**a, c, f**) Anti-Myc magnetic beads were added in per immunoprecipitated sample. (**b**) Anti-p300 antibody or control IgG were added. (**d, e**) Anti-Pan Kla antibody was added. (**h, i, k, m**) Data are expressed as means ± SD. Statistical significance was assessed by Student's *t-test* (*** $P < 0.001$).
