# Supplementary material for: α-myosin heavy chain lactylation maintains sarcomeric structure and function and alleviates the development of heart failure
Source: Cell Res. 2023 Jul 13;33(9):679–98. doi: 10.1038/s41422-023-00844-w (PMC10474270; doi:10.1038/s41422-023-00844-w)
Supplement: Supplementary file 5 — Supplementary information, Fig. S5 [file 41422_2023_844_MOESM5_ESM.pdf]

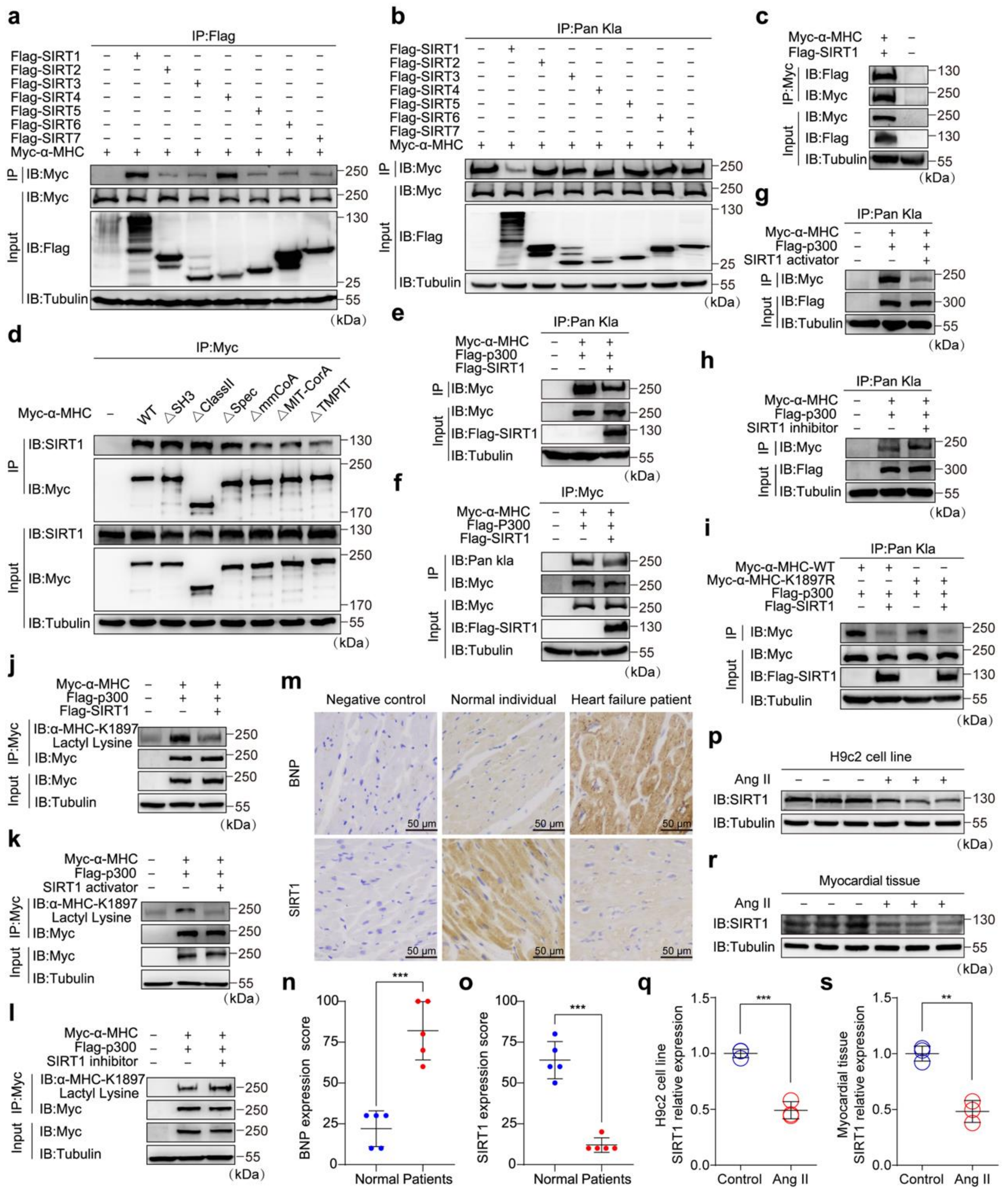

**Supplementary information, Fig. S5 SIRT1 is the delactylase of α-MHC K1897.**

**a, b** Lactylation of α-MHC was determined by IP analysis. HEK293T cells were transfected with plasmids expressing Myc-α-MHC, accompanied by overexpression of Flag-SIRT1, Flag-SIRT2, Flag-SIRT3, Flag-SIRT4, Flag-SIRT5, Flag-SIRT6, or Flag-SIRT7. (**a**) Equal amounts of lysates were prepared for IP with anti-Flag Affinity Gel, followed by detection of anti-Myc antibody. (**b**) equal amounts of lysates were prepared for IP with anti-Pan Kla antibody, followed by detection of Myc. **c** IP analysis of HEK293T cells transfected with plasmids expressing Myc-α-MHC and Flag-SIRT1. Anti-Myc magnetic beads was used for IP followed by detection of Flag. **d** α-MHC domains

binding to SIRT1 were determined by IP analysis. HEK293T cells were transfected with plasmids expressing Myc-α-MHC WT, Myc-α-MHC-ΔSH3, Myc-α-MHC-ΔClassII, Myc-α-MHC-ΔSpec, Myc-α-MHC-ΔmmCoA, Myc-α-MHC-ΔMIT-CorA and Myc-α-MHC-ΔTMPIT (truncations). Equal amounts of lysates were prepared for IP with anti-Myc magnetic beads, followed by detection of SIRT1. **e, f** IP analysis of lactylation of α-MHC. HEK293T cells were transfected with the indicated plasmids (Flag-SIRT1, Flag-p300, and Myc-α-MHC). (**e**) Equal amounts of lysates were prepared for IP with anti-Pan Kla antibody, followed by detection of Myc; (**f**) equal amounts of lysates were prepared for IP with anti-Myc magnetic beads, followed by detection of Pan Kla. **g, h** IP analysis of lactylation of α-MHC induced with SIRT1 activator or inhibitor. HEK293T cells were transfected with the indicated plasmids (Flag-p300, and Myc-α-MHC), with or without SIRT1 activator (**g**) and SIRT1 inhibitor (**h**). Equal amounts of lysates were prepared for IP with anti-Pan Kla antibody, followed by detection of Myc. **i** IP analysis of lactylation of α-MHC K1897. HEK293T cells were transfected with the indicated plasmids (Myc-α-MHC WT, Myc-α-MHC K1897R, Flag-p300 and Flag-SIRT1). Equal amounts of lysates were prepared for IP with anti-Pan Kla antibody, followed by detection of Myc. **j** IP analysis of lactylation of α-MHC. HEK293T cells were transfected with indicated plasmids (Myc-α-MHC, Flag-p300 and Flag-SIRT1) using anti-Myc magnetic beads, followed by detection of α-MHC K1897 Lactyl Lysine. **k, l** IP analysis of lactylation of α-MHC K1897 with SIRT1 activator and inhibitor. HEK293T cells were transfected with the indicated plasmids (Myc-α-MHC and Flag-p300), with or without SIRT1 activator (**k**) and SIRT1 inhibitor (**l**). Equal amounts of lysates were prepared for IP with anti-Myc magnetic beads, followed by detection of α-MHC K1897 Lactyl Lysine. **m** Representative immunohistochemical (IHC) staining of BNP (top, using anti-BNP antibody) and SIRT1 (bottom, using anti-SIRT1 antibody) proteins in the heart tissues from normal controls and heart failure patients. Scale bars, 50μm. Negative controls were performed with normal rabbit IgG. **n**, **o** Quantification of the relative BNP and SIRT1 expression score. (n=5 per group). **p** Western blot analysis to assess SIRT1 expression levels in H9c2 cells with or without Ang II treatment. **q** Quantification of relative SIRT1 expression in H9c2 cells. **r** Western blot analysis to assess SIRT1 expression levels in myocardial tissues from mice after NaCl or Ang II treatment. **s** Quantification of relative SIRT1 expression in myocardial tissues. (**a**) Anti-Flag Affinity Gel was added in per immunoprecipitated sample. (**b, e, g-i**) Anti-Pan Kla antibody was added. (**c, d, f, j-l**) Anti-Myc magnetic beads were added in per

immunoprecipitated sample. (**n, o, q, s**) Data are expressed as means ± SD. Statistical significance was assessed by Student's *t-test* (** $P < 0.01$; *** $P < 0.001$).
