# Supplementary material for: α-myosin heavy chain lactylation maintains sarcomeric structure and function and alleviates the development of heart failure
Source: Cell Res. 2023 Jul 13;33(9):679–98. doi: 10.1038/s41422-023-00844-w (PMC10474270; doi:10.1038/s41422-023-00844-w)
Supplement: Supplementary file 6 — Supplementary information, Fig. S6 [file 41422_2023_844_MOESM6_ESM.pdf]

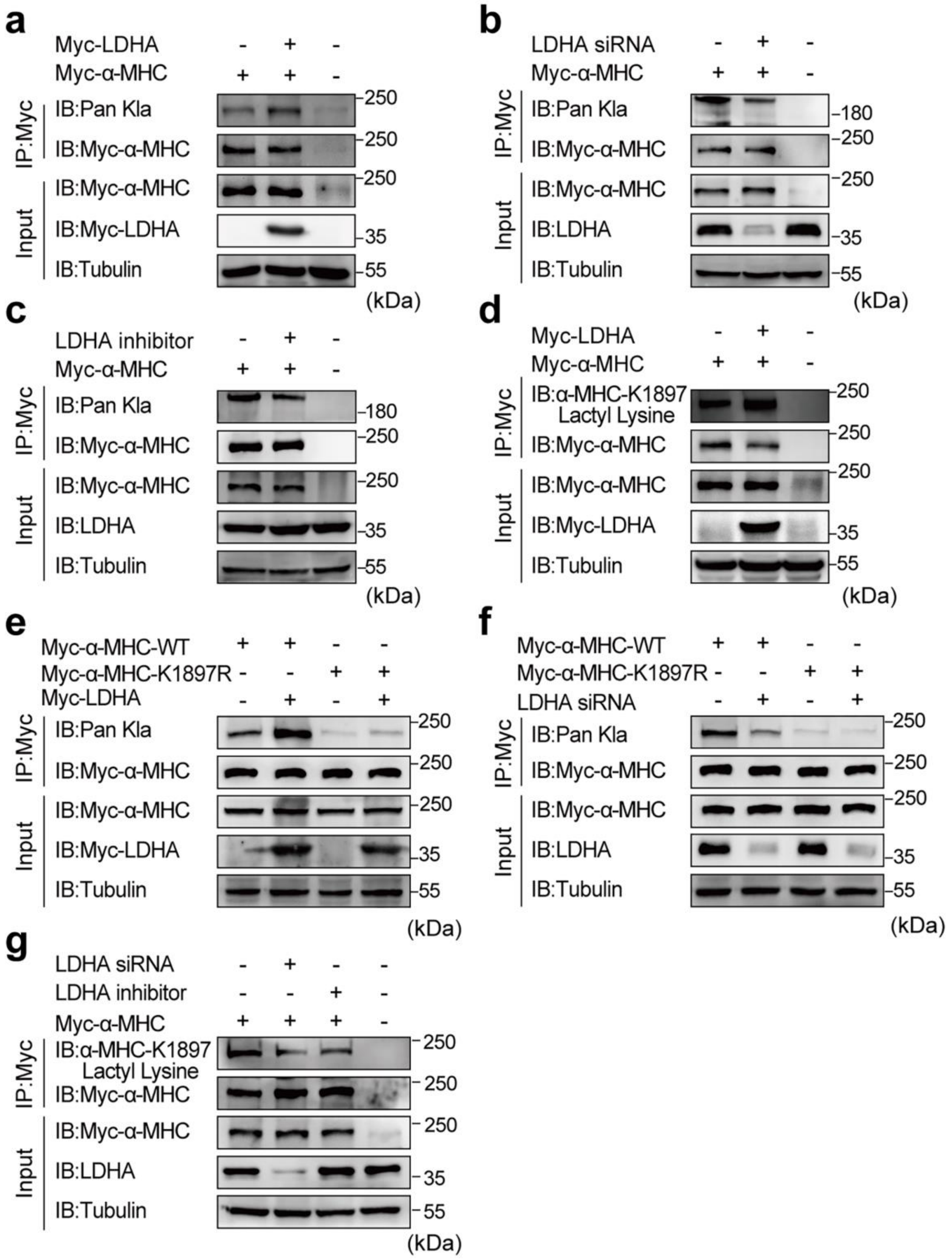

Supplementary information, Fig. S6 LDHA increases lactylation of α-MHC K1897.

a Lactylation of α-MHC was determined by IP analysis. HEK293T cells were transfected with plasmids expressing Myc-α-MHC and Myc-LDHA. Equal amounts of lysates were prepared for IP with anti-Myc magnetic beads, followed by detection of Pan Kla. **b** HEK293T cells were transfected with plasmids expressing Myc-α-MHC and transfected with negative control or LDHA siRNAs. Equal amounts of lysates were prepared for IP with anti-Myc magnetic beads, followed by detection

of Pan Kla. **c** IP analysis of lactylation of α-MHC with LDHA inhibitor. HEK293T cells were transfected with plasmids expressing Myc-α-MHC and treated with or without LDHA inhibitor. Equal amounts of lysates were prepared for IP with anti-Myc magnetic beads, followed by detection of Pan Kla. **d** IP analysis of lactylation of α-MHC K1897. HEK293T cells were transfected with plasmids expressing Myc-α-MHC and Myc-LDHA. Equal amounts of lysates were prepared for IP with anti-Myc magnetic beads, followed by detection of α-MHC K1897 Lactyl Lysine. **e** HEK293T cells were transfected with plasmids expressing Myc-α-MHC WT, Myc-α-MHC K1897R and Myc-LDHA. Equal amounts of lysates were prepared for IP with anti-Myc magnetic beads, followed by detection of Pan Kla. **f** HEK293T cells were transfected with plasmids expressing Myc-α-MHC WT, Myc-αMHC K1897R, and transfected with negative control or LDHA siRNAs. Equal amounts of lysates were prepared for IP with anti-Myc magnetic beads, followed by detection of Pan Kla. **g** HEK293T cells were transfected with plasmids expressing Myc-α-MHC and treated with LDHA inhibitor or LDHA siRNAs. Equal amounts of lysates were prepared for IP with anti-Myc magnetic beads, followed by detection of α-MHC K1897 Lactyl Lysine. (**a-g**) Anti-Myc magnetic beads were added in per immunoprecipitated sample.
