# Supplementary material for: α-myosin heavy chain lactylation maintains sarcomeric structure and function and alleviates the development of heart failure
Source: Cell Res. 2023 Jul 13;33(9):679–98. doi: 10.1038/s41422-023-00844-w (PMC10474270; doi:10.1038/s41422-023-00844-w)
Supplement: Supplementary file 7 — Supplementary information, Fig. S7 [file 41422_2023_844_MOESM7_ESM.pdf]

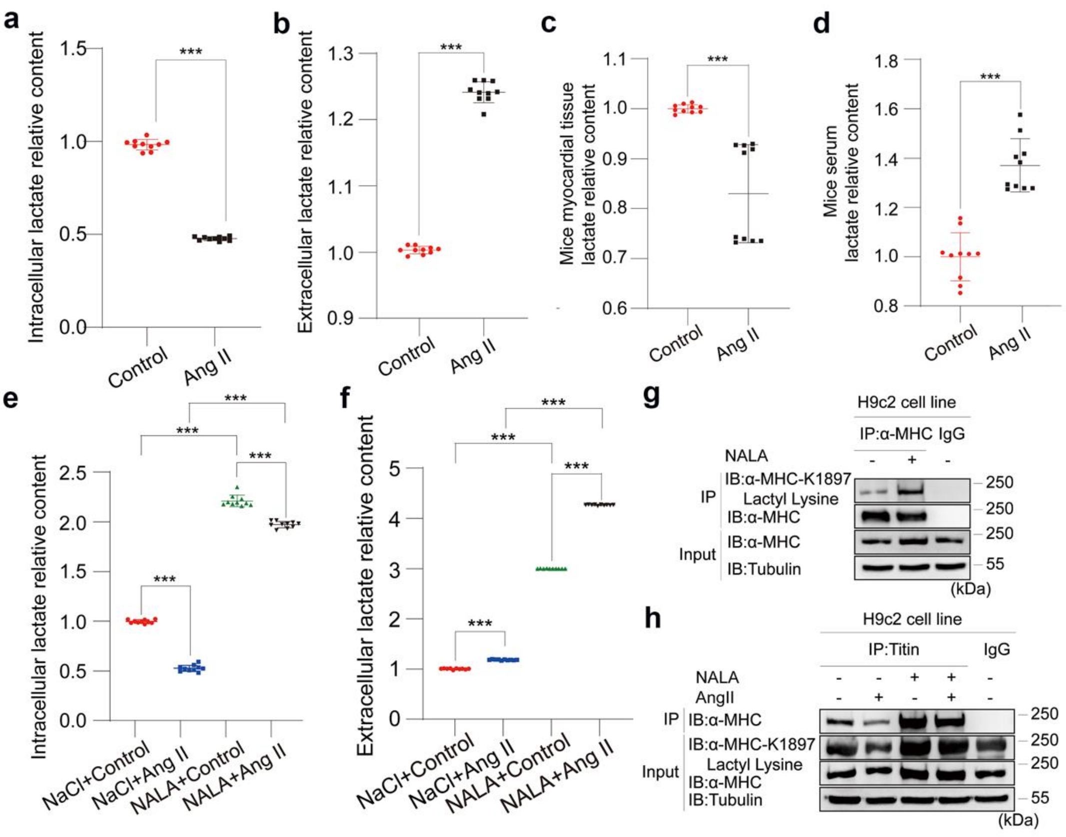

Supplementary information, Fig. S7 NALA increases the lactate level and lactylation of α-MHC K1897, further increasing the α-MHC-Titin interaction.

a, b Relative content of intracellular (**a**) and extracellular (**b**) lactate in H9c2 cells after 24h of NaCl or Ang II treatment (n=10 per group). **c, d** Relative content of lactate in myocardial tissues (**c**) and serum (**d**) of mice after NaCl or Ang II treatment (n=10 per group). **e, f** Relative content of intracellular (**e**) and extracellular (**f**) lactate in NaCl or Ang II-stimulated H9c2 cells with or without NALA treatment as indicated (n=10 per group). **g** H9c2 cells with or without NALA treatment were lysed and immunoprecipitated using control IgG or anti-α-MHC antibody, followed by detection of α-MHC K1897 Lactyl Lysine. **h** NaCl or Ang II-stimulated H9c2 cells with or without NALA treatment were lysed and immunoprecipitated using control IgG or anti-Titin antibody, followed by detection of α-MHC. (**g, h**) Anti-α-MHC antibody, anti-Titin antibody or control IgG were added. (**a-d**) Data are expressed as means ± SD. Statistical significance was assessed by Student's *t-test* (*** $P < 0.001$). (**e, f**) Data are expressed as means ± SD. Statistical significance was assessed by 2-way ANOVA with Bonferroni multiple comparisons test (*P* values adjusted for 6 comparisons, *** $P < 0.001$).
