# Supplementary material for: α-myosin heavy chain lactylation maintains sarcomeric structure and function and alleviates the development of heart failure
Source: Cell Res. 2023 Jul 13;33(9):679–98. doi: 10.1038/s41422-023-00844-w (PMC10474270; doi:10.1038/s41422-023-00844-w)
Supplement: Supplementary file 8 — Supplementary information, Fig. S8 [file 41422_2023_844_MOESM8_ESM.pdf]

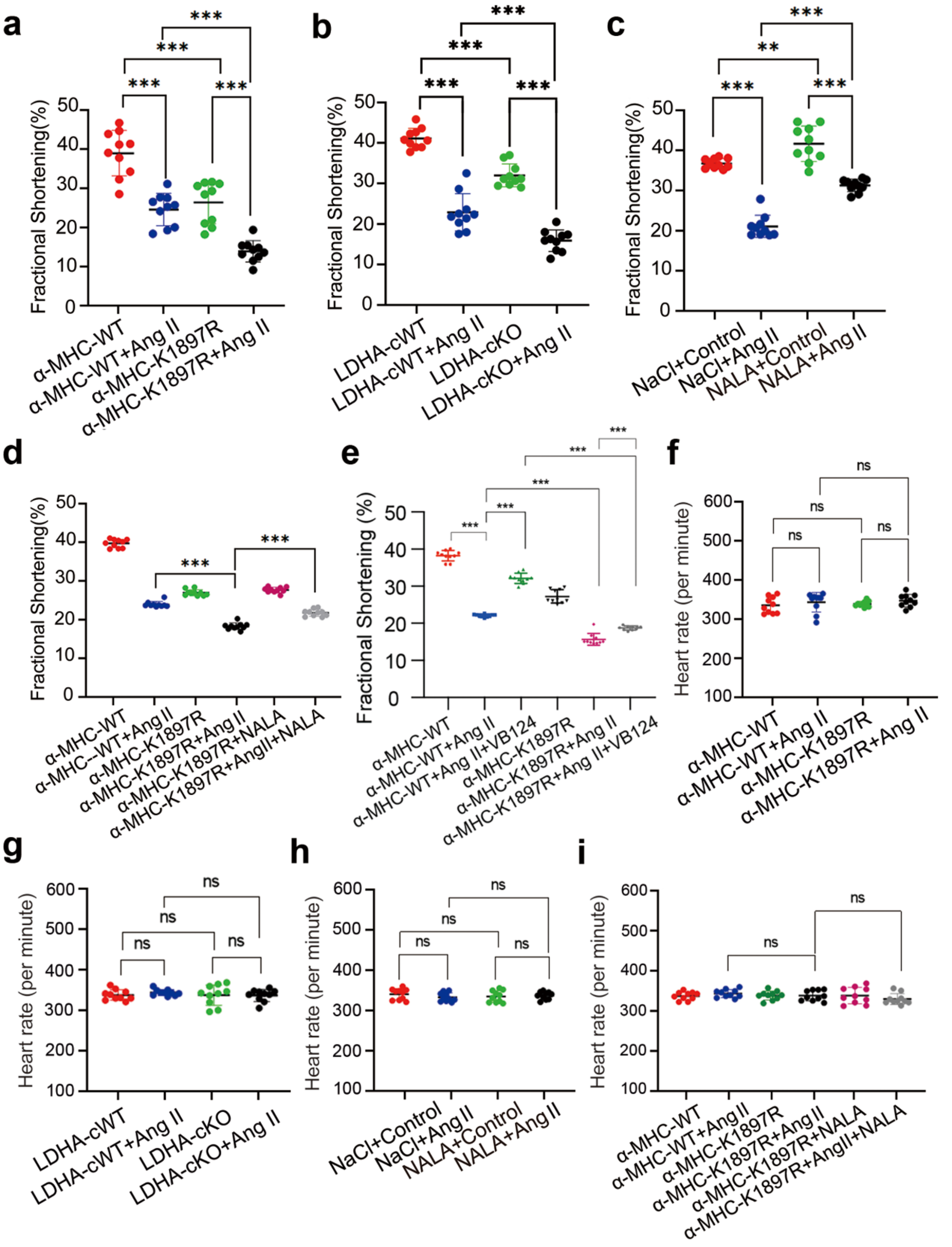

Supplementary information, Fig. S8 Fractional shortening and heart rate of mice.

a, f Cardiac function was evaluated by fractional shortening (FS%, **a**), and heart rate (**f**) of α-MHC WT and α-MHC K1897R mice after NaCl or Ang II treatment. **b, g** Cardiac function was evaluated by fractional shortening (FS%, **b**), and heart rate (**g**) of LDHA-cWT and LDHA-cKO mice after NaCl or Ang II treatment. **c, h** Cardiac function was evaluated by fractional shortening (FS%, **c**), and heart rate (**h**) of WT and Ang II groups with NaCl or NALA. **d, i** Cardiac function was evaluated

by fractional shortening (FS%, **d**), and heart rate (**i**) of α-MHC WT mice (given control treatment or Ang II infusion) and α-MHC K1897R mutant mice (given control treatment, Ang II infusion, NALA infusion or combined Ang II and NALA infusion). **e** Cardiac function was evaluated by fractional shortening (FS%) of α-MHC WT mice and α-MHC K1897R mutant mice (given control treatment, Ang II infusion, VB124 infusion or combined Ang II and VB124 infusion). (**a-i)** Data are expressed as means ± SD. Statistical significance was assessed by 2-way ANOVA with Bonferroni multiple comparisons test (**a-c, f-h** P values adjusted for 6 comparisons, **d-e, i** P values adjusted for 10 comparisons; ns P >0.05 which means no significance; ** P < 0.01; *** P < 0.001).
