# Supplementary material for: α-myosin heavy chain lactylation maintains sarcomeric structure and function and alleviates the development of heart failure
Source: Cell Res. 2023 Jul 13;33(9):679–98. doi: 10.1038/s41422-023-00844-w (PMC10474270; doi:10.1038/s41422-023-00844-w)
Supplement: Supplementary file 9 — Supplementary information, Table S1 [file 41422_2023_844_MOESM9_ESM.pdf]

Supplementary information, Table S1 The lactylation levels of all 142 overlapping lactated proteins between control group and Ang II-induced heart failure group (n = 3).

Protein description	Control 1	Control 2	Control 3	Ang II 1	Ang II 2	Ang II 3	Fold change	*P* value
Acaa2	0.979	1.055	1.124	1.039	0.852	1.041	0.928	0.377
Acad10	-	0.999	-	-	0.987	-	0.988	-
Acad9	-	-	1.047	0.963	-	-	0.920	-
Acadl	0.927	1.196	1.172	0.928	0.976	0.885	0.846	0.135
Acadm	1.275	1.046	0.893	0.963	0.916	0.723	0.810	0.201
Acadvl	1.009	1.062	1.206	0.993	0.884	0.819	0.823	0.065
Acat1	1.138	1.021	1.120	1.163	0.845	0.725	0.833	0.233
Aco2	1.123	1.131	1.097	0.982	0.894	0.824	0.806	0.013
Acot13	0.999	1.238	1.078	1.217	0.759	0.895	0.866	0.354
Actc1	1.213	1.272	1.027	0.919	0.851	0.811	0.735	0.015
Actn2	1.221	1.302	1.112	0.780	0.656	0.890	0.640	0.010
Ak1	1.082	1.092	0.959	1.054	0.843	0.999	0.924	0.364
Alb	1.106	1.187	1.019	0.881	0.882	0.961	0.823	0.021
Aldh2	0.970	-	-	1.295	-	0.872	1.118	-
Aldoa	1.101	1.060	1.086	0.910	0.911	0.976	0.861	0.004
Atp1a1	-	-	1.117	0.918	-	-	0.822	-
Atp1a2	1.322	-	1.238	0.901	0.878	0.726	0.652	0.018
Atp2a2	0.858	1.325	1.290	1.013	0.848	0.765	0.756	0.175
Atp5f1a	1.072	1.270	0.980	1.022	0.889	0.743	0.799	0.130
Atp5f1b	1.101	1.304	1.107	0.965	0.750	0.797	0.715	0.023
Atp5f1c	1.126	1.151	1.094	0.904	0.796	-	0.756	0.012
Atp5f1d	0.966	1.264	1.010	1.021	0.869	0.927	0.869	0.230
Atp5f1e	1.118	1.211	1.114	1.079	0.766	0.692	0.737	0.078
ATP5IF1	0.862	2.293	1.576	0.921	1.019	0.393	0.493	0.162
Atp5mf	-	-	1.248	1.060	0.974	0.782	0.752	-
Atp5pd	1.143	1.162	1.052	1.026	0.844	0.792	0.793	0.047
Atp5pf	0.915	0.924	1.184	1.053	1.004	-	1.021	0.819
Atp5po	1.066	1.144	1.101	1.051	0.824	0.735	0.788	0.081
Ckm	1.108	1.256	1.135	0.988	0.782	0.839	0.745	0.020
Ckmt2	1.186	1.325	0.988	0.899	0.863	0.556	0.663	0.072
Clybl	1.236	0.909	-	1.013	-	-	0.945	-
Cox4i1	1.364	1.202	1.111	1.102	0.714	0.778	0.705	0.067
Cox5b	1.148	1.116	1.154	1.021	0.826	0.796	0.773	0.075
Cox7a1	1.010	1.183	1.047	1.152	0.916	0.737	0.866	0.314
Crat	1.158	-	-	-	1.073	0.685	0.759	-
Cryab	-	1.055	-	1.134	-	-	1.075	-
Cs	1.074	1.138	1.115	1.140	0.858	0.725	0.819	0.240
Csrp3	0.853	0.943	0.925	1.146	0.957	1.076	1.168	0.066
Decr1	0.983	0.968	1.112	1.104	0.992	0.870	0.968	0.691

Des	1.175	1.589	0.988	0.790	0.933	0.643	0.630	0.061
Dlat	1.116	1.125	1.196	0.992	0.828	0.809	0.765	0.016
Dld	1.105	1.180	1.018	0.948	0.929	0.859	0.828	0.023
Dlst	0.948	1.154	1.201	1.318	0.715	0.744	0.841	0.377
Ech1	0.932	1.161	1.241	1.120	0.762	0.825	0.812	0.214
Echs1	1.039	1.050	1.247	0.844	0.892	0.924	0.797	0.026
Eci1	0.921	1.187	1.265	0.957	0.884	0.757	0.770	0.097
Eci2	1.081	1.083	1.135	1.065	0.679	-	0.793	0.457
Eno3	1.291	1.128	1.047	0.805	0.866	0.810	0.716	0.007
Etfa	1.021	1.187	1.136	1.050	0.848	0.776	0.800	0.084
Etfb	0.953	1.130	1.098	1.182	0.905	0.776	0.900	0.427
Etfdh	1.132	1.085	1.162	1.001	0.795	0.819	0.774	0.025
Fabp3	0.968	1.587	1.054	0.946	0.888	0.882	0.753	0.228
Fabp4	-	0.948	1.253	-	0.907	0.932	0.836	0.349
Fh	1.254	1.096	1.026	0.938	0.865	0.807	0.773	0.025
Gapdh	1.286	1.044	1.125	0.969	0.857	0.877	0.782	0.027
Gatd3a	1.042	1.134	1.069	1.089	0.847	0.821	0.850	0.140
Got2	1.119	1.121	1.100	0.964	0.848	0.879	0.806	0.027
H3-3a	0.968	1.011	1.151	0.853	0.951	0.991	0.893	0.177
Hadh	0.984	1.139	1.065	1.037	0.846	0.961	0.892	0.187
Hadha	1.056	1.099	1.121	1.059	0.891	0.746	0.823	0.116
Hadhb	1.142	1.124	1.189	0.728	0.938	0.776	0.707	0.011
Hba	1.177	1.091	1.013	1.043	0.990	0.763	0.852	0.191
Hbb-b1	0.984	0.900	1.096	0.868	1.136	0.955	0.993	0.925
Hibch	1.069	1.120	1.089	0.983	0.699	-	0.770	0.351
Hint2	1.064	1.083	-	1.283	0.812	0.851	0.915	0.596
Hsd17b10	0.969	1.414	1.349	0.775	0.774	0.790	0.627	0.062
Hsdl2	1.175	1.092	-	1.112	0.786	0.749	0.778	0.201
Hspa2	-	0.838	0.859	1.126	1.029	1.061	1.263	0.007
Hspa9	1.107	1.242	1.368	0.892	0.765	0.651	0.621	0.012
Hspd1	1.045	1.149	1.203	1.057	0.840	0.742	0.777	0.079
Hspe1	1.162	1.203	0.847	1.152	0.841	0.779	0.863	0.412
Idh2	1.185	1.134	1.076	1.007	0.767	0.748	0.743	0.037
Idh3a	1.121	1.128	1.148	1.041	0.839	0.791	0.786	0.096
Immt	-	1.051	1.325	0.807	1.024	0.922	0.773	0.131
Ivd	0.972	1.093	1.057	1.004	0.973	0.948	0.937	0.171
Ldb3	1.185	1.147	0.886	0.989	0.935	0.918	0.883	0.285
Letm1	0.871	1.349	1.285	-	0.718	0.840	0.667	0.130
Macrod1	0.942	1.012	1.330	1.059	0.650	0.788	0.760	0.184
Mb	1.113	1.169	0.974	1.005	0.975	0.846	0.868	0.137
Mccc1	1.027	1.120	1.153	0.831	0.752	0.886	0.748	0.008
Mcee	1.433	1.050	1.056	1.307	0.747	0.596	0.749	0.259
Mdh1	1.151	1.254	0.958	0.866	0.862	0.948	0.796	0.059
Mdh2	1.096	0.978	1.047	1.069	0.899	0.956	0.937	0.331

Mrpl12	1.113	0.923	-	0.990	-	-	0.972	-
Msra	-	-	1.112	-	-	0.824	0.741	-
Mtatp8	1.048	1.302	1.024	0.986	-	0.736	0.766	0.162
Mybpc3	1.184	1.139	1.107	0.954	0.921	0.828	0.788	0.007
Myh3	2.526	0.586	1.840	0.501	0.194	0.805	0.303	0.124
Myh4	1.445	0.923	0.942	0.709	0.852	0.929	0.752	0.182
α-MHC	1.127	1.206	1.038	1.002	0.876	0.805	0.796	0.040
Myh7	2.741	0.977	1.317	-	1.427	0.732	0.643	0.456
Myh7b	1.280	1.155	0.768	0.995	1.022	0.851	0.895	0.614
Myl2	1.192	1.060	1.004	0.884	0.969	0.883	0.840	0.044
Myl3	1.202	1.081	0.919	1.050	0.887	0.882	0.880	0.267
Ndufa10	0.532	1.333	0.894	1.514	-	-	1.647	-
Ndufa2	1.173	1.063	1.125	1.154	0.746	0.797	0.802	0.161
Ndufa4	-	1.221	1.227	0.901	0.856	0.802	0.697	0.004
Ndufa5	1.228	1.080	1.130	0.893	0.728	0.772	0.696	0.007
Ndufa7	1.217	1.132	1.184	1.128	0.687	0.711	0.715	0.150
Ndufa9	-	1.317	1.129	1.042	-	0.685	0.706	0.242
Ndufab1	1.188	0.981	1.234	0.900	-	0.842	0.768	0.072
Ndufs1	0.859	1.082	1.176	1.021	0.997	0.890	0.933	0.577
Ndufs2	1.120	1.233	-	0.707	-	-	0.601	-
Ndufs6	1.274	1.164	1.021	1.088	0.802	0.788	0.774	0.099
Ndufs8	1.014	1.166	1.180	1.304	0.682	0.722	0.806	0.291
Nipsnap2	1.043	1.150	1.120	1.020	0.650	0.750	0.730	0.072
Nnt	-	0.788	-	-	-	0.689	0.874	-
Ogdh	-	1.218	0.996	1.130	-	0.748	0.848	0.514
Oxct1	1.066	1.269	1.121	1.179	0.729	0.745	0.768	0.157
Pcca	0.962	1.383	0.976	1.012	-	0.744	0.793	0.317
Pdha1	0.839	1.168	1.125	1.126	0.619	1.021	0.883	0.531
Pdhx	1.353	-	1.195	-	-	0.610	0.479	-
Pfkm	0.942	1.007	0.923	1.028	1.014	1.066	1.082	0.058
Pfkp	-	0.791	-	-	0.933	0.935	1.181	-
Pgam2	1.209	1.305	1.263	0.775	0.653	0.764	0.580	0.001
Ppif	1.095	0.998	-	1.280	-	0.718	0.954	0.795
Prdx5	1.219	1.116	1.069	1.068	0.764	0.887	0.799	0.090
Sdha	1.108	1.197	0.970	1.246	0.820	0.735	0.855	0.356
Sdhb	1.129	1.102	1.075	1.093	0.846	0.653	0.784	0.215
Slc25a11	1.016	0.816	0.981	0.933	1.275	1.009	1.144	0.325
Slc25a3	1.140	1.069	1.244	1.036	0.816	0.698	0.738	0.063
Slc25a4	1.068	1.278	1.101	0.953	0.902	0.766	0.760	0.033
Slc25a5	1.018	-	-	0.925	-	-	0.909	-
Sod2	0.941	1.198	1.102	0.866	0.840	1.108	0.868	0.270
Sucla2	1.091	1.143	1.204	1.180	0.802	0.625	0.758	0.233
Suclg1	0.965	1.158	1.276	1.119	0.818	0.768	0.796	0.173
Suclg2	1.419	0.644	0.804	1.159	1.062	0.683	1.013	0.883

Tnni3	0.998	1.091	1.088	1.130	0.888	0.773	0.878	0.286
Tnnt2	1.344	1.098	1.110	0.883	0.860	0.691	0.685	0.020
Tpm1	0.996	1.128	1.216	1.115	0.803	0.724	0.791	0.156
Tpm3	1.167	1.291	0.943	1.062	0.912	0.790	0.813	0.176
Tpm4	1.260	1.097	1.391	1.056	0.781	0.698	0.677	0.047
Trap1	0.807	0.759	0.852	1.280	1.157	1.159	1.487	0.001
Tsfm	-	0.841	-	1.308	-	-	1.555	-
Ttn	1.057	1.144	1.129	1.023	0.865	0.825	0.815	0.040
Tufm	1.058	1.106	1.087	1.018	0.754	0.952	0.838	0.177
Uqcrb	1.050	1.180	1.333	1.033	0.744	0.737	0.706	0.052
Uqcrc2	1.471	1.287	1.154	1.017	0.668	0.731	0.617	0.028
Uqcrfs1	1.227	-	1.008	1.070	-	0.656	0.772	0.395
Uqcrh	-	-	1.244	1.181	0.942	0.814	0.787	-
Vdac3	1.141	1.041	0.996	0.962	-	-	0.908	-
Atp5pb	1.171	1.143	0.973	1.107	0.830	0.825	0.840	0.186
