# Supplementary material for: α-myosin heavy chain lactylation maintains sarcomeric structure and function and alleviates the development of heart failure
Source: Cell Res. 2023 Jul 13;33(9):679–98. doi: 10.1038/s41422-023-00844-w (PMC10474270; doi:10.1038/s41422-023-00844-w)
Supplement: Supplementary file 10 — Supplementary information, Table S2 [file 41422_2023_844_MOESM10_ESM.pdf]

Supplementary information, Table S2 Differential analysis of all 483 overlapping lactylated modified sites between control group and Ang II-induced heart failure group (n = 3).

Protein description	Position	Amino acid	Control 1	Control 2	Control 3	Ang II 1	Ang II 2	Ang II 3	Fold change	*P* value
Acaa2	137	K	1.214	-	-	-	0.718	-	0.591	-
Acaa2	214	K	0.814	1.020	1.140	1.143	0.941	0.970	1.027	0.794
Acaa2	241	K	0.914	-	-	-	0.896	1.111	1.098	-
Acaa2	270	K	0.972	1.089	1.108	0.934	-	-	0.884	-
Acad10	1056	K	-	0.999	-	-	0.987	-	0.988	-
Acad9	242	K	-	-	1.047	0.963	-	-	0.920	-
Acadl	66	K	0.800	-	-	-	1.137	0.956	1.308	-
Acadl	81	K	1.047	1.207	1.112	0.915	0.979	0.789	0.797	0.039
Acadl	95	K	0.828	1.192	1.204	0.876	0.933	1.011	0.875	0.404
Acadl	322	K	1.034	1.190	1.201	0.994	0.853	0.784	0.768	0.035
Acadm	30	K	1.741	0.865	0.503	0.787	1.064	-	0.893	0.994
Acadm	212	K	1.035	1.149	1.122	1.079	-	0.723	0.818	0.250
Acadm	259	K	1.049	1.124	1.053	1.024	0.762	-	0.830	0.183
Acadvl	72	K	-	-	1.143	-	0.887	-	0.776	-
Acadvl	122	K	0.871	1.094	1.155	1.023	-	-	0.984	-
Acadvl	196	K	1.010	1.169	1.168	1.164	0.774	0.771	0.809	0.190
Acadvl	277	K	1.017	1.175	1.199	1.048	0.903	0.713	0.786	0.112
Acadvl	483	K	0.890	1.111	1.792	0.883	0.777	0.673	0.615	0.112
Acadvl	557	K	1.097	0.748	-	0.847	1.080	1.118	1.100	0.600
Acat1	171	K	0.972	1.005	1.214	1.396	0.921	0.515	0.887	0.549
Acat1	199	K	1.236	-	-	-	0.719	0.965	0.681	-
Acat1	260	K	1.127	-	1.131	1.137	0.788	0.777	0.798	0.230
Acat1	265	K	1.290	1.036	1.151	0.955	0.950	0.644	0.733	0.090
Aco2	31	K	1.041	1.065	1.146	1.055	0.907	0.825	0.857	0.109
Aco2	50	K	1.144	1.086	1.133	0.913	0.942	0.819	0.795	0.007
Aco2	138	K	1.263	1.261	0.931	0.990	0.852	0.757	0.752	0.093
Aco2	401	K	1.508	0.945	1.148	0.882	0.905	0.648	0.676	0.091
Aco2	411	K	-	1.181	1.037	0.905	-	0.982	0.851	0.172
Aco2	517	K	1.053	1.110	1.196	1.069	0.832	0.793	0.802	0.084
Aco2	523	K	-	1.231	-	1.033	-	0.816	0.751	-
Aco2	549	K	1.068	1.157	1.037	0.975	0.844	-	0.836	0.078
Aco2	591	K	-	-	1.205	0.934	0.873	0.941	0.760	-
Aco2	605	K	0.764	1.190	1.150	-	1.024	0.893	0.926	0.775
Aco2	730	K	1.143	1.084	1.110	1.068	0.871	0.770	0.812	0.088
Acot13	127	K	1.123	1.238	1.106	1.060	0.741	0.808	0.753	0.060
Actc1	52	K	1.309	1.215	1.028	0.833	0.761	0.939	0.713	0.023
Actc1	63	K	1.268	1.202	1.144	0.967	0.784	0.731	0.687	0.013
**Actc1**	**115**	**K**	**1.546**	**1.283**	**1.018**	**0.826**	**0.787**	**0.657**	**0.590**	**0.021**
Actc1	317	K	0.983	-	0.972	-	1.040	0.917	1.001	0.989

Actc1	328	K	0.960	1.387	0.973	1.050	0.883	0.809	0.826	0.271
Actn2	102	K	-	1.302	-	0.780	-	0.890	0.641	-
Actn2	443	K	1.391	-	-	-	0.656	-	0.472	-
Ak1	21	K	1.003	1.010	0.871	-	0.948	1.153	1.093	0.435
Ak1	27	K	1.161	1.173	1.046	1.090	0.737	0.845	0.791	0.109
Alb	212	K	-	1.359	0.856	0.991	0.950	-	0.876	0.693
Alb	223	K	1.010	1.011	1.237	-	0.839	-	0.773	-
Alb	264	K	-	1.364	0.991	1.108	0.764	0.837	0.767	0.251
Alb	376	K	-	-	1.115	-	0.774	-	0.694	-
Alb	413	K	-	1.113	1.224	0.924	0.866	-	0.766	0.044
**Alb**	**490**	**K**	**1.154**	**1.318**	**1.222**	**0.808**	**-**	**0.727**	**0.623**	**0.005**
Alb	543	K	-	-	0.723	-	1.113	-	1.539	-
Alb	549	K	1.114	1.198	1.057	-	-	0.769	0.685	-
Alb	588	K	1.144	0.944	0.746	0.572	-	1.605	1.152	0.948
Aldh2	370	K	0.768	-	-	1.295	-	-	1.686	-
Aldh2	430	K	1.171	-	-	-	-	0.872	0.745	-
Aldoa	42	K	1.206	1.019	1.116	0.952	0.869	0.883	0.809	0.020
Aldoa	147	K	0.995	1.011	1.041	0.923	0.975	1.049	0.967	0.429
Aldoa	230	K	-	1.040	0.899	0.855	1.056	1.074	1.026	0.844
Aldoa	322	K	-	1.314	-	-	0.743	0.898	0.624	-
Atp1a1	21	K	-	-	1.117	0.918	-	-	0.822	-
**Atp1a2**	**658**	**K**	**1.322**	**-**	**1.238**	**0.901**	**0.878**	**0.726**	**0.652**	**0.018**
Atp2a2	205	K	0.966	1.292	-	-	-	0.879	0.779	-
Atp2a2	502	K	0.750	1.108	1.176	0.988	0.934	1.051	0.980	0.987
Atp2a2	712	K	-	1.576	1.404	1.037	0.761	0.364	0.484	0.137
Atp5f1a	161	K	1.073	1.409	0.262	1.128	1.148	-	1.244	0.562
Atp5f1a	167	K	1.056	1.201	1.094	1.148	0.807	0.751	0.808	0.167
Atp5f1a	218	K	-	1.356	-	0.760	0.938	-	0.626	-
**Atp5f1a**	**230**	**K**	**1.344**	**1.200**	**1.162**	**0.912**	**0.867**	**0.555**	**0.630**	**0.042**
Atp5f1a	239	K	-	1.244	-	0.898	-	-	0.722	-
Atp5f1a	427	K	1.022	1.321	1.015	1.141	0.811	0.758	0.807	0.219
Atp5f1a	498	K	0.989	1.228	1.032	1.023	0.946	0.826	0.860	0.175
Atp5f1a	531	K	0.966	1.230	1.161	1.052	0.848	0.802	0.805	0.118
Atp5f1a	539	K	1.054	1.241	1.131	1.132	0.745	0.764	0.771	0.126
Atp5f1b	124	K	1.031	1.286	1.194	1.029	0.741	0.791	0.729	0.055
Atp5f1b	201	K	1.122	1.351	0.984	0.933	-	0.741	0.726	0.119
Atp5f1b	259	K	1.238	-	-	0.734	-	-	0.593	-
Atp5f1b	426	K	-	1.449	1.153	0.869	0.685	0.957	0.643	0.063
Atp5f1b	522	K	1.042	1.166	1.222	1.202	0.685	0.752	0.769	0.181
Atp5f1c	55	K	1.317	-	-	0.664	-	-	0.504	-
Atp5f1c	83	K	-	1.151	-	1.070	0.796	-	0.811	-
Atp5f1d	136	K	0.966	1.264	1.010	1.021	0.869	0.927	0.869	0.230
Atp5f1e	32	K	1.072	1.165	1.090	1.039	0.740	-	0.802	0.171
Atp5f1e	37	K	1.041	1.298	1.120	1.072	0.824	0.712	0.754	0.098

Atp5f1e	44	K	1.241	1.171	1.132	1.127	0.735	0.672	0.715	0.089
ATP5IF1	64	K	1.087	2.456	1.507	0.921	0.832	0.393	0.425	0.073
Atp5mf	8	K	-	-	1.248	1.060	0.974	0.782	0.752	-
Atp5pb	144	K	0.930	0.936	-	1.221	0.934	0.920	1.099	0.524
Atp5pb	225	K	1.103	1.255	1.257	0.992	0.761	0.718	0.684	0.023
Atp5pb	233	K	-	1.273	1.145	-	0.794	0.836	0.674	0.022
Atp5pd	63	K	-	1.225	1.198	0.994	0.832	0.791	0.720	0.034
Atp5pd	72	K	1.083	-	1.121	1.003	0.780	-	0.809	0.226
Atp5pd	78	K	0.977	1.260	1.204	1.014	0.841	0.763	0.761	0.075
Atp5pd	85	K	1.202	-	0.961	0.781	1.020	-	0.833	0.398
Atp5pd	95	K	1.228	1.067	1.091	1.032	0.855	0.768	0.784	0.062
Atp5pd	99	K	1.184	1.227	0.907	1.085	0.868	0.768	0.820	0.224
Atp5pd	117	K	1.071	1.102	1.181	1.172	0.752	0.775	0.805	0.179
Atp5pd	149	K	-	-	1.065	0.967	-	-	0.908	-
Atp5pf	84	K	1.054	0.924	-	0.966	1.004	-	0.996	0.979
Atp5pf	94	K	0.694	-	1.184	1.140	-	-	1.214	-
**Atp5po**	**51**	**K**	**1.342**	**1.413**	**1.044**	**0.920**	**0.661**	**0.695**	**0.599**	**0.021**
Atp5po	70	K	1.099	1.144	1.029	1.048	0.889	0.828	0.845	0.087
Atp5po	90	K	1.212	1.094	1.148	1.180	0.743	0.682	0.754	0.146
Ckm	9	K	-	1.352	0.972	1.109	0.826	0.783	0.780	0.276
Ckm	15	K	1.002	-	1.219	-	0.794	-	0.715	-
Ckm	107	K	1.214	-	1.215	0.963	0.725	0.826	0.690	0.038
Ckm	242	K	-	1.265	-	0.883	-	-	0.698	-
Ckmt2	149	K	1.334	-	1.063	-	0.692	-	0.577	-
Ckmt2	209	K	1.341	1.352	0.912	0.899	1.034	0.556	0.690	0.165
Cox4i1	29	K	1.364	1.010	1.004	1.169	0.755	0.753	0.792	0.241
Cox4i1	67	K	-	1.264	1.256	1.034	0.706	0.803	0.673	0.066
Cox4i1	78	K	-	1.332	1.084	-	0.682	-	0.565	-
Cox5b	67	K	1.175	-	1.149	0.970	0.876	0.775	0.752	0.041
Cox5b	73	K	1.121	1.116	1.158	1.072	0.775	0.817	0.785	0.067
Cox7a1	31	K	1.010	1.183	1.047	1.152	0.916	0.737	0.866	0.314
Crat	186	K	1.158	-	-	-	1.073	0.685	0.759	-
Cryab	90	K	-	1.055	-	1.134	-	-	1.075	-
Cs	43	K	1.062	1.094	1.069	1.120	0.911	0.798	0.877	0.228
Cs	103	K	1.066	1.205	1.018	0.943	0.893	-	0.837	0.082
Cs	321	K	1.094	1.069	1.139	1.223	0.795	0.752	0.839	0.266
Cs	366	K	1.081	1.274	1.070	-	-	0.663	0.581	-
Cs	370	K	-	1.182	1.109	1.148	0.845	0.770	0.804	0.236
Cs	450	K	1.069	1.176	1.278	1.145	0.778	0.644	0.729	0.125
Cs	459	K	-	1.009	1.122	1.340	0.857	0.725	0.914	0.643
Csrp3	109	K	0.711	0.906	0.939	1.149	0.999	1.146	1.289	0.059
Csrp3	113	K	0.995	0.980	0.910	1.142	0.914	1.005	1.061	0.471
Decr1	97	K	0.988	0.777	0.999	1.087	1.179	0.975	1.173	0.175
Decr1	106	K	0.978	1.158	1.225	1.121	0.804	0.765	0.800	0.166

Des	124	K	1.204	1.164	1.023	0.804	0.933	0.939	0.789	0.029
Des	298	K	0.599	2.548	-	-	-	0.347	0.221	-
Des	308	K	1.723	1.056	0.953	0.776	-	-	0.624	-
Dlat	363	K	-	1.250	-	1.079	0.834	0.807	0.725	-
Dlat	371	K	1.217	0.994	1.182	0.982	0.856	0.811	0.781	0.044
Dlat	468	K	-	1.132	1.210	0.914	0.794	-	0.729	0.055
Dld	66	K	-	1.121	1.028	1.034	-	0.857	0.880	0.332
Dld	132	K	0.990	1.197	1.102	0.956	0.955	0.832	0.834	0.066
Dld	143	K	1.058	1.266	1.136	0.925	0.842	0.818	0.747	0.011
Dld	430	K	0.937	1.136	1.200	0.841	0.989	0.929	0.843	0.132
Dlst	155	K	0.838	1.100	1.249	1.456	0.725	0.710	0.907	0.616
Dlst	218	K	1.058	1.207	1.152	1.180	0.705	0.778	0.779	0.167
Ech1	76	K	0.932	1.161	1.241	1.120	0.762	0.825	0.812	0.214
Echs1	43	K	0.960	1.105	1.283	0.771	0.979	0.924	0.799	0.114
Echs1	101	K	1.118	0.954	1.210	0.916	0.805	-	0.787	0.103
Eci1	76	K	0.976	1.094	1.174	0.958	-	-	0.886	-
Eci1	255	K	0.866	1.279	1.355	0.955	-	0.757	0.734	0.236
Eci2	60	K	1.081	1.083	1.135	1.065	0.679	-	0.793	0.226
Eno3	28	K	1.714	-	0.606	-	-	0.714	0.616	-
Eno3	64	K	1.233	-	-	0.857	0.863	-	0.697	-
Eno3	71	K	-	-	1.397	0.683	0.868	-	0.555	-
Eno3	420	K	1.189	1.072	1.139	0.875	0.867	-	0.769	0.007
Etfa	59	K	1.002	1.167	1.004	1.023	0.935	0.907	0.903	0.180
Etfa	62	K	0.925	1.317	-	1.098	0.688	-	0.797	0.500
Etfa	75	K	0.931	-	-	-	1.025	-	1.101	-
Etfa	206	K	0.985	-	-	1.004	-	-	1.019	-
**Etfa**	**216**	**K**	**1.227**	**1.238**	**1.193**	**-**	**0.756**	**0.627**	**0.567**	**0.004**
Etfa	226	K	1.056	1.089	1.171	1.082	0.848	0.793	0.821	0.108
Etfa	294	K	-	1.122	1.175	1.044	0.710	-	0.764	0.276
Etfb	26	K	0.825	1.018	-	1.349	-	0.881	1.210	0.541
Etfb	116	K	-	-	1.017	1.135	0.962	0.845	0.964	-
Etfb	238	K	-	1.190	1.162	1.109	-	0.663	0.753	0.345
Etfb	248	K	1.050	1.182	1.115	1.136	0.847	0.716	0.806	0.170
Etfdh	222	K	1.132	1.138	1.162	1.001	0.795	0.819	0.762	0.019
Fabp3	15	K	0.637	-	1.189	1.226	0.905	0.882	1.100	0.659
Fabp3	91	K	1.208	1.587	0.919	0.666	0.871	-	0.621	0.135
Fabp4	121	K	-	0.948	1.253	-	0.907	0.932	0.836	0.349
Fh	58	K	1.716	0.722	0.500	0.814	1.130	1.082	1.030	0.699
Fh	63	K	1.007	1.219	1.141	1.060	0.654	-	0.764	0.225
Fh	169	K	-	1.232	1.016	0.891	-	-	0.793	-
Fh	170	K	1.199	-	1.237	-	-	0.610	0.501	-
Fh	220	K	1.094	1.212	1.229	0.988	0.810	0.728	0.715	0.024
Gapdh	192	K	-	0.977	1.089	0.994	0.939	0.937	0.926	0.210
Gapdh	213	K	1.286	1.111	1.160	0.943	0.774	0.817	0.712	0.010

Gatd3a	149	K	1.123	1.117	0.987	1.251	0.803	0.792	0.882	0.398
Gatd3a	155	K	-	1.014	1.092	0.981	-	-	0.932	-
Gatd3a	162	K	-	1.319	-	0.838	-	-	0.635	-
Gatd3a	186	K	0.834	1.201	1.294	1.238	0.648	0.859	0.825	0.414
Gatd3a	201	K	1.004	0.893	0.868	1.170	1.091	1.022	1.187	0.044
Gatd3a	217	K	1.143	1.099	-	0.980	0.859	-	0.820	0.101
Gatd3a	221	K	1.043	1.158	1.108	1.224	0.809	0.723	0.833	0.270
Gatd3a	231	K	1.107	1.267	1.063	1.031	0.869	0.710	0.759	0.077
Got2	82	K	1.315	-	0.968	0.958	0.877	0.856	0.786	0.156
Got2	90	K	1.182	1.249	-	0.922	0.849	0.834	0.714	0.005
Got2	122	K	1.085	1.095	1.108	0.994	0.849	0.910	0.837	0.018
Got2	338	K	1.064	1.151	1.138	1.043	0.840	0.812	0.804	0.053
Got2	363	K	1.067	-	1.192	0.903	0.826	-	0.765	0.064
H3-3a	24	K	0.989	1.131	1.048	0.951	0.917	-	0.884	0.102
H3-3a	80	K	0.946	0.911	1.416	0.754	0.985	0.991	0.834	0.371
Hadh	68	K	-	1.152	1.141	-	0.848	-	0.740	-
Hadh	81	K	0.986	1.181	1.113	-	0.805	-	0.736	-
Hadh	87	K	1.022	-	1.050	1.052	0.885	0.918	0.919	0.292
Hadh	312	K	1.037	1.051	0.954	1.021	-	-	1.007	-
Hadha	46	K	1.042	1.054	1.195	0.950	0.755	-	0.777	0.088
Hadha	60	K	0.894	1.203	1.287	1.116	0.823	0.738	0.791	0.227
Hadha	262	K	-	1.138	1.368	-	-	0.637	0.508	-
Hadha	284	K	-	1.257	0.972	0.891	-	-	0.799	-
Hadha	295	K	0.901	1.057	1.164	1.099	0.783	-	0.904	0.540
Hadha	309	K	1.028	-	-	-	1.059	0.800	0.904	-
Hadha	350	K	-	-	0.356	-	1.514	-	4.253	-
Hadha	353	K	1.022	1.225	-	1.029	0.906	0.789	0.808	0.171
Hadha	390	K	1.512	1.082	1.025	1.014	-	0.430	0.599	0.198
Hadha	406	K	1.002	1.186	1.391	1.014	0.753	0.714	0.693	0.063
Hadha	415	K	1.090	1.109	1.253	0.984	0.863	0.735	0.748	0.036
Hadha	489	K	-	1.180	1.068	1.028	0.932	0.885	0.844	0.086
Hadha	516	K	-	0.841	1.035	1.253	-	-	1.336	-
Hadha	519	K	1.079	1.071	1.187	1.073	0.809	0.826	0.812	0.089
Hadha	569	K	0.979	1.147	1.265	1.063	0.817	0.780	0.784	0.113
Hadha	634	K	1.060	1.118	1.030	1.212	0.740	0.896	0.888	0.393
Hadha	644	K	1.096	1.055	1.225	1.108	0.832	0.723	0.789	0.133
Hadhb	202	K	-	-	1.229	-	-	0.862	0.701	-
Hadhb	255	K	1.473	1.117	-	0.165	1.167	-	0.514	0.391
Hadhb	273	K	1.059	1.191	1.220	-	0.850	0.719	0.678	0.019
Hadhb	278	K	1.161	1.212	1.095	1.023	0.797	0.748	0.740	0.037
Hba	8	K	-	1.055	1.078	1.003	0.873	0.845	0.850	0.095
Hba	12	K	0.947	1.236	1.109	1.152	0.888	0.727	0.841	0.295
Hba	17	K	-	1.085	1.049	0.997	0.911	0.819	0.852	0.116
Hba	41	K	1.229	-	0.906	-	1.171	0.692	0.873	0.653

Hba	57	K	1.154	1.313	1.059	1.021	0.851	0.731	0.738	0.055
Hba	140	K	1.377	0.767	0.875	-	1.248	-	1.240	-
Hbb-b1	67	K	0.748	0.857	0.953	0.921	1.314	1.089	1.299	0.108
Hbb-b1	96	K	-	0.733	-	0.796	1.169	1.033	1.363	-
Hbb-b1	145	K	1.220	1.109	1.239	0.888	0.925	0.744	0.717	0.011
Hibch	352	K	1.069	1.120	1.232	0.983	0.699	-	0.738	0.105
Hint2	128	K	1.064	1.083	-	1.283	0.812	0.851	0.915	0.596
**Hsd17b10**	**107**	**K**	**0.969**	**1.414**	**1.349**	**0.775**	**0.774**	**0.790**	**0.627**	**0.019**
Hsdl2	390	K	1.175	-	-	1.112	0.786	0.749	0.751	-
Hspa2	72	K	-	0.838	0.859	1.126	1.029	1.061	1.263	0.007
Hspa9	135	K	1.254	1.287	1.286	1.091	0.548	0.623	0.591	0.054
Hspa9	138	K	1.070	-	1.492	-	0.825	0.613	0.561	0.123
Hspa9	300	K	0.936	-	-	-	0.999	-	1.067	-
Hspa9	567	K	1.133	1.098	1.092	1.186	-	0.587	0.800	0.360
Hspa9	600	K	-	1.360	1.810	0.286	0.699	-	0.311	0.116
Hspa9	625	K	1.141	1.223	1.159	1.006	0.753	0.781	0.721	0.023
Hspd1	133	K	0.997	1.193	1.194	1.134	0.753	0.769	0.785	0.153
Hspd1	250	K	1.093	1.104	1.212	0.979	0.927	0.714	0.769	0.058
Hspe1	40	K	0.976	1.276	1.184	1.072	0.829	0.743	0.769	0.118
Hspe1	54	K	1.451	1.198	0.108	1.410	0.921	0.926	1.181	0.506
Hspe1	56	K	1.058	1.288	1.248	1.061	0.772	0.668	0.696	0.065
Hspe1	66	K	-	1.049	-	1.066	-	-	1.016	-
Idh2	45	K	1.576	0.982	-	0.985	-	0.601	0.620	0.295
Idh2	155	K	1.099	1.082	1.163	0.874	0.812	-	0.756	0.006
Idh2	180	K	0.967	1.247	1.187	1.066	0.695	0.904	0.784	0.160
Idh2	199	K	-	1.137	1.131	-	-	0.775	0.683	-
Idh2	272	K	1.210	-	1.073	1.226	0.747	0.774	0.802	0.329
Idh2	280	K	0.977	1.097	1.064	0.982	-	-	0.939	-
Idh2	282	K	1.181	1.260	1.126	0.986	0.813	0.687	0.697	0.027
Idh2	384	K	1.288	-	0.869	0.930	-	-	0.862	-
Idh3a	58	K	1.136	1.228	1.134	0.936	0.840	0.782	0.731	0.006
Idh3a	223	K	1.147	1.170	1.130	1.111	0.759	0.747	0.759	0.088
Idh3a	336	K	-	0.802	-	1.296	-	-	1.616	-
Idh3a	343	K	1.080	1.151	1.220	0.994	0.810	0.800	0.755	0.022
Idh3a	350	K	-	1.289	1.106	0.867	0.947	0.835	0.737	0.027
Immt	435	K	-	-	1.579	0.513	-	-	0.325	-
Immt	450	K	-	1.051	-	0.929	1.024	0.922	0.912	-
Ivd	76	K	0.994	1.093	1.057	1.004	0.950	0.948	0.923	0.076
Ldb3	218	K	1.185	1.104	1.029	0.983	0.873	0.883	0.825	0.026
Ldb3	507	K	-	1.190	0.743	0.995	0.996	0.953	1.015	0.825
Letm1	458	K	0.871	1.349	1.285	-	0.718	0.840	0.667	0.130
Macrod1	101	K	1.012	-	-	0.964	-	-	0.953	-
Macrod1	131	K	0.997	1.046	1.265	1.154	0.808	0.788	0.831	0.244
Macrod1	207	K	0.818	0.978	1.676	-	0.491	-	0.424	-

Mb	43	K	-	-	0.837	-	1.133	-	1.354	-
Mb	51	K	1.090	1.118	1.079	1.078	0.978	0.708	0.841	0.213
Mb	57	K	1.065	1.242	1.148	1.085	0.812	0.716	0.756	0.089
Mb	64	K	1.293	0.799	0.980	0.890	1.046	0.988	0.952	0.839
Mb	78	K	-	-	0.688	0.947	1.189	1.130	1.582	-
Mb	80	K	1.098	1.321	1.099	0.952	0.903	0.685	0.722	0.050
Mb	97	K	1.018	1.365	0.988	1.078	0.766	0.848	0.799	0.195
Mccc1	233	K	1.027	1.064	1.148	1.172	0.752	0.886	0.868	0.303
Mccc1	692	K	-	1.175	1.352	0.489	-	-	0.387	-
Mcee	152	K	1.433	1.050	1.056	1.307	0.747	0.596	0.749	0.259
Mdh1	110	K	1.122	-	-	0.875	-	-	0.780	-
Mdh1	118	K	1.179	1.268	0.958	0.811	0.862	-	0.737	0.074
Mdh1	205	K	-	1.239	-	0.912	-	0.948	0.751	-
Mdh2	78	K	1.008	0.524	0.677	-	1.179	1.573	1.869	0.093
Mdh2	239	K	1.116	1.102	1.150	1.239	0.751	0.691	0.796	0.224
Mdh2	301	K	1.104	1.223	1.195	0.939	0.825	0.771	0.720	0.007
Mdh2	307	K	1.157	1.063	1.167	1.029	0.840	0.788	0.784	0.044
Mrpl12	181	K	1.188	0.923	-	1.016	-	-	0.963	-
Msra	183	K	-	-	1.112	-	-	0.824	0.741	-
Mtatp8	48	K	1.048	1.302	1.024	0.986	-	0.736	0.766	0.162
Mybpc3	87	K	1.135	1.150	-	0.635	-	1.148	0.780	0.429
Mybpc3	193	K	-	1.027	0.893	-	1.059	0.961	1.052	0.603
Mybpc3	414	K	-	-	1.088	1.135	-	0.740	0.862	-
**Mybpc3**	**561**	**K**	**1.234**	**1.240**	**1.233**	**1.048**	**0.782**	**0.599**	**0.655**	**0.050**
Mybpc3	1151	K	1.183	-	1.213	1.002	-	0.694	0.708	0.188
Myh3	1871	K	2.526	0.586	1.840	0.501	0.194	0.805	0.303	0.124
Myh4	857	K	1.161	-	-	-	0.883	-	0.761	-
Myh4	922	K	0.948	0.923	0.828	1.039	1.265	0.929	1.198	0.155
Myh4	1455	K	2.313	-	1.137	0.378	0.408	-	0.228	0.058
Myh7	383	K	2.534	-	-	-	1.610	-	0.635	-
Myh7	559	K	-	1.108	-	-	1.230	-	1.110	-
Myh7	942	K	2.947	0.856	1.317	-	1.441	0.732	0.637	0.533
Myh7b	958	K	1.254	1.349	0.656	0.975	1.080	0.781	0.870	0.708
Myh7b	959	K	1.305	0.960	0.880	1.014	0.964	0.920	0.921	0.610
Myl2	62	K	1.008	1.248	1.215	0.968	0.903	0.737	0.751	0.053
Myl2	111	K	0.921	1.215	1.156	1.099	0.823	0.849	0.842	0.237
Myl2	115	K	1.989	0.600	0.467	0.374	1.391	1.072	0.928	1.000
Myl2	165	K	1.098	1.035	1.178	1.096	0.759	0.874	0.824	0.147
Myl3	19	K	1.080	0.946	0.888	0.958	0.948	1.156	1.051	0.604
Myl3	42	K	-	1.061	1.127	1.100	0.865	0.815	0.847	0.244
Myl3	50	K	1.540	-	0.485	1.075	-	-	1.062	-
Myl3	79	K	1.294	0.999	1.069	-	0.830	-	0.741	-
Myl3	109	K	1.105	1.344	0.959	1.040	0.874	0.750	0.782	0.145
Myl3	134	K	0.952	1.188	0.984	1.032	1.056	0.805	0.926	0.514

Ndufa10	122	K	-	-	0.620	1.514	-	-	2.442	-
Ndufa2	13	K	1.173	1.063	1.125	1.154	0.746	0.797	0.802	0.161
Ndufa4	10	K	-	1.213	1.227	-	0.856	0.802	0.680	0.007
Ndufa4	74	K	-	1.228	-	0.901	0.856	-	0.715	-
Ndufa5	40	K	1.183	1.162	1.131	1.024	0.758	0.772	0.735	0.032
**Ndufa5**	**46**	**K**	**1.272**	**1.071**	**1.128**	**0.761**	**0.697**	**-**	**0.630**	**0.008**
Ndufa7	80	K	1.217	1.132	1.184	1.128	0.687	0.711	0.715	0.088
Ndufa9	175	K	-	-	1.129	1.042	-	-	0.923	-
Ndufa9	370	K	-	1.317	-	-	-	0.685	0.520	-
Ndufab1	97	K	1.188	0.981	1.234	0.900	-	0.842	0.768	0.072
Ndufs1	84	K	0.935	0.977	1.106	1.105	0.929	0.982	0.999	0.992
Ndufs1	450	K	0.782	1.132	1.246	-	1.064	0.798	0.884	0.627
Ndufs1	467	K	-	1.138	-	0.937	-	-	0.823	-
Ndufs2	425	K	1.120	1.233	-	0.707	-	-	0.601	-
Ndufs6	41	K	1.274	-	-	-	0.798	0.788	0.622	-
Ndufs6	100	K	-	1.164	1.021	1.088	0.805	-	0.866	0.451
Ndufs8	42	K	1.070	1.053	1.208	1.429	0.615	0.691	0.821	0.371
Ndufs8	51	K	0.958	1.278	1.152	1.179	0.749	0.753	0.791	0.220
Nipsnap2	53	K	1.157	-	1.101	-	0.663	-	0.587	-
Nipsnap2	63	K	0.937	1.072	-	1.066	-	-	1.061	-
Nipsnap2	94	K	0.996	1.229	1.147	0.970	-	0.750	0.765	0.117
Nipsnap2	140	K	1.082	1.150	1.112	1.025	0.637	-	0.746	0.170
Nnt	1079	K	-	0.788	-	-	-	0.689	0.874	-
Ogdh	564	K	-	1.221	-	-	-	0.809	0.663	-
Ogdh	999	K	-	1.372	1.020	-	-	0.686	0.574	-
Ogdh	1000	K	-	-	0.932	1.130	-	-	1.212	-
Oxct1	176	K	1.090	1.203	1.089	1.198	0.793	0.729	0.804	0.199
Oxct1	446	K	1.041	1.187	1.153	1.159	0.790	0.761	0.802	0.164
Oxct1	455	K	-	1.416	-	-	0.604	-	0.427	-
Pcca	184	K	0.962	1.383	0.976	1.012	-	0.744	0.793	0.317
Pdha1	63	K	0.668	-	-	-	-	1.258	1.883	-
Pdha1	244	K	-	-	1.005	1.043	-	-	1.038	-
Pdha1	313	K	1.010	1.224	1.244	1.208	0.619	0.784	0.751	0.195
Pdhx	218	K	1.353	-	1.195	-	-	0.610	0.479	-
Pfkm	678	K	0.942	1.007	0.923	1.028	1.014	1.066	1.082	0.058
Pfkp	687	K	-	0.791	-	-	0.933	0.935	1.181	-
Pgam2	100	K	-	1.428	1.435	0.395	-	-	0.276	-
Pgam2	251	K	1.209	1.182	1.256	1.029	0.653	0.764	0.671	0.036
Ppif	85	K	-	0.998	-	1.065	-	-	1.067	-
Prdx5	79	K	1.184	1.085	1.069	1.068	0.759	0.871	0.808	0.100
Prdx5	112	K	1.254	1.146	-	-	0.769	0.902	0.696	0.058
Sdha	179	K	1.087	1.103	1.096	1.172	0.804	0.788	0.841	0.215
Sdha	182	K	1.310	1.255	-	1.064	0.763	0.637	0.640	0.095
Sdha	250	K	1.057	1.489	1.092	-	-	0.453	0.374	-

Sdha	335	K	1.130	1.179	1.211	1.038	0.695	0.809	0.722	0.046
Sdha	480	K	1.063	1.130	1.211	1.056	0.812	0.778	0.777	0.065
**Sdha**	**547**	**K**	**1.144**	**1.259**	**-**	**-**	**0.851**	**0.726**	**0.656**	**0.045**
Sdha	608	K	-	-	0.038	2.017	1.026	0.919	34.754	-
Sdha	633	K	1.076	1.124	1.169	1.126	0.789	0.769	0.797	0.125
Sdhb	235	K	1.214	1.102	1.120	1.129	0.846	0.653	0.765	0.144
Sdhb	263	K	1.044	-	1.030	1.056	-	-	1.018	-
Slc25a11	73	K	1.016	0.816	0.981	0.900	1.275	1.009	1.132	0.393
Slc25a3	94	K	1.212	1.033	1.359	0.985	0.826	0.655	0.684	0.053
Slc25a3	290	K	1.101	1.196	1.129	1.086	0.806	0.740	0.768	0.080
Slc25a4	33	K	1.049	1.595	0.955	0.814	0.940	0.727	0.689	0.114
Slc25a4	96	K	1.094	1.176	-	0.852	-	0.886	0.766	0.023
Slc25a4	147	K	1.079	1.180	1.182	1.015	0.825	0.776	0.760	0.032
Slc25a4	166	K	-	1.208	-	-	0.834	-	0.690	-
Slc25a4	263	K	1.050	1.230	1.167	0.955	0.812	0.850	0.759	0.015
Slc25a5	147	K	1.019	-	-	0.925	-	-	0.908	-
Sod2	68	K	0.799	1.460	-	0.449	-	1.424	0.829	0.690
Sod2	75	K	1.069	0.962	0.982	1.068	0.944	1.027	1.009	0.872
Sod2	114	K	0.955	1.173	1.222	1.080	0.735	0.874	0.803	0.168
Sucla2	78	K	1.182	1.237	-	1.107	0.787	0.734	0.724	0.132
Sucla2	88	K	0.989	1.250	1.224	0.999	0.960	0.648	0.753	0.132
Sucla2	93	K	-	-	1.083	1.206	0.781	-	0.917	-
Sucla2	108	K	1.047	1.104	1.208	1.186	0.818	0.711	0.808	0.212
Sucla2	116	K	1.072	1.152	1.285	1.072	0.826	0.668	0.731	0.088
Sucla2	139	K	0.848	0.958	1.367	1.531	-	0.463	0.943	0.697
Sucla2	143	K	1.126	1.085	1.225	1.180	0.746	0.716	0.769	0.154
Sucla2	216	K	1.218	1.285	1.216	1.155	0.799	0.436	0.643	0.143
Sucla2	287	K	1.249	-	-	-	0.700	-	0.560	-
Suclg1	54	K	0.973	1.059	-	1.333	-	0.683	0.992	0.871
Suclg1	90	K	1.018	1.257	1.534	1.046	0.649	0.584	0.598	0.068
Suclg1	94	K	0.900	1.056	1.136	1.059	0.971	0.912	0.951	0.594
Suclg1	308	K	0.970	1.261	1.158	1.048	0.833	0.787	0.787	0.108
Suclg2	67	K	1.419	0.644	0.804	1.387	1.062	0.683	1.092	0.755
Tnni3	37	K	1.251	0.922	0.974	1.030	-	0.958	0.948	0.739
Tnni3	121	K	0.745	0.959	1.201	1.449	1.028	0.587	1.055	0.984
Tnni3	165	K	-	1.172	-	0.911	-	-	0.777	-
Tnni3	194	K	-	1.311	-	-	0.747	-	0.570	-
Tnnt2	137	K	-	-	1.231	0.820	-	-	0.666	-
Tnnt2	230	K	1.424	0.992	1.078	0.913	0.860	0.806	0.738	0.064
Tnnt2	266	K	1.440	1.269	1.022	-	-	0.575	0.462	-
Tpm1	37	K	-	1.214	-	1.227	0.550	-	0.732	-
Tpm1	48	K	-	-	0.974	1.047	0.852	-	0.975	-
Tpm1	59	K	1.129	1.055	1.335	1.067	0.833	0.669	0.730	0.097
Tpm1	65	K	0.984	1.056	1.091	1.280	0.711	-	0.954	0.716

Tpm1	77	K	0.381	1.212	1.673	1.149	0.742	-	0.868	0.992
Tpm1	112	K	1.051	0.978	1.300	1.022	0.922	0.774	0.816	0.162
Tpm1	118	K	-	-	1.140	1.229	0.701	0.757	0.786	-
Tpm1	152	K	0.989	1.187	1.100	1.091	0.778	-	0.856	0.328
Tpm1	168	K	1.248	1.080	-	1.066	0.897	0.673	0.755	0.201
Tpm1	189	K	-	1.150	-	-	0.878	-	0.763	-
Tpm1	205	K	1.016	1.088	1.280	0.932	0.837	-	0.784	0.090
Tpm1	213	K	0.980	1.166	1.158	0.974	0.980	0.790	0.831	0.108
Tpm1	217	K	1.026	1.159	-	1.265	0.757	0.750	0.846	0.450
Tpm1	220	K	1.156	1.195	1.214	1.150	0.740	0.656	0.714	0.099
Tpm3	214	K	0.998	1.597	1.088	1.133	0.894	0.629	0.721	0.210
Tpm3	221	K	1.177	1.637	1.140	1.338	0.674	0.522	0.641	0.166
Tpm3	252	K	1.326	0.638	0.601	0.714	1.167	1.219	1.209	0.494
Tpm4	184	K	1.492	1.326	1.383	1.169	0.612	0.535	0.551	0.055
Tpm4	190	K	1.027	0.867	1.398	0.943	0.950	0.861	0.837	0.328
Trap1	128	K	0.807	0.759	0.852	1.280	1.157	1.159	1.487	0.001
Tsfm	75	K	-	0.841	-	1.308	-	-	1.555	-
Ttn	13401	K	1.165	1.189	1.291	0.990	0.806	0.685	0.681	0.024
Ttn	16660	K	1.083	1.169	1.012	1.008	0.920	0.854	0.852	0.065
Ttn	17332	K	1.255	1.097	-	1.033	0.692	-	0.733	0.261
Ttn	33769	K	0.726	-	-	1.253	-	-	1.726	-
Tufm	91	K	1.058	1.115	1.110	1.061	0.754	-	0.829	0.217
Uqcrb	12	K	1.050	1.180	1.333	1.033	0.744	0.737	0.706	0.052
**Uqcrc2**	**92**	**K**	**1.471**	**1.135**	**1.154**	**0.952**	**0.668**	**0.719**	**0.622**	**0.025**
Uqcrc2	375	K	-	1.438	-	1.081	-	0.535	0.562	-
Uqcrfs1	101	K	1.227	-	1.008	1.070	-	0.656	0.772	0.395
Uqcrh	40	K	-	-	1.244	1.181	0.870	0.814	0.768	-
Vdac3	63	K	1.141	0.958	0.980	0.962	-	-	0.937	-
α-MHC	43	K	0.821	1.233	-	1.025	0.884	0.971	0.935	0.782
α-MHC	58	K	1.234	1.251	1.077	-	0.745	0.847	0.670	0.014
α-MHC	67	K	1.084	1.221	1.297	1.072	0.781	0.656	0.697	0.068
α-MHC	72	K	1.102	1.028	1.136	1.148	0.821	0.816	0.853	0.215
α-MHC	279	K	1.138	1.200	1.123	0.934	-	0.810	0.756	0.018
α-MHC	406	K	0.886	1.306	1.008	1.101	-	0.870	0.924	0.700
α-MHC	550	K	1.214	1.268	1.018	0.797	-	-	0.683	-
α-MHC	552	K	1.214	1.268	1.018	0.797	-	-	0.683	-
α-MHC	560	K	-	1.296	-	-	0.747	-	0.576	-
α-MHC	566	K	1.111	1.191	1.203	1.006	0.762	0.819	0.738	0.023
α-MHC	642	K	1.216	1.223	1.080	1.054	0.774	0.750	0.733	0.050
α-MHC	659	K	1.180	1.178	-	-	0.939	0.734	0.709	0.104
α-MHC	665	K	1.041	-	-	-	1.140	0.675	0.872	-
α-MHC	681	K	-	1.392	-	0.703	0.760	1.055	0.603	-
α-MHC	759	K	1.155	1.142	1.001	0.948	0.898	0.905	0.834	0.021
α-MHC	855	K	-	0.961	1.133	1.103	0.886	0.857	0.906	0.456

α-MHC	867	K	0.993	1.188	1.144	1.026	0.838	0.870	0.822	0.076
α-MHC	912	K	1.064	1.194	1.033	0.995	0.901	0.863	0.838	0.045
α-MHC	914	K	1.180	1.388	0.932	1.033	0.845	0.717	0.741	0.131
α-MHC	941	K	1.036	1.290	1.030	1.032	0.988	0.686	0.806	0.205
α-MHC	944	K	0.973	0.942	0.792	0.899	0.950	1.382	1.194	0.339
α-MHC	953	K	1.255	0.975	0.698	0.862	0.921	1.263	1.040	0.809
α-MHC	954	K	1.299	0.974	0.964	0.999	0.859	0.942	0.865	0.270
α-MHC	1024	K	0.962	-	-	-	1.067	0.818	0.980	-
α-MHC	1085	K	1.046	1.196	1.154	1.124	0.777	0.780	0.789	0.124
α-MHC	1167	K	0.950	-	-	1.072	-	-	1.128	-
α-MHC	1175	K	1.297	1.177	1.057	0.956	0.859	0.745	0.725	0.026
α-MHC	1197	K	1.454	1.343	1.059	0.977	0.776	0.546	0.596	0.051
α-MHC	1244	K	-	1.471	0.877	0.977	0.760	-	0.740	0.438
α-MHC	1249	K	1.258	1.345	1.094	0.966	0.824	0.639	0.657	0.033
α-MHC	1264	K	-	-	0.912	1.106	-	-	1.213	-
α-MHC	1281	K	1.096	1.471	1.067	1.053	0.796	0.639	0.685	0.093
α-MHC	1307	K	1.214	1.295	0.957	0.945	0.869	0.802	0.755	0.056
α-MHC	1318	K	1.250	0.533	1.173	1.094	1.013	-	1.069	0.732
α-MHC	1326	K	0.976	1.204	-	-	0.912	-	0.837	-
α-MHC	1356	K	1.227	0.660	-	1.351	-	0.792	1.136	0.766
α-MHC	1365	K	-	1.253	1.157	0.804	0.915	0.858	0.713	0.009
α-MHC	1376	K	1.117	1.413	0.941	1.112	0.899	0.608	0.755	0.234
α-MHC	1392	K	1.052	1.218	1.096	0.980	-	0.845	0.813	0.078
α-MHC	1412	K	0.834	-	-	-	1.181	0.806	1.191	-
α-MHC	1418	K	1.111	1.260	0.966	0.905	0.945	0.866	0.814	0.068
α-MHC	1446	K	1.498	1.260	0.327	1.146	0.945	0.844	0.951	0.802
α-MHC	1461	K	0.892	1.759	0.890	1.114	0.867	0.594	0.727	0.366
α-MHC	1474	K	1.370	1.261	1.056	1.025	0.814	0.597	0.661	0.068
α-MHC	1487	K	0.715	1.312	0.786	1.098	1.145	-	1.196	0.441
α-MHC	1530	K	0.920	1.046	1.077	0.903	1.142	0.901	0.968	0.713
α-MHC	1533	K	1.357	1.357	-	0.912	0.782	0.639	0.573	0.024
α-MHC	1571	K	1.494	0.824	0.941	1.004	1.051	0.714	0.850	0.546
α-MHC	1577	K	0.975	-	1.024	1.052	0.977	0.860	0.963	0.641
α-MHC	1581	K	1.175	1.182	1.003	1.133	0.782	0.796	0.807	0.161
α-MHC	1643	K	-	1.240	1.109	1.032	0.835	0.772	0.749	0.090
α-MHC	1702	K	1.279	1.211	1.088	0.941	0.809	0.773	0.705	0.010
α-MHC	1729	K	0.864	1.706	1.477	0.731	0.561	0.859	0.532	0.066
α-MHC	1759	K	1.240	1.232	1.086	0.781	0.919	0.828	0.711	0.006
α-MHC	1773	K	1.370	1.296	-	1.083	-	0.419	0.563	0.288
α-MHC	1793	K	1.074	1.133	0.993	0.962	-	0.998	0.919	0.202
α-MHC	1833	K	1.063	1.102	1.193	1.315	0.622	0.789	0.812	0.313
α-MHC	1840	K	1.054	-	1.047	1.023	-	-	0.974	-
α-MHC	1850	K	0.712	1.380	0.920	1.147	1.004	0.848	0.996	0.909
α-MHC	1860	K	1.197	1.297	0.998	1.035	0.879	0.682	0.743	0.102

α-MHC	1872	K	1.076	1.128	1.051	1.003	-	-	0.924	-
**α-MHC**	**1897**	**K**	**1.695**	**1.141**	**-**	**0.600**	**0.810**	**0.802**	**0.520**	**0.045**
α-MHC	1900	K	1.141	1.162	1.198	1.159	0.768	0.669	0.742	0.120
α-MHC	1921	K	1.099	1.194	1.133	0.959	0.809	0.880	0.773	0.009

Of all 483 overlapping lactylated modified sites, 14 lactylated modified sites (marked in red) were significantly differentially expressed between the control group and the Ang II-induced heart failure group using cut offs of fold change > 1.5/ fold change < 0.67, and *P* value < 0.05.
