# Supplementary material for: α-myosin heavy chain lactylation maintains sarcomeric structure and function and alleviates the development of heart failure
Source: Cell Res. 2023 Jul 13;33(9):679–98. doi: 10.1038/s41422-023-00844-w (PMC10474270; doi:10.1038/s41422-023-00844-w)
Supplement: Supplementary file 11 — Supplementary information, Table S3 [file 41422_2023_844_MOESM11_ESM.pdf]

Supplementary information, Table S3 Plasmids and siRNA used in this study

PLASMID	SPECIES	TAG	VECTOR	IDENTIFIER	SOURCE
α-MHC WT	Human	1XMyc	GV172	GOSE0270372	GeneChem (China)
α-MHC K1897R	Human	1XMyc	GV172	GOSE0270369	GeneChem (China)
α-MHC ΔTMPIT	Human	1XMyc	GV172	GOSE0324668	GeneChem (China)
α-MHC ΔClass II domain	Human	1XMyc	GV172	GOSE0324663	GeneChem (China)
α-MHC ΔSH3	Human	1XMyc	GV172	GOSE0324662	GeneChem (China)
α-MHC ΔSPEC	Human	1XMyc	GV172	GOSE0324664	GeneChem (China)
α-MHC ΔmmCoA	Human	1XMyc	GV172	GOSE0324665	GeneChem (China)
α-MHC ΔMIT-CorA	Human	1XMyc	GV172	GOSE0324666	GeneChem (China)
LDHA	Human	1XMyc	GV172	GOSE0338080	GeneChem (China)
Titin A80-82	Human	6XHis	GV172	GOSE0301069	GeneChem (China)
Titin I1065-1108	Human	6XHis	GV172	GOSE0301072	GeneChem (China)
Titin A84-86	Human	6XHis	GV172	GOSE0301071	GeneChem (China)
Titin A77-78	Human	6XHis	GV172	GOSE0301036	GeneChem (China)
SIRT1	Human	1XFlag	Pcdna3.1		
SIRT2	Human	1XFlag	Pcdna3.1		
SIRT3	Human	1XFlag	Pcdna3.1	A gift from Prof.Chuangui Wang	
SIRT4	Human	1XFlag	Pcdna3.1	(China Medical University,	
SIRT5	Human	1XFlag	Pcdna3.1	Shenyang, China)	
SIRT6	Human	1XFlag	Pcdna3.1		
SIRT7	Human	1XFlag	Pcdna3.1		
GCN5	Human	1XMyc		A gift from Prof.Qunying Lei	
P300	Human	1XFlag		(Shanghai Medical College,	
CBP	Human	1XFlag		Shanghai, China)	
PCAF	Human	1XFlag		A gift from Prof. Weiguo Zhu (Shenzhen University, Shenzhen, China)	

siRNA	SPECIES	TARGET SEQUENCE	SOURCE
siLDHA 001	Human	GAGGTAGCTTGGAACCTAA	RIBOBIO (China)
siLDHA 002	Human	GCCACTATGTGGTAGTTAA	RIBOBIO (China)
siLDHA 003	Human	GACAGAGCCTCAGATTACA	RIBOBIO (China)
