# Supplementary material for: α-myosin heavy chain lactylation maintains sarcomeric structure and function and alleviates the development of heart failure
Source: Cell Res. 2023 Jul 13;33(9):679–98. doi: 10.1038/s41422-023-00844-w (PMC10474270; doi:10.1038/s41422-023-00844-w)
Supplement: Supplementary file 12 — Supplementary information, Table S4 [file 41422_2023_844_MOESM12_ESM.pdf]

Supplementary information, Table S4 Antibodies and reagents used in this study

ANTIBODY	IDENTIFIER	SOURCE
Anti-α-MHC	Cat#sc-32732	Santa (USA)
Anti-LDHA	Cat#19987-1-AP	Proteintech(China)
Anti-Tubulin	Cat#11224-1-AP	Proteintech (China)
Anti-α-MHC K1897 Lactyl Lysine	Cat#CL030202	PTMBIO(China)
Anti-Pan Kla	Cat#PTM-1401RM	PTMBIO(China)
Anti-Cleaved-PARP1	Cat#5625S	Cell Signaling Technology (USA)
Anti-Cleaved-Caspase3	Cat#19677-1-AP	Proteintech (China)
Anti-Flag	Cat#GNI4110-FG-M	GNI (Japan)
Anti-Myc	Cat#GNI4110-MC-M	GNI (Japan)
Anti-His	Cat#GNI4110-HS-M	GNI (Japan)
Anti-P300	Cat#70088	Cell Signaling Technology (USA)
Anti-P300	Cat#AF5360	Affinity Biosciences (China)
Aniti-BNP	Cat#DF6902	Affinity Biosciences (China)
Anti-SIRT1	Cat#8469	Cell Signaling Technology (USA)
Anti-SIRT1	Cat#13161-1-AP	Proteintech (China)
Anti-α-SMA	Cat#55135-1-AP	Proteintech(China)
Anti-Col-1	Cat#67288-1-Ig	Proteintech (China)
Anti-Titin	Cat#sc-271946	Santa (USA)
Normal rabbit IgG	Cat#A7016	Beyotime Biotechnology (China)
Normal mouse IgG	Cat#A7028	Beyotime Biotechnology (China)
**REAGENT**		
Masson's Trichrome Stain Kit	Cat#G1340	Solarbio (China)
Eosin Y solution	Cat#G1100	Solarbio (China)
Mayer Hematoxylin solution	Cat#G1080	Solarbio (China)
WGA,Fluorescein	Cat#FL-1021	Vector laboratories (USA)
Galloflavin (LDHA inhibitor)	Cat#HY-W040118	MedChemExpress (USA)
C646(P300 inhibitor)	Cat#HY-13823	MedChemExpress (USA)
CTB (P300 activator)	Cat#HY-134964	MedChemExpress (USA)
EX527 (SIRT1 inhibitor)	Cat#A4181	APExBio (USA)
SRT1720 (SIRT1 activator)	Cat#A4180	APExBio (USA)
Lactate sodium	Cat#HY-B2227B	MedChemExpress (USA)
VB124	Cat#S9929	Selleck (USA)
CheKine™ Lactate Assay Kit	Cat#KTB1100	Abbkine (China)
Fetal Bovine Serum	Cat#FB15015	Clark (Australia)
High-glucose DMEM	Cat#01-052-1A	Biological Industries (Israel)
Lipofectamine 3000	Cat#L3000015	Invitrogen (USA)
HiGene	Cat#c1506	Applygen (China)
PageRuler™ Prestained Protein Ladder	Cat#26616	Thermo Scientific (USA)
PageRuler™ Multicolor High Range Protein Ladder	Cat#26625	Thermo Scientific (USA)
jetPRIME	Cat#PT-114-15	Polyplus (France)
Ang II	Cat#HY-13948	MedChemExpress (USA)

AlZET Osmotic Pumps	Cat#2002	DURECT Corporation (Cupertino, CA)
Protein A/G magnetic beads	Cat#B23202	Bimake(USA)
Tris-EDTA Antigen Retrieval Solution,10×(ph=9.0)	Cat#C1038	Solarbio (China)
DAB plus	Cat#DAB-2031	MXB Biotechnologies (China)
UltraSensitiveTM SP(Mouse/Rabbit) IHC Kit	Cat#KIT-9710	MXB Biotechnologies (China)
